# Risk of infection in patients with lymphoma receiving rituximab: systematic review and meta-analysis

**DOI:** 10.1186/1741-7015-9-36

**Published:** 2011-04-12

**Authors:** Simone Lanini, Aoife C Molloy, Paul E Fine, Archibald G Prentice, Giuseppe Ippolito, Christopher C Kibbler

**Affiliations:** 1 National Institute for Infectious Diseases, INMI-Lazzaro Spallanzani Via Portuense, 292 00149 Rome, Italy; 2 Department of Medical Microbiology, Royal Free Hospital, Pond Street, London NW3 2QG, UK; 3 London School of Hygiene and Tropical Medicine, Keppel Street, London WC1E 7HT, UK; 4 Department of Haematology, Royal Free Hospital, Pond Street, London NW3 2QG, UK

## Abstract

**Background:**

The addition of Rituximab (R) to standard chemotherapy (C) has been reported to improve the end of treatment outcome in patients affected by CD-20 positive malignant lymphomas (CD20+ ML). Nevertheless, given the profound and prolonged immunosuppression produced by R there are concerns that severe infections may arise. A systematic review and meta-analysis were performed to determine whether or not the addition of R to C may increase the risk of severe infections in adults undergoing induction therapy for CD20+ ML.

**Methods:**

Only randomised controlled trials comparing R-C to C standard alone in adult patients with CD20+ ML were included. Meta-analysis was performed on overall incidence of severe infection, risk of dying as the consequence of infection, risk of febrile neutropenia, risk of severe leucopenia, risk of severe granulocytopenia and overall response assuming a fixed effect model. Heterogeneity was investigated, if present and I^2 ^>20%, according to several predefined baseline characteristics of the study populations.

**Results:**

Several relevant results have emerged. First, the addition of R to standard C does not increase the overall risk of severe infections (RR = 1.00; 95% CI 0.87 to 1.14) nor does it increase the risk of dying as a consequence of infection (RR = 1.60; 95% CI 0.68 to 3.75). Second, we confirmed that the addition of R to standard C increases the proportion of overall response (RR = 1.12; 95% CI 1.09 to 1.15), but it also increases the risk of severe leucopenia (RR = 1.24; 95% CI 1.12 to 1.37) and granulocytopenia (RR = 1.07; 95% CI 1.02 to 1.12).

**Conclusions:**

R-C is superior to standard C in terms of overall response and it does not increase the overall incidence of severe infection. However, data on special groups of patients (for example, HIV positive subjects and HBV carriers) are lacking. In our opinion more studies are needed to explore the potential effect of R on silent and chronic viral infections.

## Background

CD20 positive (CD20+) malignant lymphomas (ML) are a group of potentially lethal neoplasms with an incidence rate of approximately 19 cases per 100,000 person-years in Europe and represent one of the leading causes of cancer in adults [1]. The last revision of the World Health Organization (WHO) "Classification of Tumours of Haematopoietic and Lymphoid Tissues" identified 40 CD-20+ ML subtypes [2]. From a practical point of view the different histological subtypes can be grouped according to their clinical features, into aggressive, potentially curable, and indolent, as yet incurable. The majority of CD20+ ML in adults are indolent lymphomas and include different histological subtypes such as chronic lymphocytic leukemia/small lymphocytic lymphoma (CLL), follicular lymphoma (FL), marginal zone lymphoma (MZL) and lymphoplasmacytic lymphoma (LPL). Aggressive lymphomas are less common in adults and include diffuse large B-cell lymphomas (DLBCL) and all HIV-associated lymphomas.

Effective multi-drug chemotherapy (C) protocols for CD20+ ML have been available for the last 30 years with variable results in indolent and aggressive ML but protocols have been changing recently with the introduction of rituximab (R) [3]. R is a chimerical anti-CD20 monoclonal antibody (MoAb) with activity against normal and malignant B-cells expressing the cell-surface molecule CD20. Recent systematic reviews provide evidence that, in comparison to C alone, the combination of R and C (R-C) may increase the remission both in indolent [4] and aggressive CD20+ ML [5,6]. However, given the profound and prolonged immunosuppression produced by R, there are concerns that infections may arise [7].

In studies of HIV positive (HIV+) patients with ML there is evidence of an increased risk of infections when R is added to C, in particular for patients with CD4 counts less than 50 cells/ml [8]. In pooled results from three phase II trials of patients with ML receiving R-C, 31% of patients developed severe infections, compared with 20% incidence reported in prior studies with similar C protocols [9]. In the only phase III trial published so far, comparing R-C to C in HIV-associated ML, the mortality due to infection was significantly higher in R-C than in standard C: 14% and 2% respectively (*P *= 0.035) [8].

A meta-analysis comparing R maintenance therapy with observation in HIV negative subjects indicated that the risk of infections in the intervention was double that in the control arm [10].

Case reports and case series also suggest that R increases the risk of viral infections [11]. Potentially lethal reactivations of hepatitis B virus (HBV) [12] may occur after R therapy both in patients with resolved HBV (anti-HBsAg+) and in those with isolated HBV core antibody positivity (anti-HBc+) [13,14]. These observations are strengthened by other reports which show that protective antibody against HBV (anti-HBsAg) may diminish, or even vanish, soon after R administration in some patients [15,16] and that patients receiving anti-HBV prophylaxis during R-C regimens may experience no reactivation of their latent HBV infection [14-17].

Problems with other viruses have also been reported in association with R-containing regimens. Severe herpes virus reactivation (for example, cytomegalovirus [18-20] and varicella zoster [20,21]) has been reported in several patients. Potentially lethal enteroviral encephalitis has been described after R-C in patients with ML [22-24]. Papovavirus infections have been linked to R-containing therapies in rare case reports. JC virus infection was reported in patients undergoing autologous hematopoietic stem cell transplantation soon after peri-transplant R was added [25]. BK virus-associated leukoencephalopathy developed in a single patient soon after receiving R, although this patient also had a complicated history of prior treatment for Hodgkin's disease [26]. Anecdotal reports have also indicated that Parvovirus B19 with pure red cell aplasia [27] and West Nile virus [28] may be linked to R-containing protocols.

R is also associated with impaired immunity against non-viral pathogens such as *Babesia microti *[29] and *Pneumocystis jirovecii *[30].

Infections are among the most important causes of morbidity and mortality in patients suffering from cancer. Although R-C is associated with a treatment outcome superior to standard C, it is not clear whether or not the addition of R to standard C increases the risk of infections. The main objective of this review is to explore this. To do so we examine the evidence on infections from RCTs enrolling adults with CD20+ ML who undergo R-C compared with identical C alone. Other endpoints included in the review are the risk of specific clinical presentation of infections, risk of death due to infection, risk of severe leucopenia, risk of severe granulocytopenia, risk of severe lymphopenia and overall response to therapy.

## Methods

The review is reported according to the new "*Preferred reporting items for systematic reviews and meta-analyses: the PRISMA statement*" guidelines [31,32] (see Additional file 1).

### Eligibility criteria

We included only peer-reviewed studies published in English, Spanish, French, German and Italian up to 31 July 2010.

The following inclusion criteria were used: being an RCT; enrolling adults with CD20+ ML; and identical C regimens in both arms with regard to type of drug(s), dosages and number of administrations (studies which included more than one C regimen were excluded provided that disaggregated data for patients receiving the different Cs were available).

Exclusion criteria included the following: studies not reporting data about infection outcome; studies including non-lymphoma patients; studies without identical C in both arms; studies about maintenance purging and sequential treatment; studies without an R-free arm; non-randomized studies; studies including other MoAb in addition to or instead of R; studies in children (aged 16 or less); studies published in languages not mentioned above; and published editorials/reviews/letters/comments.

### Information source and search strategy

An electronic search was performed in three different databases (PubMed; Embase; Cochrane Controlled Trials Register) to reduce publication bias

Different sets of key words, Mesh terms, truncation and filters for trials were used and the complete text for each string is provided in Additional file 2.

Further information was sought by e-mailing the authors to clarify uncertainty and omissions. In addition, we searched and analysed systematic reviews with similar inclusion criteria published over the last five years to determine if they reported additional studies or unpublished data. The references of all included studies and systematic reviews were also searched to find additional eligible studies

### Study selection

Study selection was done by two independent reviewers (SL and AM). To guarantee transparency throughout the selection process, excluded papers were ranked according to the list of exclusion criteria. A single paper was either included or discarded if both reviewers had reached the same decision independently. For discordant decisions papers were assessed again by both reviewers and a consensus decision was taken.

### Data collection and data items

Data about baseline population characteristics, type of intervention, outcome and study design were collected.

Population baseline data included the number of subjects randomized in each arm; participants' mean age in each arm; the proportion of different ML histological subtypes in each arm (this is according to WHO's "Classification of Tumours of Haematopoietic and Lymphoid Tissues [2]); line of treatment (that is, untreated or refractory); HIV status of participants; and HBV status of participants.

Intervention data included the type of drugs and dosage used in the C; the scheduled number of C cycles; and the use of any other adjuvant therapies, such as antivirals, antibiotics, granulocyte colony-stimulating factor (G-CSF) or radiotherapy; dosage and number of R courses in the intervention arm.

Only the events which occurred during treatment were considered. Grades of adverse events were according to the NCI Common Terminology Criteria for Adverse Events Version 3.0 [33] and remission was defined according to the international definition for response criteria [34]. Outcome data were collected for: incidence of grade 3 and 4 infections [33] during treatment; incidence of different types of infections according to their clinical diagnosis, as reported by the authors; incidence of febrile neutropenia; the incidence of infection-related death; and the incidence of grade 3 or 4 leucopenia/granulocytopenia/lymphopenia.

We reviewed method(s) used to generate the allocation sequence, method(s) used to conceal the allocation sequence, measure(s) used to blind study participants and personnel from knowledge of which intervention a participant received, type of analysis, data about attrition, early stop of studies and study funding.

### Assessment of risk of bias

To assess potential within-study risk of bias we considered several tools as reported below.

The methods used to generate the allocation sequence were adequate if authors referred to a random number table or stratification or a computer to generate a random number or minimization; according to Schulz *et al. *[35], we considered manual randomization, such as shuffling envelopes or throwing dice, inadequate.

The methods used to conceal allocation sequence to operator were adequate if authors referred to central allocation or sequentially numbered drug containers of identical appearance or sequentially numbered opaque sealed envelopes.

The methods used to blind study participants and personnel were adequate if the assessors of outcome and patients were blinded and an appropriate placebo, that is, an inactive intravenous (IV) compound in spite of R, was used.

The methods used to manage incomplete data reports and type of analysis were adequate if intent-to-treat analysis was undertaken without attrition or intent-to-treat analysis was undertaken and overall attrition was lower than 20% with similar attrition proportions in both arms.

The presence of potential conflict of interests was adequate if the study was mainly sponsored by government or any other organization without a direct link with R manufacturer and individual potential conflicting interests were disclosed.

The occurrence of early termination of the study was adequate if it did not occur.

A study was considered at low risk of bias if all the criteria were met and reported, at high risk of bias if one or more criteria were not met, or at unclear risk of bias if data for one or more items were not reported.

### Assessing the quality of a body of evidence

We assessed the quality of the evidence supporting the meta-analysis results for each outcome according to the *Grades of Recommendation, Assessment, Development and Evaluation Working Group *approach (GRADE) as reported in the Cochrane Handbook for Systematic Reviews of Interventions [36,37].

In brief, the quality of evidence for each outcome was ranked according to four levels of quality; that is, high, moderate, low and very low. This was done with a two-step approach. First, we considered all outcomes as supported by high quality evidence since we included only RCTs. Then we downgraded the level of evidence according to four criteria: 1. within-study risk of bias was present if less than two studies were at low risk of bias; 2. imprecision of results was present if wide overall 95% confidence interval (95% CI) was present (according to GRADE guidance a confidence interval is wide when "*the estimate is consistent with conflicting recommendations*") [36]; 3. indirectness of evidence was present if indirect comparison between R-C and C was done; and 4. risk of publication bias was present if funnel plots showed relevant asymmetry (see below).

The body of evidence was considered either of high quality (if no criterion was present), moderate quality (if one criterion was present), low quality (if two criteria were present) or very low quality (if three or more criteria were present).

### Statistical methods

All outcomes were considered as binary variables. Risk ratio (RR) was used as a measure of association according to the formula:

A fixed-effect model using the inverse-variance method was considered in all meta-analyses.

RR standard error (SE) was calculated according to the formula:

Where sqr = square root, Ai = events in the intervention arm in i^th ^study, Ci = events in the control arm in i^th ^study, N1i = participants in intervention arm in i^th ^study and N2i = participants in control arm in i^th ^study. Exponential form was always reported.

95% CI were calculated according to the formula: 95% CI = RR ± 1.96 SE

Meta-analysis was performed for outcomes reported in two or more studies. Meta-analyses of risk of infections, leucopenia and granulocytopenia included only studies reporting events according to the NCI grade (and only grade 3 and 4 events where considered for the analysis). Data about countable outcomes (that is, outcomes which may occur more than once in the same subject, such as infection, granulocytopenia, leucopenia and lymphopenia) were included in a meta-analysis only when patient level data (that is, risks or rates) were provided (we did not include studies reporting only the proportion of cycles in which events occurred).

In meta-analyses with at least four studies a funnel plot was generated and visually assessed by the help of Egger's graphical test [38,39]. We did not perform a formal statistical test for significance since the power and sensitivity of such tests is not well established when RR is used as the measure of association [37].

We considered heterogeneity negligible if I-squared was <20.0%, moderate if I-squared was between 20.0% and 49.9% and strong if I-squared was ≥50.0%. For meta-analyses with moderate/strong heterogeneity, potential causes were explored by subgroup analysis for binary dichotomous covariates, that is: ML type (indolent/aggressive), line of treatment (untreated/refractory) and HIV status (positive/negative). A simple meta-regression model was used for continuous covariates, that is: mean age and minimum/maximum number of R cycles scheduled. In subgroup analyses with persisting heterogeneity, Der Simonian and Laird random-effect RR estimates were provided as terms of comparison. Chi-square *P*-value for heterogeneity was also provided in all fixed-effect models.

Sensitivity analysis was undertaken to evaluate the robustness of findings according to the relevant features which may affect within-study risk of bias (that is, type of funding; whether the study stopped early; methods used to manage incomplete data and randomization (this latter was adequate if both sequence generation and allocation concealment were adequate)).

STATA 11.1 (StataCorp Texas 77845 USA) package was used for the analysis and to generate forest plots and funnel plots.

## Results

The literature search identified 729 unduplicated papers. Of these, 674 were excluded directly by reading the title and abstract and a further 39 were discarded after full evaluation of the text version. The remaining 16 papers comprising 17 RCTs for a total of 5,259 patients, were included (one paper, Pfreundschuh 2008, was divided into two RCTs according to the different R-C/C regimens). A summary table with the results of study selection, references of all included papers and the list of excluded papers are reported in Additional files 3, 4 and 5 respectively. All the data reported are those published, no additional data were provided by authors.

### Study characteristics

Of the 17 RCTs, 16 were phase III RCTs to assess efficacy and 1 was a phase II RTC to assess toxicity.

Five RCTs enrolled 150 or less patients, six between 150 and 400 patients and six more than 400 patients. Mean age of participants was between 50.0 and 69.2 years (quartiles 55.5, 61.5, 68.0). Nine RCTs enrolled patients with indolent lymphoma (FC, MZL, CLL, ML and LPL) and eight RCTs enrolled patients with aggressive lymphoma (almost all DLBCL). Twelve RCTs enrolled only previously untreated subjects and five only refractory or relapsed patients. HIV status was reported in 13 studies (12 HIV negative and 1 HIV positive) and 4 studies did not explicitly report the HIV status of participants but it is likely they included only HIV negative subjects. HBV status was reported in seven studies, three RCTs included only HBsAg negative (the authors specified "absence of chronic hepatitis") and four RCTs included only anti-HBc negative (the authors specified "negative HBV serology"). Table 1 shows the baseline characteristics of the study populations.

**Table 1:** Studies according to the study population baseline characteristics.

Characteristics of study populations							
**Study**	**Number ^a^**		**Mean age **	**Line of treatment **	**Type of lymphoma**	**HIV status **	**HBV status **
						
	**Intervention**	**control**					

Aviles 2007a	102	102	69.2	untreated	aggressive	neg.	anti-HBc -
Aviles 2007b	98	98	59.7	refractory	aggressive	neg.	anti-HBc -
Aviles 2010	47	53	50.0	refractory	aggressive	neg.	anti-HBc -
Buske 2009	36	33	60.5	untreated	indolent	neg.*	NR
Coiffier 2002	202	197	69.0	untreated	aggressive#	neg.	HBsAg -
Eve 2009	78	78	63.5	untreated	indolent	neg.	anti-HBc -
Forstpointner 2004	66	62	62.5	refractory	indolent	neg.*	NR
Habermann 2006	267	279	69.5	untreated	aggressive	neg.	NR
Herold 2007	105	96	58.5	untreated	indolent	neg.	NR
Hiddemann 2005	223	205	55.5	untreated	indolent	neg.*	NR
Kaplan 2005	99	51	42.7	untreated	aggressive	pos.	NR
Lenz 2005	62	60	61.5	untreated	indolent	neg.*	NR
Marcus 2005	162	159	52.5	untreated	Indolent §	neg.	HBsAg -
Pfreundschuh 2008a	304	305	68.5	untreated	aggressive#	neg.	NR
Pfreundschuh 2008b	306	307	68.0	untreated	aggressive#	neg.	NR
Robak 2010	276	276	62.5	refractory	indolent	neg.	HBsAg-
van Oers 2006	234	231	54.5	refractory	indolent	neg.	NR

a) number of patients included either in the control and intervention arm; NR = not reported; § this includes 1.2% undefined lymphomas; # this includes 6 to 20% proportion of indolent or undefined; * HIV status not specified and presumed as negative.

Eight different protocols were associated with R as shown in Additional file 6. R was always administered at 375 mg/m^2^, given either the day before or the same day of C. Number of C cycles, number of R administrations and other adjuvant therapies in each individual study are reported in Table 2. Minimum and maximum number of R cycles varies between three and eight (modal value six; median value six) and four and eight (modal value eight; median value six), respectively.

**Table 2:** Studies according to the type of intervention.

Intervention							
**Study**	**Intervention arm ^a^**			**Adjuvant therapies ^b^**			
		
	**Type of CHT **	**N. Cycles **	**R courses **	**G-CSF**	**AI prophylaxis**	**RTX**	**Other**

Aviles 2007a	CEOP	6	6	Discretionary	NR	No	No
Aviles 2007b	CEOP	6	6	Not reported			
Aviles 2010	ESHAP	6	6	To all	No	No	No
Buske 2009	CHOP 21	4 to 8	4 to 8	Not reported			
Coiffier 2002	CHOP 21	8	8	Discretionary	NR	No	No
Eve 2009	FC	8	8	NR	PCP + VZV	No	No
Forstpointner 2004	FCM	4	4	Not reported			
Habermann 2006	CHOP 21	6 to 8	4 to 5	Discretionary	NR	No	M
Herold 2007	MCP	8	8	Not reported			
Hiddemann 2005	CHOP 21	6 to 8	6 to 8	Not reported			
Kaplan 2005	CHOP 21	3 to 6	3 to 6	To all	PCP	Discretionary	M + ART
Lenz 2005	CHOP 21	6	6	Not reported			
Marcus 2005	CVP	8	8	Not reported			
Pfreundschuh 2008a	CHOP 14	6	8	To all	No	No	Pre-O
Pfreundschuh 2008b	CHOP 14	8	8	To all	No	No	Pre-O
Robak 2010	FC	6	6	Discretionary	No	No	No
van Oers 2006	CHOP 21	3 to 6	3 to 6	Not reported			

Detailed protocol reported in additional file 6. a) This describes the type of therapy for intervention arm (patients in control arm received the same therapy apart from rituximab); b) This includes all reported non randomized therapies. AI prophylaxis = anti-infective prophylaxis; ART = antiretroviral therapy; C = chemotherapy; -CSF = granulocyte colony stimulating factor; GRTX = radio therapy; M = meningeal prophylaxis with methotrexate; N. cycles = number of cycles of scheduled therapy; NR = not reported; PCP = prophylaxis against *P. jaroviecy*; Pre-O = pre-treatment with single dose 1 mg vincristine and 100 mg prednisone orally for seven days; R course = number of cycles of scheduled rituximab course; VZV = prophylaxis against Varicella zoster virus

Data for binary outcomes (that is: overall response and deaths from infection) were reported as the number or proportion of patients experiencing the event. Countable outcomes data were reported in two different ways: 13 studies reported the number or proportion of patients experiencing at least one event throughout the therapy and 4 other studies analysed countable outcomes as the number or proportion of cycles of therapy in which events occurred at least once. No study clearly reported counts as rates or reported the absolute numbers of events and time at risk during therapy in either arm (see Additional file 7 for details about outcome reporting).

### Risk of bias within the studies

**Figure 1:**
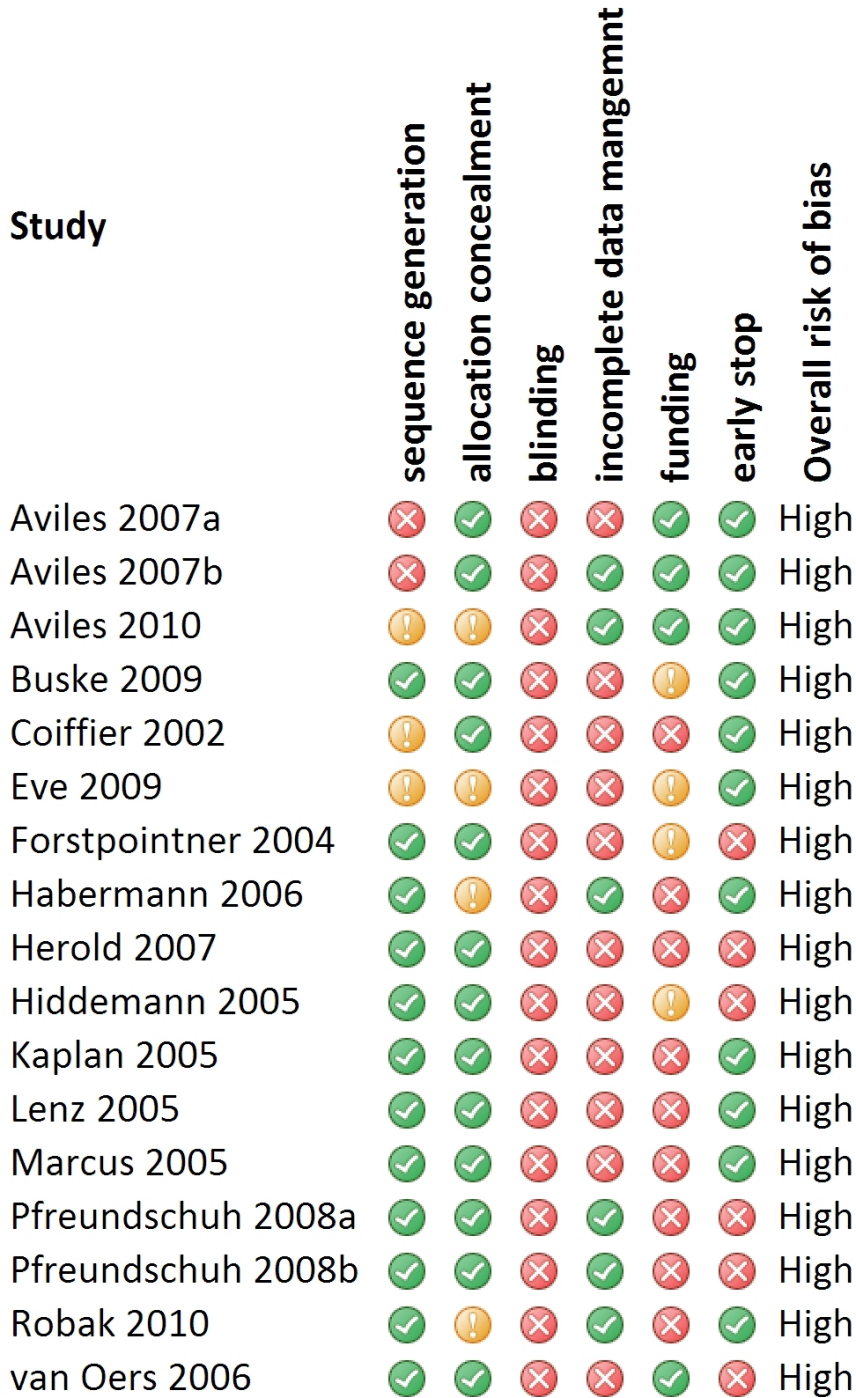
**Within-study risk of bias**. This figure shows the summary of within-study risk of bias. Green icons indicate methodological features adequately undertaken and reported, yellow icons indicate methodological features unclearly reported, red icons indicate methodological features inadequately undertaken.

All the studies were considered to be at high risk of bias. Figure 1 reports the summary of the within-study risk of bias analysis (Additional file 8 reports details of risk of bias analysis).

### Results of the meta-analyses

**Figure 2:**
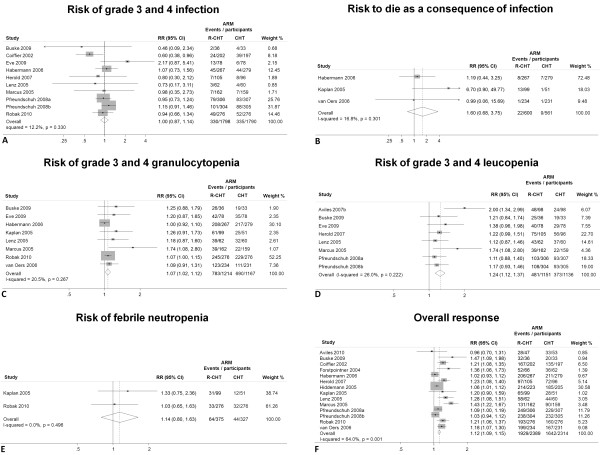
**Fixed-effect model meta-analysis**. This figure shows the fixed-effect model meta-analysis of the six outcomes included in the meta-analyses. **A) **Studies for risk of grade 3 and 4 infections (as shown by the diamond at the bottom) show no evidence for increased risk in R-C vs. C arm and negligible heterogeneity (I-squared <20%). **B) **Three studies for risk of death as a consequence of infection; no evidence for increased risk in R-C vs. C arm and negligible heterogeneity (I-squared <20%) was found. **C)**. Eight studies for risk of grade 3 and 4 granulocytopenia; significant evidence for increased risk in R-C vs. C was found, although moderate evidence of heterogeneity (I-squared = 20% to 50%) was present. **D) **Eight studies for risk of grade 3 and 4 leucopenia; significant evidence for increased risk in R-C vs. C was found, although moderate evidence of heterogeneity (I-squared = 20% to 50%) was present. **E)**. Two studies for risk of febrile neutropenia; no evidence for increased risk in R-C vs. C arm and negligible heterogeneity (I-squared <20%) was found. **F) **Fourteen studies for overall response; overall response was significantly better in R-C vs. C, although strong evidence of heterogeneity (I-squared = >50%) was present. RR = risk ratio, 95% CI = 95% confidence interval.

Figure 2 shows the forest plots with results of the meta-analyses. In particular, pooled RRs did not indicate increased risk in patients receiving R-C compared to those receiving C for infections (RR = 1.00; CI 95% = 0.87 to 1.14, *P *= 0.943), risk of death as a consequence of infection (RR = 1.60; CI 95% = 0.68 to 3.75, *P *= 0.279) and febrile neutropenia (RR = 1.14; CI 95% = 0.80 to 1.63; *P *= 0.478). In contrast, the pooled RRs for risk of leucopenia (RR = 1.24; CI 95% = 1.12 to 1.37; *P *< 0.001), granulocytopenia (RR = 1.07; CI 95% 1.02 to 1.12; *P *= 0.008) and overall response (1.12, 95% CI 1.09 to 1.15 *P *< 0.001) indicated that patients receiving R-C had a greater risk of toxic effects, but a better end of treatment outcome, than patients in C arm.

Heterogeneity was negligible (that is, <20.0%) in meta-analyses of risk of infection, risk of death as a consequence of infection and febrile neutropenia; moderate (that is, 20.0% to 49.9%) in meta-analyses of leucopenia and granulocytopenia; and strong (that is, >50%) in the meta-analysis of overall response.

### Outcomes not included in the meta-analyses

Patient-based risk of herpes zoster infections was reported in one study only (Coiffer 2002) that found 9/202 and 2/197 cases, in R-C and C arms respectively. Risk of hepatitis B was investigated in one study (Robak 2010) which reported 5/272 cases in R-C and 0/272 in the C arm (this study included only patients without chronic hepatitis B). Cases of pneumonia were reported in one study (Robak 2010), which found 15/272 and 16/272 for R-C and C respectively.

One study (Aviles 2010) gave a detailed report of infections in the R-C and C arms measured as the number of cycles in which at least one event occurred. This included: febrile neutropenia (8/282 and 12/318), sepsis (19/282 and 16/318), pneumonia (71/282 and 49/318), urinary infection (12/282 and 8/318), cytomegalovirus infection (28/282 and 3/318), herpes zoster (16/282 and 0/318), respiratory syncytial virus (7/282 and 0/318) and parvovirus (1/282 and 0/318).

Grade 3 and 4 lymphopenia was reported in one study (Fosterprint 2004) occurring in 51.2% and 39.4% of the R-C and C arms respectively (the precise number of cycles in either arm was not given).

### Risk of bias across the studies

**Figure 3:**
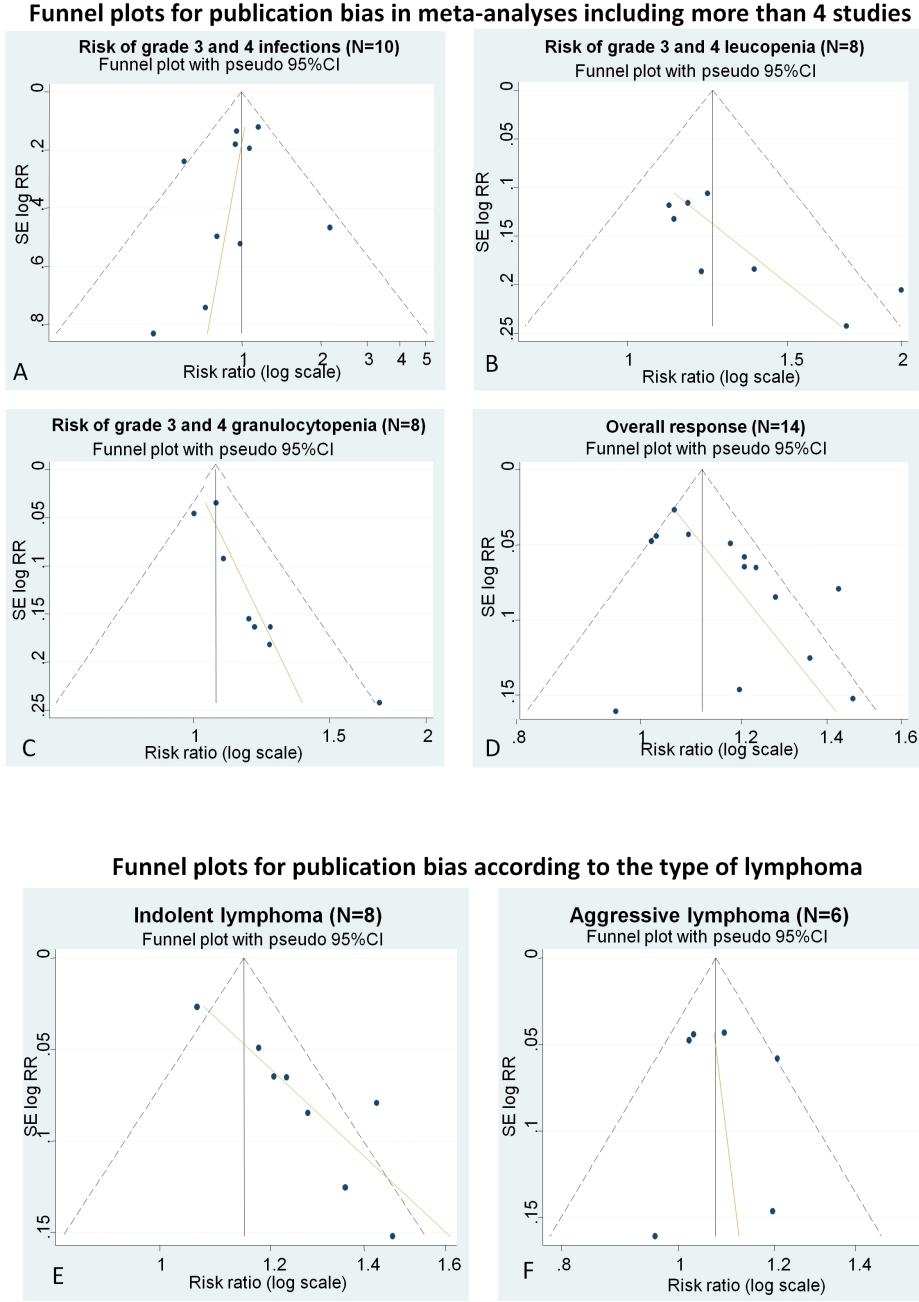
**Funnel plots**. This figure shows funnel plots produced using data from different meta-analyses including more than four studies. X axes report risk ratio (RR) in the log scale; Y axes report the standard error of natural logarithm of risk ratio (SE log(RR)). **T**he Egger's line (in orange) shows the degree of asymmetry. Relevant asymmetry, assessable by the degree of deviation of Egger's line indicates a high risk of potential publication bias. Asymmetry was negligible for risk of infection **(A) **and significant for risk of leucopenia **(B) **and risk of granulocytopenia **(C)**. Asymmetry was also significant for overall response **(D)**; however, funnel plots produced using data dividing studies according to the type of lymphoma (aggressive/indolent) showed persistently strong heterogeneity for indolent lymphomas **(E) **but negligible asymmetry for aggressive lymphomas **(F)**.

Figure 3 shows the effect of publication bias. Overall the analysis showed that smaller studies tended to report higher overall response and lower risk of infections. Funnel plot asymmetry was mild for risk of infections (Figure 3A), suggesting low risk of publication bias, and substantial for leucopenia, (Figure 3B), granulocytopenia (Figure 3C) and overall response (Figure 3D), suggesting a high risk of publication bias. However, since type of ML is one of the major predictors of response to therapy we performed an additional analysis to evaluate plot asymmetry for overall response either in indolent (Figure 3E) or aggressive (Figure 3F) lymphomas. In this way we found that asymmetry was greater for indolent lymphomas than for the aggressive lymphomas group.

Table 3 shows the results of the assessment of quality of evidence supporting each outcome according to the GRADE approach. The analysis indicates moderate to very low quality.

**Table 3:** Results of the assessment of quality of evidence

Assessment of body of evidence supporting each outcome meta-analysis						
**Outcomes**		**Within study risk of bias**	**Imprecision**	**Indirectness of evidence**	**Funnel plot asymmetry** **(Risk of pub bias)**	**Judgment**

Infections		present	not present	not present	not present	Moderate
Death for infection		present	present	not present	not assessed	Very low
Febrile neutropenia		present	present	not present	not assessed	Very low
Leucopenia		present	not present	not present	present	Low
Granulocytopenia		present	not present	not present	present	Low
Overall response	*Overall*	present	not present	not present	present	Low
	*Indolent*	present	not present	not present	present	Low
	*Aggressive*	present	not present	not present	not present	Moderate

### Additional analysis

**Figure 4:**
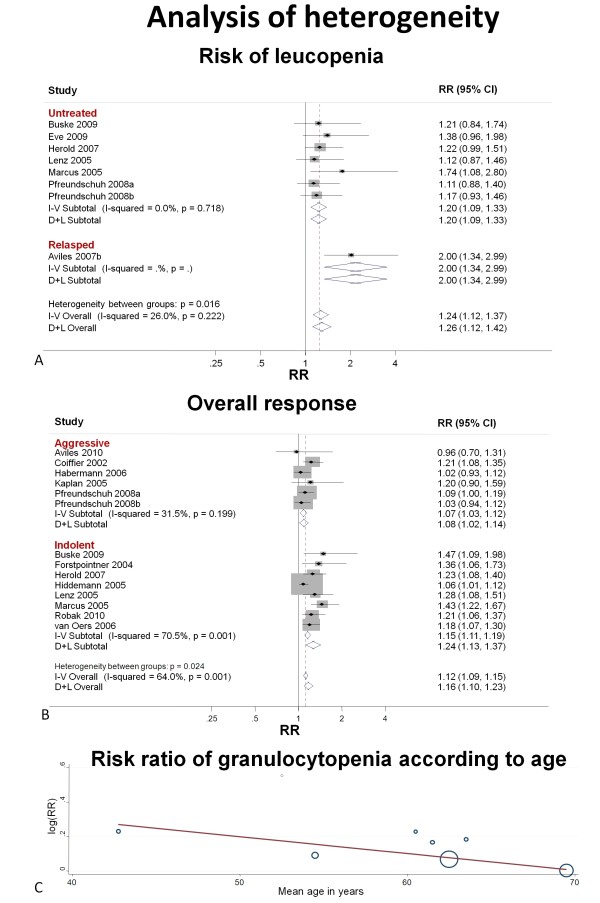
**Analysis of heterogeneity**. This figure shows the analysis of heterogeneity. **A) **Subgroup analysis of risk of grades 3 and 4 leucopenia according to the line of treatment, all heterogeneity found in the overall analysis was explained by between-group heterogeneity. This analysis indicates that previously untreated patients had a lower (although present) increased risk of developing severe leucopenia after R-C than refractory patients. **B) **Subgroup analysis of overall response according to he type of lymphoma, moderate and strong heterogeneity persisted after subgroup analysis in aggressive and indolent lymphomas subgroup respectively. In particular, very little difference is present between random-effect and fixed-effect model RR estimates in the aggressive subgroup while in the indolent subgroup, fixed-effect and random-effect models diverge more consistently. **C) **A simple meta-regression model to analyse risk of grade 3 and 4 granulocytopenia according to age is shown. The diameter of each circle is the inverse of its within-study variance. The graph shows that risk of granulocytopenia is inversely related to age, this may be due to the differential use of granulocyte stimulating factors between older and younger adults. RR = risk ratio, 95% CI = 95% confidence interval, I-V = inverse variance estimates; D+L = DerSimonian and Laird estimates.

Subgroup analysis according to the line of treatment (Figure 4A) explained the heterogeneity found in the meta-analysis for leucopenia. This analysis shows that the previously untreated subgroup had a homogenous and lower pooled RR (RR 1.20, 95% CI 1.09 to 1.33, I-squared 0%) than the refractory subgroup (RR 2.00 95% CI 1.34 to 2.99, one study only). A partial explanation of heterogeneity in the overall response was given by subgroup analysis according to the type of ML (Figure 4B). In the aggressive lymphomas subgroup we found a moderate heterogeneity within-group (I-squared 31.5%, *P *= 0.199), with minimal difference between fixed-effect and random-effect model estimates, while in the indolent lymphomas subgroup the heterogeneity remained strong (I-squared 70.5%, *P *= 0.001). Nevertheless, a beneficial effect was estimated by both fixed-effect and random-effect models in the aggressive lymphomas subgroup (RR 1.07, 95% CI 1.03 to 1.12 and RR 1.08 95% CI 1.02 to 1.14 in fixed-effect and random-effect respectively) and indolent lymphomas subgroup (RR 1.15 95% CI 1.11 to 1.19 and RR 1.24 95% CI 1.13 to 1.37 in fixed-effect and random-effect respectively).

Results of the simple meta-regression model are provided in Table 4. Figure 4C shows the effect of mean age of participants on RR of granulocytopenia. The model indicates that RR of granulocytopenia is estimated to be multiplied by 0.99 (0.98 to 1.00, *P *= 0.080) for each additional year of increase of the median age of study population (that is, the RR granulocytopenia of R-C vs. C is estimated to be multiplied by a factor 0.99^10 ^= 0.90 for each 10 years of age increase of the study population). This model could explain all the heterogeneity found in the meta-analysis (residual heterogeneity 0%). In addition since mean age seemed to explain the heterogeneity also for leucopenia (I-squared = 0%, *P *= 0.237) which was already explained by line of treatment in subgroup analysis, we analysed the effect of mean age and line of treatment as concomitant covariates (using a bivariate meta-regression model). The results of this model are reported in Table 5 and show that line of treatment remained the best predictor of heterogeneity for severe leucopenia.

**Table 4:** The simple meta-regression analysis to explore heterogeneity for overall response, leucopenia and granulocytopenia according to continuous variables.

Meta-regression model									
**Studies characteristics**	**Leucopenia**			**Granulocytopenia **			**Overall response**		
	
	**Res**.	**Coeff. (95% CI)**	**p**	**Res**.	**Coeff. (95% CI)**	**p**	**Res**.	**Coeff. (95% CI)**	**p**

Mean age	**0%**	0.94 (0.67 to 1.30)	**0.237**	**0%**	**0.99** **(0.97 to 1.00)**	**0.080**	64.7%	0.99 (0.98 to 1.00)	0.271
Number of R cycles	32.9%	0.95 (0.79 to 1.15)	0.561	13.2%	1.03 (0.97 to 1.08)	0.255	66.5%	1.01 (0.97 to 1.05)	0.645

The analysis indicates that mean age may explain heterogeneity found for granulocytopenia and leucopenia. Coeff. = regression coefficient in exponential form; Res. = residual heterogeneity. CI = confidence interval

**Table 5:** The bivariate metaregression analysis to explore heterogeneity for leucopenia according mean age and line of treatment simultaneously.

Bivariate meta-regression model			
**Studies characteristics**	**Leucopenia**		
	
	**Res.**	**Coeff. (CI 95%)**	***P***

Mean age	0%	0.99 (0.96 to 1.01)	0.279
Previous treatment		**1.59** **(0.91 to 2.78)**	**0.084**

The analysis confirms the results of subgroup analysis which indicates that the line of treatment is the best predictor of heterogeneity for severe leucopenia. Coeff. = regression coefficient in exponential form; Res. = residual heterogeneity

Meta-regression according to the minimum or number of R cycles did not provide additional evidence to explain heterogeneity (Table 4) and a similar result was obtained for the maximum or number of R cycles (data not shown)

Sensitivity analyses were conducted for all considered outcomes. These did not show any substantial difference between the pooled RRs in the overall meta-analyses and the meta-analyses of selected studies (complete results on sensitivity analysis, indicating only slight changes in leucopenia and granulocytopenia estimates, are reported in Additional file 9).

Table 6 shows the summary of evidence.

**Table 6:** The overall results of the study.

Summary of evidence							
**Outcomes**		**Risk ratio** **(95% CI)**	***P***	**Num. studies**	**Num. of participants**	**Quality of evidence**	**Heterogeneity (H)**

**Infections**		1.00 (0.87 to 1.14)	0.943	10	3,585	Moderate	I^2^<20% not further investigated
**Death from infection**		1.60 (0.68 to 3.75)	0.279	3	1,161	Very Low	I^2^<20% not further investigated
**Febrile neutropenia**		1.14 (0.80 to 1.63)	0.496	2	702	Very Low	None
**Leucopenia**	*Overall*	1.24 (1.12 to 1.37)	>0.001	8	2287	Low	No within group H after subgroup analysis for line of treatment, between group H *P *= 0.016
	*Untreated*	1.20 (1.09 to 1.33)	>0.001	7	2,091		
	*Refractory*	2.00 (1.34 to 2.99)	0.001	1	196		
**Granulocytopenia**		1.07 (1.02 to 1.12)	0.008	8	2,381	Low	H disappears in metaregression according to mean age
**Overall response**	*Overall*	1.12 (1.09 to 1.15)	>0.001	14	4,703	Low	Persistent strong H in indolent lymphomas and moderate H in aggressive lymphomas after subgroup and metaregression analyses.
	*Indolent lymphomas*	1.15 (1.11 to 1.19)	>0.001	6	2,417	Low	
	*Aggressive lymphomas*	1.07 (1.03 to 1.12)	0.002	8	2,286	Moderate	

CI = 95% confidence interval; H = heterogeneity; 95% Num. = number

## Discussion

This is the largest systematic review of infections in patients (5,259) randomised to receive C with or without R as part of induction protocol for CD20+ ML. Several relevant results emerged: 1). addition of R to standard C does not increase the global risk of infections nor does it increase the risk of lethal infections during therapy in patients with CD20+ ML; 2). there is a paucity of data about reactivation of latent viral pathogens which might be relevant in particular groups of patients (for example: HIV positive subjects and HBV carriers); 3). the addition of R to standard C can increase the risk of severe leucopenia and granulocytopenia during therapy in patients with CD20+ ML; and 4). compared to standard chemotherapy alone, R-C increases the overall response both in indolent and aggressive lymphomas clinical variants.

Of the 10 studies included in the meta-analysis for global risk of infection, none found a significant increase of infections in the R-C arm compared to the standard C arm, and one (Coiffer 2002) found a reduction (RR = 0.60, 95% CI 0.38 to 0.96). The evidence for no increased risk is strong and is supported by low risk of publication bias, negligible heterogeneity in the meta-analysis and is consistent with indications from other systematic reviews [4,5]. However, the results may suffer from potential bias due to sub-optimal quality of the studies. In particular, the absence of proper allocation concealment in all the included studies is a relevant issue. Indeed, a proper blinding is not always achievable when frail subjects are to undergo parenteral drug therapies, as this would involve using an inactive IV infusion compound which may potentially expose patients in the control arm to additional (and perhaps, unjustified) risks. Although justified by ethical and practical reasons, the absence of proper blinding may bias results in two different ways. On one hand, the results may underestimate the actual RR. In fact, the awareness of patients' allocation may lead to systematically different and/or wider use of adjuvant treatments in the R-C arms (for example, use of anti-infective prophylaxis or G-CSF) than in the control arm. On the other hand, the results may overestimate the actual effect of R on infection, as the consequence of the fact that patients in the intervention arm are more frequently exposed to IV procedures, such as R administrations.

The issue of whether or not the addition of R to standard C may facilitate the reactivation of latent pathogens in selected groups of patients (for example, HIV positive subjects and HBV carriers) is more difficult to examine for at least three reasons. Only one RCT (Kaplan 2005) included HIV positive patients and, although infections were accurately reported, we could not use them in the meta-analysis since disaggregate data of infections during therapy and during follow-up were not provided (it is noteworthy that this study found a statistically significant increase of risk of infection in the R-C arm mainly due to patients with pre-treatment CD4 counts less than 50 cells/ml). Second, only a few studies reported incident infections by aetiology. In particular, only one study systematically reported all infections by diagnosis (Aviles 2010) and three studies reported viral reactivation (Aviles 2010 and Coiffer 2002 herpetic infections; Robak 2010 HBV). These studies suggest that viral reactivations are more frequent in the R-C arm which is consistent with data from a recent review including non-comparative studies [40], but we could not perform any specific analysis due to paucity of data. Third, RCTs selected only patients who were at very low or no risk of reactivation of relevant viral pathogens such as HBV.

The addition of R to standard C is associated with increased risk of severe leucopenia and granulocytopenia. Leucopenia and granulocytopenia were both reported in eight studies, both had a suggestion of publication bias and both showed moderate heterogeneity in overall meta-analysis that was eventually explained by subgroup or meta-regression analysis. In particular, heterogeneity for leucopenia was due to one study (Aviles 2007b), which included refractory patients only. The subgroup analysis according to the line of treatment showed no within-group heterogeneity and a strong between-groups heterogeneity (*P *= 0.016). This effect may be explained by the more likely occurrence of leucopenia in frail patients undergoing aggressive therapy for refractory disease than in patients receiving first line treatment. The problem of heterogeneity for granulocytopenia is more complicated and this heterogeneity disappeared in meta-regression analysis according to the mean age of study population. The meta-regression indicates an inverse relation between mean age and RR (exponential regression coefficient 0.99, 95% CI 0.98 to 1.00, *P *= 0.080). This result is at first sight anomalous since granulocytopenia might be expected to become more common with increasing age. However, we believe this could be explained by a greater use of G-CSF in RCTs enrolling older patients. G-CSF was mainly given according to clinical judgment, so it is possible that clinicians were more likely to give G-CSF to older patients. Published studies suggest that the use of G-CSF is beneficial in elderly patients with cancer [41] and in those receiving R-containing therapies [42].

Finally, the results of meta-analysis on efficacy were consistent with other reviews focused on this issue [4,5] which have shown that R-C increases the response to therapy in patients with ML and that this effect is more evident in indolent than in aggressive lymphoma.

Several limitations may affect the result of the present study. First, the pooled RR for overall response in indolent ML may be overestimated as a result of publication bias. In fact, smaller studies suggest a more beneficial effect of R than larger ones (asymmetry of funnel plot) and small negative studies are less likely to be published than large negative ones. Therefore, we may have missed a number of small negative studies. Second, real associations between R-C and countable outcomes (that is, infections, leucopenia, granulocytopenia and febrile neutropenia) may be misinterpreted. In fact, we could not obtain additional data from authors about the rates of these events, and, basing these meta-analyses on published data only, we used the risk (number of patients with at least one event over overall exposed at risk) as the measure of association. The degree and direction of this potential bias is not predictable. Third, the choice of dividing all ML into only aggressive and indolent clinical type (due to a lack of disaggregated outcomes data by histology of different indolent ML) may be too simplistic and it may explain the strong heterogeneity found in the overall response meta-analysis of indolent clinical type. Fourth, we considered only grade 3 and 4 infections, which occurred during induction therapy; therefore, we did not estimate the potential risk of late onset infections, the potential increased risk of infection in patients who eventually undergo maintenance therapy and the actual risk of mild and moderate infection (it is noteworthy that there is evidence of increased risk of infections when long lasting R-based regimens, such as maintenance protocols, are undertaken [10,43]). Fifth, the quality of all the meta-analyses might be affected by the sub-optimal quality of the studies. In particular, none of the studies was blinded and patients underwent a number of unrestricted adjuvant therapies such as use of anti-infective agents and G-CSF. Sixth, due to lack of published data, we could not definitively determine whether or not the addition of R to standard C may facilitate the reactivation of latent pathogens. Finally, we did not include two papers written in Chinese. We could not retrieve the authors' e-mail addresses nor were we able to obtain the full-text copy of the papers in the original language to be translated. Although such as exclusion might potentially bias our results, we do not believe this will affect the general application of our findings.

## Conclusions

In conclusion we find that R-C is superior to standard C in terms of overall response in patients affected from CD20 positive ML without increasing the incidence of severe infections. In addition, overall survival has been improved in many of the included studies as also reported in recent systematic reviews focused on R efficacy in induction treatment of CD20+ ML [4-6]. Nevertheless, data on patients with overt and/or latent viral infections are lacking and in our opinion, more studies are needed to explore the potential effect of R on latent viral infections (for example, herpesvirus and HBV infections) and on selected groups of patients (for example, patients with advanced HIV infection, anti-HBc positive and/or HBsAg positive patients). Future studies should preferably be RCTs or, if RCTs are considered inappropriate, they should be large and methodologically sound observational ones (such as prospective cohort studies). To increase the quality of future systematic reviews, new studies should provide the overall number of countable outcomes along with the time at risk.

## Abbreviations

95% CI: 95% confidence interval; Anti-HBc: anti hepatitis B virus core antigen antibodies; Anti-HBs: anti hepatitis B virus surface antigen antibodies; CEOP: cyclophosphamide, epirubicin, vincristine and prednisone (each cycle given every 21 days); CHOP 14: cyclophosphamide, doxorubicin, vincristine and prednisone (each cycle given every 14 days); CHOP 21: cyclophosphamide, doxorubicin, vincristine and prednisone (each cycle given every 21 days); C: chemotherapy; CLL: chronic lymphocytic leukemia/small lymphocytic lymphoma; CVP: cyclophosphamide, vincristine and prednisone (each cycle given every 14 days); DLBCL: diffuse large B cell lymphoma; ESHAP: etoposide, methylprednisolone, cytarabine and cisplatin (each cycle every 28 days); FC: cyclophosphamide and fludarabine (each cycle given every 28 days); FCM: cyclophosphamide, fludarabine and mitoxantrone (each cycle given every 28 days); FL: follicular lymphoma; G-CSF: granulocyte colony stimulating factor; GRADE: Grades of Recommendation, Assessment, Development and Evaluation Working Group; HBsAg: hepatitis B virus surface antigen; HBV: hepatitis B virus; HIV: human immunodeficiency virus; IV: intra-venous; LPL: lymphoplasmacytic lymphoma; MCP: mitoxantrone, chlorambucil and prednisone (each cycle given every 28 days); ML: malignant lymphoma; MoAb: monoclonal antibody; MZL: marginal zone lymphoma; NCI: US National Cancer Institute; *P*: *P*-value; PRISMA: Preferred reporting items for systematic reviews and meta-analyses; R: rituximab; R-C: chemotherapy plus rituximab; RCT: randomized controlled trial; RR: risk ratio.

## Competing interests

The authors declare that they have no competing interests.

## Authors' contributions

SL designed the study, set up the Epi-Info databases, defined the search strings, performed the literature search, selected papers, assessed papers, extracted data, made the analysis, interpreted results and drafted the text. ACM performed the literature search, selected papers, assessed papers, extracted data, interpreted results, and reviewed and approved the final text. PEF, AGP and GI interpreted results, and reviewed and approved the final text. CK approved the study design, interpreted results, reviewed the text and gave final approval to the paper.

## Pre-publication history

The pre-publication history for this paper can be accessed here:

http://www.biomedcentral.com/1741-7015/9/36/prepub

## Supplementary Material

Additional file 1 **PRISMA check list**. PRISMA's items and their application within the paper. Click here for file

Additional file 2 **Strings**. Strings used to perform the search. Click here for file

Additional file 3 **Paper selection**. Summary of paper selection results. Click here for file

Additional file 4 **Included papers**. Bibliography of included papers sorted by author's name. Click here for file

Additional file 5 **Excluded papers**. List of excluded papers with exclusion criteria sorted by year of publication. Click here for file

Additional file 6 **Protocols**. Details of therapies in each different RTC. Click here for file

Additional file 7 **Reported outcome**. Details of studied outcomes as reported in each different RTC. Click here for file

Additional file 8 **Within study risk of bias**. Details of the studies according to within risk of bias of each different RTC. Click here for file

Additional file 9 **Sensitivity analysis**. Summary of complete results of sensitivity analysis. Click here for file
