# Supplementary figures and images for: Risk of infection in patients with lymphoma receiving rituximab: systematic review and meta-analysis
Source: BMC Med. 2011 Apr 12;9:36. doi: 10.1186/1741-7015-9-36 (PMC3094236; doi:10.1186/1741-7015-9-36)

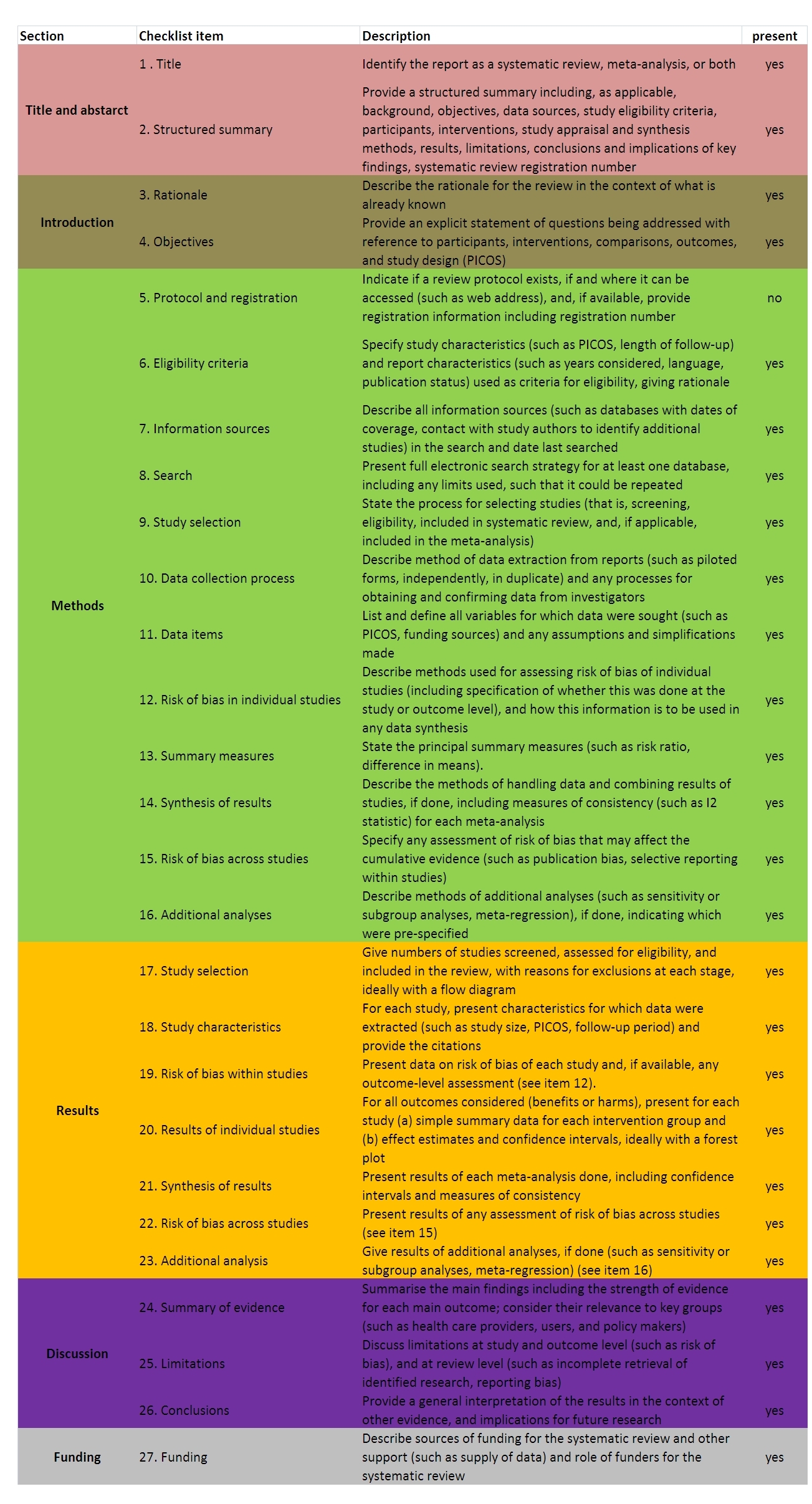

Supplement: Additional file 1 — PRISMA check list. PRISMA's items and their application within the paper. [file 1741-7015-9-36-S1.JPEG]
