# Supplementary material for: Risk of infection in patients with lymphoma receiving rituximab: systematic review and meta-analysis
Source: BMC Med. 2011 Apr 12;9:36. doi: 10.1186/1741-7015-9-36 (PMC3094236; doi:10.1186/1741-7015-9-36)
Supplement: Additional file 2 — Strings. Strings used to perform the search. [file 1741-7015-9-36-S2.RTF]

Appendix 2: search strings


PubMed
("Leukemia"[Mesh] OR "Lymphoma"[Mesh] OR "Neoplasms, Plasma Cell"[Mesh]) AND ("rituximab "[Substance Name] OR "R-FCM protocol "[Substance Name] OR "rituximab"[All Fields] OR "rituxan"[All Fields] OR "mabthera"[all fields] OR "IDEC-C2B8"[all Fields]) AND ("humans"[MeSH Terms] AND "adult"[MeSH Terms])
CENTRAL
1.	rituximab
2.	mabthera
3.	rituxan
4.	idec*
5.	#1 or #2 # or #3 or #4
6.	MeSH descriptor Lymphoma explode all trees
7.	MeSH descriptor Leukemia explode all trees
8.	MeSH descriptor Neoplasms, Plasma Cell explode all trees
9.	#6 or #7 or #8
10.	#5 and #9
11.	11.MeSH descriptor Adult explode all trees
12.	#10 and #11


Embase
1.	exp leukemia/ 
2.	exp Waldenstroem macroglobulinemia/    
3.	exp plasmacytoma/    
4.	exp B cell lymphoma/    
5.	1 or 2 or 3 or 4    
6.	exp rituximab/    
7.	rituxan.mp. [mp=title, abstract, subject headings, heading word, drug trade name, original title, device manufacturer, drug manufacturer]    
8.	mabthera.mp. [mp=title, abstract, subject headings, heading word, drug trade name, original title, device manufacturer, drug manufacturer]    
9.	rituximab.mp. [mp=title, abstract, subject headings, heading word, drug trade name, original title, device manufacturer, drug manufacturer]    
10.	6 or 7 or 8 or 9    
11.	5 and 10    
12.	(random$ or placebo$).ti,ab.    
13.	((single$ or double$ or triple$ or treble$) and (blind$ or mask$)).ti,ab.    
14.	controlled clinical trial$.ti,ab.    
15.	RETRACTED ARTICLE/    
16.	or/12-15    
17.	(animal$ not human$).sh,hw.    
18.	16 not 17    
19.	18 and 11
