# Supplementary material for: Risk of infection in patients with lymphoma receiving rituximab: systematic review and meta-analysis
Source: BMC Med. 2011 Apr 12;9:36. doi: 10.1186/1741-7015-9-36 (PMC3094236; doi:10.1186/1741-7015-9-36)
Supplement: Additional file 3 — Paper selection. Summary of paper selection results. [file 1741-7015-9-36-S3.RTF]

Appendix 3: results of study selection

Study selection	
	Number			
Overall	729			
Included	16			
Excluded	713			
	Exclusion criterion:	Number	
		No infection outcome 	7	
		Non lymphoma 	7	
		Non identical CHT in either arm 	3	
		Purging, maintenance and sequential protocols	19	
		Non R free arm 	138	
		Non randomized studies	245	
		Other MoAb included 	70	
		Children (aged 16 or less)	1	
		Chinese language	2	
		Abstract/editorial/review/comments 	210	
		Duplicate publication	11*	
Overall 729 papers were evaluated, 16 were included and 713 were excluded. The table shows number of studies excluded by each criterion. 
* This includes papers which escaped electronic de-duplication and other papers which report data already considered in other included studies.
