# Supplementary material for: Risk of infection in patients with lymphoma receiving rituximab: systematic review and meta-analysis
Source: BMC Med. 2011 Apr 12;9:36. doi: 10.1186/1741-7015-9-36 (PMC3094236; doi:10.1186/1741-7015-9-36)
Supplement: Additional file 4 — Included papers. Bibliography of included papers sorted by author's name. [file 1741-7015-9-36-S4.RTF]

Appendix 4: bibliography of included papers (sorted by author's name; N=16).
Avilés A, Neri N, Huerta-Guzmán J, de Jesús Nambo M. ESHAP versus rituximab-ESHAP in frail patients with refractory diffuse large B-cell lymphoma.  Clin Lymphoma Myeloma Leuk. 2010;10(2):125-8. 
Avilés A, Nambo MJ, Neri N, Cleto S, Castañeda C, Huerta-Guzmàn J, Murillo E,  Contreras M, Talavera A, González M. Dose dense (CEOP-14) vs dose dense and rituximab (CEOP-14 +R) in high-risk diffuse large cell lymphoma. Med Oncol. 2007;24(1):85-9.
Avilés A, Nambo MJ, Castañeda C, Cleto S, Neri N, Murillo E, Huerta-Guzmán J,  Contreras M. Rituximab and escalated chemotherapy in elderly patients with aggressive diffuse large-cell lymphoma: a controlled clinical trial. Cancer Biother Radiopharm. 2007;22(2):194-9. 
Buske C, Hoster E, Dreyling M, Eimermacher H, Wandt H, Metzner B, Fuchs R, Bittenbring J, Woermann B, Hohloch K, Hess G, Ludwig WD, Schimke J, Schmitz S, Kneba M, Reiser M, Graeven U, Klapper W, Unterhalt M, Hiddemann W; German Low-Grade Lymphoma Study Group. The addition of rituximab to front-line therapy with CHOP (R-CHOP) results in a higher response rate and longer time to treatment failure in patients with lymphoplasmacytic lymphoma: results of a randomized trial of the German Low-Grade Lymphoma Study Group (GLSG). Leukemia. 2009; 23(1):153-61.
Coiffier B, Lepage E, Briere J, Herbrecht R, Tilly H, Bouabdallah R, Morel P, Van Den Neste E, Salles G, Gaulard P, Reyes F, Lederlin P, Gisselbrecht C. CHOP chemotherapy plus rituximab compared with CHOP alone in elderly patients with diffuse large-B-cell lymphoma. N Engl J Med. 2002;346(4):235-42.
Eve HE, Linch D, Qian W, Ross M, Seymour JF, Smith P, Stevens L, Rule SA. Toxicity of fludarabine and cyclophosphamide with or without rituximab as initial therapy for patients with previously untreated mantle cell lymphoma: results of a randomised phase II study. Leuk Lymphoma. 2009;50(2):211-5.
Forstpointner R, Dreyling M, Repp R, Hermann S, Hänel A, Metzner B, Pott C, Hartmann F, Rothmann F, Rohrberg R, Böck HP, Wandt H, Unterhalt M, Hiddemann W; German Low-Grade Lymphoma Study Group. The addition of rituximab to a combination of fludarabine, cyclophosphamide, mitoxantrone (FCM) significantly increases the  response rate and prolongs survival as compared with FCM alone in patients with relapsed and refractory follicular and mantle cell lymphomas: results of a prospective randomized study of the German Low-Grade Lymphoma Study Group. Blood;104(10):3064-71. 
Habermann TM, Weller EA, Morrison VA, Gascoyne RD, Cassileth PA, Cohn JB, Dakhil SR, Woda B, Fisher RI, Peterson BA, Horning SJ. Rituximab-CHOP versus CHOP alone or with maintenance rituximab in older patients with diffuse large B-cell lymphoma. J Clin Oncol. 2006;24(19):3121-7.
Herold M, Haas A, Srock S, Neser S, Al-Ali KH, Neubauer A, Dölken G, Naumann R, Knauf W, Freund M, Rohrberg R, Höffken K, Franke A, Ittel T, Kettner E, Haak U, Mey U, Klinkenstein C, Assmann M, von Grünhagen U; East German Study Group Hematology and Oncology Study. Rituximab added to first-line mitoxantrone, chlorambucil, and prednisolone chemotherapy followed by interferon maintenance prolongs survival in patients with advanced follicular lymphoma: an East German Study Group Hematology and Oncology Study. J Clin Oncol. 2007;25(15):1986-92. 
Hiddemann W, Kneba M, Dreyling M, Schmitz N, Lengfelder E, Schmits R, Reiser M, Metzner B, Harder H, Hegewisch-Becker S, Fischer T, Kropff M, Reis HE, Freund  M, Wörmann B, Fuchs R, Planker M, Schimke J, Eimermacher H, Trümper L, Aldaoud A, Parwaresch R, Unterhalt M. Frontline therapy with rituximab added to the combination of cyclophosphamide, doxorubicin, vincristine, and prednisone (CHOP)  significantly improves the outcome for patients with advanced-stage follicular lymphoma compared with therapy with CHOP alone: results of a prospective randomized study of the German Low-Grade Lymphoma Study Group. Blood. 2005;106(12):3725-32.
Kaplan LD, Lee JY, Ambinder RF, Sparano JA, Cesarman E, Chadburn A, Levine AM, Scadden DT. Rituximab does not improve clinical outcome in a randomized phase 3 trial of CHOP with or without rituximab in patients with HIV-associated non-Hodgkin lymphoma: AIDS-Malignancies Consortium Trial 010. Blood. 2005;106(5):1538-43.
Lenz G, Dreyling M, Hoster E, Wörmann B, Dührsen U, Metzner B, Eimermacher H, Neubauer A, Wandt H, Steinhauer H, Martin S, Heidemann E, Aldaoud A, Parwaresch R, Hasford J, Unterhalt M, Hiddemann W. Immunochemotherapy with rituximab and cyclophosphamide, doxorubicin, vincristine, and prednisone significantly improves response and time to treatment failure, but not long-term outcome in patients with previously untreated mantle cell lymphoma: results of a prospective randomized trial of the German Low Grade Lymphoma Study Group (GLSG). J Clin Oncol. 2005;23(9):1984-92
Marcus R, Imrie K, Belch A, Cunningham D, Flores E, Catalano J, Solal-Celigny P, Offner F, Walewski J, Raposo J, Jack A, Smith P. CVP chemotherapy plus rituximab compared with CVP as first-line treatment for advanced follicular lymphoma. Blood. 2005;105(4):1417-23.
Pfreundschuh M, Schubert J, Ziepert M, Schmits R, Mohren M, Lengfelder E, Reiser M, Nickenig C, Clemens M, Peter N, Bokemeyer C, Eimermacher H, Ho A, Hoffmann M, Mertelsmann R, Trümper L, Balleisen L, Liersch R, Metzner B, Hartmann F, Glass B, Poeschel V, Schmitz N, Ruebe C, Feller AC, Loeffler M; German High-Grade Non-Hodgkin Lymphoma Study Group (DSHNHL). Six versus eight cycles of  bi-weekly CHOP-14 with or without rituximab in elderly patients with aggressive CD20+ B-cell lymphomas: a randomised controlled trial (RICOVER-60). Lancet Oncol. 2008;9(2):105-16.
Robak T, Dmoszynska A, Solal-Céligny P, Warzocha K, Loscertales J, Catalano J, Afanasiev BV, Larratt L, Geisler CH, Montillo M, Zyuzgin I, Ganly PS, Dartigeas C, Rosta A, Maurer J, Mendila M, Saville MW, Valente N, Wenger MK, Moiseev SI. Rituximab plus fludarabine and cyclophosphamide prolongs progression-free survival compared with fludarabine and cyclophosphamide alone in previously treated chronic lymphocytic leukemia. J Clin Oncol. 2010;28(10):1756-65.
Van Oers MH, Hagenbeek A, Van Glabbeke M, Teodorovic I. Chimeric anti-CD20 monoclonal antibody (Mabthera) in remission induction and maintenance treatment of relapsed follicular non-Hodgkin's lymphoma: a phase III randomized clinicaltrial-Intergroup Collaborative Study. Ann Hematol. 2002;81(10):553-7.
