# Supplementary material for: Risk of infection in patients with lymphoma receiving rituximab: systematic review and meta-analysis
Source: BMC Med. 2011 Apr 12;9:36. doi: 10.1186/1741-7015-9-36 (PMC3094236; doi:10.1186/1741-7015-9-36)
Supplement: Additional file 5 — Excluded papers. List of excluded papers with exclusion criteria sorted by year of publication. [file 1741-7015-9-36-S5.RTF]

Appendix 5: list excluded papers
Paper are sorted by year of publication and exclusion criterion is reported in squared parentheses (N=713).
1.	
1.	Maloney,D. G. et al.Phase I clinical trial using escalating single-dose infusion of chimeric anti-CD20 monoclonal antibody (IDEC-C2B8) in patients with recurrent B-cell lymphoma Blood 1994;84:2457-2466 [Non R free arm]
2.	Maloney,D. G. et al.IDEC-C2B8 (Rituximab) anti-CD20 monoclonal antibody therapy in patients with relapsed low-grade non-Hodgkin's lymphoma Blood 1997;90:2188-2195 [Non randomized studies]
3.	Maloney,D. G. et al.IDEC-C2B8: results of a phase I multiple-dose trial in patients with relapsed non-Hodgkin's lymphoma Journal of clinical oncology  1997;15:3266-3274 [Non R free arm]
4.	Press,O. W. et al.Prospects for the management of non-Hodgkin's lymphomas with monoclonal antibodies and immunoconjugates. Cancer Journal from Scientific American 1998;4:19s-26s [Abstract/editorial/review/comments]
5.	Coiffier,B. et al.Rituximab (anti-CD20 monoclonal antibody) for the treatment of patients with relapsing or refractory aggressive lymphoma: A multicenter phase II study. Blood 1998;92:1927-1932 [Non R free arm]
6.	Tobinai,K. et al.Feasibility and pharmacokinetic study of a chimeric anti-CD20 monoclonal antibody (IDEC-C2B8, rituximab) in relapsed B-cell lymphoma. The IDEC-C2B8 Study Group Annals of Oncology  1998;9:527-534 [Non randomized studies]
7.	McLaughlin,P. et al.Rituximab chimeric anti-CD20 monoclonal antibody therapy for relapsed indolent lymphoma: half of patients respond to a four-dose treatment program Journal of clinical oncology  1998;16:2825-2833 [Non randomized studies]
8.	Sweetenham,J. et al.Cost-minimization analysis of CHOP, fludarabine and rituximab for the treatment of relapsed indolent B-cell non-Hodgkin's lymphoma in the U.K.. British journal of haematology 1999;106:47-54 [Abstract/editorial/review/comments]
9.	Nguyen,D. T. et al.IDEC-C2B8 anti-CD20 (rituximab) immunotherapy in patients with low-grade non-Hodgkin's lymphoma and lymphoproliferative disorders: evaluation of response on 48 patients European journal of haematology 1999;62:76-82 [Non randomized studies]
10.	Byrd,J. C. et al.Rituximab therapy in hematologic malignancy patients with circulating blood tumor cells: association with increased infusion-related side effects and rapid blood tumor clearance Journal of clinical oncology  1999;17:791-795 [Non R free arm]
11.	Piro,L. D. et al.Extended Rituximab (anti-CD20 monoclonal antibody) therapy for relapsed or refractory low-grade or follicular non-Hodgkin's lymphoma Annals of Oncology  1999;10:655-661 [Non randomized studies]
12.	Czuczman,M. S. et al.Treatment of patients with low-grade B-cell lymphoma with the combination of chimeric anti-CD20 monoclonal antibody and CHOP chemotherapy Journal of clinical oncology  1999;17:268-276 [Non randomized studies]
13.	Winkler,U. et al.Cytokine-release syndrome in patients with B-cell chronic lymphocytic leukemia and high lymphocyte counts after treatment with an anti-CD20 monoclonal antibody (rituximab, IDEC-C2B8) Blood 1999;94:2217-2224 [Non R free arm]
14.	McLaughlin,P. et al.Rituximab in indolent lymphoma: the single-agent pivotal trial Seminars in oncology 1999;26:79-87 [Abstract/editorial/review/comments]
15.	Czuczman,M. S. et al.CHOP plus rituximab chemoimmunotherapy of indolent B-cell lymphoma Seminars in oncology 1999;26:88-96 [Abstract/editorial/review/comments]
16.	Buckstein,R. et al.Stem cell function and engraftment is not affected by "in vivo purging" with rituximab for autologous stem cell treatment for patients with low-grade non-Hodgkin's lymphoma Seminars in oncology 1999;26:115-122 [Purging, maintenance and sequential protocols]
17.	Davis,T. A. et al.Single-agent monoclonal antibody efficacy in bulky non-Hodgkin's lymphoma: results of a phase II trial of rituximab Journal of clinical oncology  1999;17:1851-1857 [Non randomized studies]
18.	Witzig,T. E. et al.Phase I/II trial of IDEC-Y2B8 radioimmunotherapy for treatment of relapsed or refractory CD20(+) B-cell non-Hodgkin's lymphoma Journal of clinical oncology  1999;17:3793-3803 [Other MoAb included]
19.	Maloney,D. G. et al.Advances in the immunotherapy of hematologic malignancies: Cellular and humoral approaches. Current opinion in hematology 1999;6:222-228 [Abstract/editorial/review/comments]
20.	Ghielmini,M. et al.The effect of Rituximab on patients with follicular and mantle-cell lymphoma. Swiss Group for Clinical Cancer Research (SAKK) Annals of Oncology 2000;:123-126 [Non randomized studies]
21.	Keating,M. et al.High-dose rituximab therapy in chronic lymphocytic leukemia Seminars in oncology 2000;27:86-90 [Non R free arm]
22.	McLaughlin,P. et al.Safety of fludarabine, mitoxantrone, and dexamethasone combined with rituximab in the treatment of stage IV indolent lymphoma Seminars in oncology 2000;27:37-41 [Non randomized studies]
23.	Foran,J. M. et al.European phase II study of rituximab (chimeric anti-CD20 monoclonal antibody) for patients with newly diagnosed mantle-cell lymphoma and previously treated mantle-cell lymphoma, immunocytoma, and small B-cell lymphocytic lymphoma Journal of clinical oncology  2000;18:317-324 [Non randomized studies]
24.	Weiden,P. L. et al.Pretargeted radioimmunotherapy (PRIT) for treatment of non-Hodgkin's lymphoma (NHL): initial phase I/II study results Cancer biotherapy & radiopharmaceuticals 2000;15:15-29 [Non randomized studies]
25.	Hainsworth,J. D. et al.Rituximab monoclonal antibody as initial systemic therapy for patients with low-grade non-Hodgkin lymphoma Blood 2000;95:3052-3056 [Non randomized studies]
26.	Foran,J. M. et al.A UK multicentre phase II study of rituximab (chimaeric anti-CD20 monoclonal antibody) in patients with follicular lymphoma, with PCR monitoring of molecular response British journal of haematology 2000;109:81-88 [Non randomized studies]
27.	Drapkin,R. et al.Pentostatin and rituximab in the treatment of patients with B-cell malignancies Oncology (Williston Park, N.Y.) 2000;14:25-29 [Non randomized studies]
28.	Davis,T. A. et al.Combination immunotherapy of relapsed or refractory low-grade or follicular non-Hodgkin's lymphoma with rituximab and interferon-alpha-2a Clinical cancer research 2000;6:2644-2652 [Non randomized studies]
29.	Ladetto,M. et al.Rituximab anti-CD20 monoclonal antibody induces marked but transient reductions of peripheral blood lymphocytes in chronic lymphocytic leukaemia patients Medical oncology (Northwood, London, England) 2000;17:203-210 [Non R free arm]
30.	Davis,T. A. et al.Rituximab anti-CD20 monoclonal antibody therapy in non-Hodgkin's lymphoma: safety and efficacy of re-treatment Journal of clinical oncology  2000;18:3135-3143 [Non randomized studies]
31.	Heinzerling,L. M. et al.Reduction of tumor burden and stabilization of disease by systemic therapy with anti-CD20 antibody (rituximab) in patients with primary cutaneous B-cell lymphoma Cancer 2000;89:1835-1844 [Non R free arm]
32.	Feuring-Buske,M. et al.IDEC-C2B8 (Rituximab) anti-CD20 antibody treatment in relapsed advanced-stage follicular lymphomas: results of a phase-II study of the German Low-Grade Lymphoma Study Group Annals of Hematology 2000;79:493-500 [Non randomized studies]
33.	Flinn,I. W. et al.Immunotherapy with rituximab during peripheral blood stem cell transplantation for non-Hodgkin's lymphoma Biology of blood and marrow transplantation  2000;6:628-632 [Non randomized studies]
34.	Coiffier,B. et al.Monoclonal antibodies in the treatment of neoplastic hematologic diseases. Bulletin du cancer 2000;87:839-845 [Abstract/editorial/review/comments]
35.	Gunter Derigs,H. et al.In vitro and in vivo purging of B lymphoma cells from stem-cell products using anti-CD20 Abs. Cytotherapy 2000;2:445-453 [Abstract/editorial/review/comments]
36.	Kyle,R. A. et al.Waldenstrom's macroglobulinaemia: a prospective study comparing daily with intermittent oral chlorambucil British journal of haematology 2000;108:737-742 [Non R free arm]
37.	Bienvenu,J. et al.Tumor necrosis factor alpha release is a major biological event associated with rituximab treatment. Hematology Journal 2001;2:378-384 [Duplicate publication]
38.	Wiseman,G. A. et al.Biodistribution and dosimetry results from a phase III prospectively randomized controlled trial of Zevalin radioimmunotherapy for low-grade, follicular, or transformed B-cell non-Hodgkin's lymphoma Critical reviews in oncology/hematology 2001;39:181-194 [Other MoAb included]
39.	Colombat,P. et al.Rituximab (anti-CD20 monoclonal antibody) as single first-line therapy for patients with follicular lymphoma with a low tumor burden: clinical and molecular evaluation Blood 2001;97:101-106 [Non randomized studies]
40.	Vose,J. M. et al.Phase II study of rituximab in combination with chop chemotherapy in patients with previously untreated, aggressive non-Hodgkin's lymphoma Journal of clinical oncology  2001;19:389-397 [Non randomized studies]
41.	Czuczman,M. S. et al.Clearing of cells bearing the bcl-2 [t(14;18)] translocation from blood and marrow of patients treated with rituximab alone or in combination with CHOP chemotherapy Annals of Oncology 2001;12:109-114 [Non R free arm]
42.	Byrd,J. C. et al.Rituximab using a thrice weekly dosing schedule in B-cell chronic lymphocytic leukemia and small lymphocytic lymphoma demonstrates clinical activity and acceptable toxicity Journal of clinical oncology  2001;19:2153-2164 [Non randomized studies]
43.	O'Brien,S. M. et al.Rituximab dose-escalation trial in chronic lymphocytic leukemia Journal of clinical oncology  2001;19:2165-2170 [Non R free arm]
44.	Aviles,A. et al.Rituximab in the treatment of refractory follicular lymphoma -- six doses are better than four Journal of hematotherapy & stem cell research 2001;10:313-316 [Non R free arm]
45.	Igarashi,T. et al.Re-treatment of relapsed indolent B-cell lymphoma with rituximab International journal of hematology 2001;73:213-221 [Non randomized studies]
46.	Aviles,A. et al.Evaluation on a six-dose treatment of anti CD 20 monoclonal antibody in patients with refractory follicular lymphoma Cancer biotherapy & radiopharmaceuticals 2001;16:159-162 [Non randomized studies]
47.	Joyce,R. M. et al.Rituximab and ifosfamide, mitoxantrone, etoposide (RIME) with Neupogen support for B-cell non-Hodgkin's lymphoma prior to high-dose chemotherapy with autologous haematopoietic transplant European journal of haematology.Supplementum 2001;64:56-62 [Non randomized studies]
48.	Huhn,D. et al.Rituximab therapy of patients with B-cell chronic lymphocytic leukemia Blood 2001;98:1326-1331 [Non randomized studies]
49.	Sacchi,S. et al.Clinical activity and safety of combination immunotherapy with IFN-alpha 2a and Rituximab in patients with relapsed low grade non-Hodgkin's lymphoma Haematologica 2001;86:951-958 [Non randomized studies]
50.	Weiden,P. L. et al.Pretargeted radioimmunotherapy (PRIT) for treatment of non-Hodgkin's lymphoma (NHL) Critical reviews in oncology/hematology 2001;40:37-51 [Other MoAb included]
51.	Tobinai,K. et al.Clinical trials of a mouse-human chimeric anti-CD20 monoclonal antibody (rituximab) for B cell non-Hodgkin's lymphoma in Japan Cancer chemotherapy and pharmacology 2001;:-90 [Non R free arm]
52.	Lauria,F. et al.Efficacy of anti-CD20 monoclonal antibodies (Mabthera) in patients with progressed hairy cell leukemia Haematologica 2001;86:1046-1050 [Non randomized studies]
53.	Mayer,J. et al.Non-cytostatic treatment of malignancies. Anti-CD20 monoclonal antibody (Rituximab, Mabthera) in the treatment of non-Hodgkin's lymphoma Vnitrni lekarstvi 2001;:57-62 [Non randomized studies]
54.	Khouri,I. F. et al.Nonablative allogeneic hematopoietic transplantation as adoptive immunotherapy for indolent lymphoma: low incidence of toxicity, acute graft-versus-host disease, and treatment-related mortality Blood 2001;98:3595-3599 [Non R free arm]
55.	Ladetto,M. et al.Concurrent administration of high-dose chemotherapy and rituximab is a feasible and effective chemo/immunotherapy for patients with high-risk non-Hodgkin's lymphoma Leukemia  2001;15:1941-1949 [Non randomized studies]
56.	Walewski,J. et al.Rituximab (Mabthera, Rituxan) in patients with recurrent indolent lymphoma: evaluation of safety and efficacy in a multicenter study Medical oncology (Northwood, London, England) 2001;18:141-148 [Non randomized studies]
57.	Kanelli,S. et al.Rituximab toxicity in patients with peripheral blood malignant B-cell lymphocytosis Leukemia & lymphoma 2001;42:1329-1337 [Non R free arm]
58.	Grillo-Lopez,A. J. et al.Monoclonal antibodies: A new era in the treatment of non-Hodgkin's lymphoma. Current Pharmaceutical Biotechnology 2001;2:301-311 [Abstract/editorial/review/comments]
59.	Dimopoulos,M. A. et al.Treatment of Waldenstrom's macroglobulinemia with thalidomide Journal of clinical oncology  2001;19:3596-3601 [Non randomized studies]
60.	Burke,J. M. et al.Radioimmunotherapy for acute leukemia. Cancer Control 2002;9:106-113 [Abstract/editorial/review/comments]
61.	Robak,T. et al.Alkylating agents and nucleoside analogues in the treatment of B cell chronic lymphocytic leukemia. Leukemia 2002;16:1015-1027 [Abstract/editorial/review/comments]
62.	Klasa,R. J. et al.Oblimersen Bcl-2 antisense: Facilitating apoptosis in anticancer treatment. Antisense and Nucleic Acid Drug Development 2002;12:193-213 [Abstract/editorial/review/comments]
63.	Grillo-Lopez,A. J. et al.Zevalin: The first radioimmunotherapy approved for the treatment of lymphoma. Expert Review of Anticancer Therapy 2002;2:485-493 [Other MoAb included]
64.	Haanen,J. B. A. G. et al.With mouse more man. Effective treatment with monoclonal antibodies. Pharmaceutisch weekblad 2002;137:1724-1729 [Abstract/editorial/review/comments]
65.	Coiffier,B. et al.Aggressive lymphoma: Improving treatment outcome with rituximab. Anti-Cancer Drugs 2002;13:43s-50s [Abstract/editorial/review/comments]
66.	Tallman,M. S. et al.Current treatment strategies for patients with hairy cell leukemia. Reviews in Clinical and Experimental Hematology 2002;6:389-400 [Abstract/editorial/review/comments]
67.	Sarris,A. H. et al.Quantitative real-time polymerase chain reaction for monitoring minimal residual disease in patients with advanced indolent lymphomas treated with rituximab, fludarabine, mitoxantrone, and dexamethasone Seminars in oncology 2002;29:48-55 [Non randomized studies]
68.	Behr,T. M. et al.High-dose myeloablative radioimmunotherapy of mantle cell non-Hodgkin lymphoma with the iodine-131-labeled chimeric anti-CD20 antibody C2B8 and autologous stem cell support. Results of a pilot study Cancer 2002;94:1363-1372 [Other MoAb included]
69.	Witzig,T. E. et al.Randomized controlled trial of yttrium-90-labeled ibritumomab tiuxetan radioimmunotherapy versus rituximab immunotherapy for patients with relapsed or refractory low-grade, follicular, or transformed B-cell non-Hodgkin's lymphoma Journal of clinical oncology  2002;20:2453-2463 [Other MoAb included]
70.	Portlock,C. S. et al.CHOP and rituximab in elderly patients Current oncology reports 2002;4:414- [Duplicate publication]
71.	Forstpointner,R. et al.Increased response rate with rituximab in relapsed and refractory follicular and mantle cell lymphomas -- results of a prospective randomized study of the German Low-Grade Lymphoma Study Group Deutsche medizinische Wochenschrift (1946) 2002;127:2253-2258 [Duplicate publication]
72.	Anonymous et al.Rituxan delays disease progression in indolent non-Hodgkin's lymphoma Oncology (Williston Park, N.Y.) 2002;16:1472-1475 [Purging, maintenance and sequential protocols]
73.	Carswell,C. I. et al.90Y ibritumomab tiuxetan. American Journal of Cancer 2002;1:341-348 [Other MoAb included]
74.	Ansell,S. M. et al.Phase 1 study of interleukin-12 in combination with rituximab in patients with B-cell non-Hodgkin lymphoma Blood 2002;99:67-74 [Non randomized studies]
75.	Tirelli,U. et al.Infusional CDE with rituximab for the treatment of human immunodeficiency virus-associated non-Hodgkin's lymphoma: preliminary results of a phase I/II study Recent results in cancer research 2002;159:149-153 [Non randomized studies]
76.	Rambaldi,A. et al.Monitoring of minimal residual disease after CHOP and rituximab in previously untreated patients with follicular lymphoma Blood 2002;99:856-862 [Non R free arm]
77.	Hainsworth,J. D. et al.Rituximab as first-line and maintenance therapy for patients with indolent non-Hodgkin's lymphoma: interim follow-up of a multicenter phase II trial Seminars in oncology 2002;29:25-29 [Non randomized studies]
78.	Wilson,W. H. et al.The role of rituximab and chemotherapy in aggressive B-cell lymphoma: a preliminary report of dose-adjusted EPOCH-R Seminars in oncology 2002;29:41-47 [Non randomized studies]
79.	Mangel,J. et al.Immunotherapy with rituximab following high-dose therapy and autologous stem-cell transplantation for mantle cell lymphoma Seminars in oncology 2002;29:56-69 [Non randomized studies]
80.	Howard,O. M. et al.Rituximab and CHOP induction therapy for newly diagnosed mantle-cell lymphoma: molecular complete responses are not predictive of progression-free survival Journal of clinical oncology  2002;20:1288-1294 [Non randomized studies]
81.	Wiseman,G. A. et al.Radiation dosimetry results for Zevalin radioimmunotherapy of rituximab-refractory non-Hodgkin lymphoma Cancer 2002;94:1349-1357 [Non R free arm]
82.	Berkahn,L. et al.In vivo purging with rituximab prior to collection of stem cells for autologous transplantation in chronic lymphocytic leukemia Journal of hematotherapy & stem cell research 2002;11:315-320 [Non randomized studies]
83.	Weide,R. et al.Bendamustine mitoxantrone and rituximab (BMR): a new effective regimen for refractory or relapsed indolent lymphomas Leukemia & lymphoma 2002;43:327-331 [Non randomized studies]
84.	Wiseman,G. A. et al.Ibritumomab tiuxetan radioimmunotherapy for patients with relapsed or refractory non-Hodgkin lymphoma and mild thrombocytopenia: a phase II multicenter trial Blood 2002;99:4336-4342 [Other MoAb included]
85.	Flohr,T. et al.Rituximab in vivo purging is safe and effective in combination with CD34-positive selected autologous stem cell transplantation for salvage therapy in B-NHL Bone marrow transplantation 2002;29:769-775 [Non randomized studies]
86.	Rohatgi,N. et al.Phase II trial of sequential therapy with fludarabine followed by cyclophosphamide, mitoxantrone, vincristine, and prednisone for low-grade follicular lymphomas American Journal of Hematology 2002;70:181-185 [Non R free arm]
87.	Igarashi,T. et al.Factors affecting toxicity, response and progression-free survival in relapsed patients with indolent B-cell lymphoma and mantle cell lymphoma treated with rituximab: a Japanese phase II study Annals of Oncology  2002;13:928-943 [Abstract/editorial/review/comments]
88.	Witzig,T. E. et al.Treatment with ibritumomab tiuxetan radioimmunotherapy in patients with rituximab-refractory follicular non-Hodgkin's lymphoma Journal of clinical oncology  2002;20:3262-3269 [Other MoAb included]
89.	Jaeger,G. et al.Rituximab (anti-CD20 monoclonal antibody) as consolidation of first-line CHOP chemotherapy in patients with follicular lymphoma: a phase II study European journal of haematology 2002;69:21-26 [Non randomized studies]
90.	Scheidhauer,K. et al.Biodistribution and kinetics of (131)I-labelled anti-CD20 MAB IDEC-C2B8 (rituximab) in relapsed non-Hodgkin's lymphoma European journal of nuclear medicine and molecular imaging 2002;29:1276-1282 [Other MoAb included]
91.	Hainsworth,J. D. et al.Rituximab as first-line and maintenance therapy for patients with indolent non-hodgkin's lymphoma Journal of clinical oncology  2002;20:4261-4267 [Non randomized studies]
92.	Schulz,H. et al.Phase 2 study of a combined immunochemotherapy using rituximab and fludarabine in patients with chronic lymphocytic leukemia Blood 2002;100:3115-3120 [Non randomized studies]
93.	Domingo-Domenech,E. et al.Combined treatment with anti-CD20 (rituximab) and CHOP in relapsed advanced-stage follicular lymphomas Haematologica 2002;87:1229-1230 [Non randomized studies]
94.	Gibson,A. D. et al.Updated results of a Phase III trial comparing ibritumomab tiuxetan with rituximab in previously treated patients with non-Hodgkin's lymphoma Clinical lymphoma 2002;3:87-89 [Other MoAb included]
95.	Emmanouilides,C. et al.Mitoxantrone/ifosfamide/etoposide salvage regimen with rituximab for in vivo purging in patients with relapsed lymphoma Clinical lymphoma 2002;3:111-116 [Non randomized studies]
96.	Patte,C. et al.Treatment of mature B-ALL and high grade B-NHL in children. Best Practice and Research in Clinical Haematology 2002;15:695-711 [Abstract/editorial/review/comments]
97.	Treon,S. P. et al.CD20-directed serotherapy in patients with multiple myeloma: biologic considerations and therapeutic applications Journal of immunotherapy (Hagerstown, Md. 2002;25:72-81 [Non randomized studies]
98.	Dimopoulos,M. A. et al.Extended rituximab therapy for previously untreated patients with Waldenstrom's macroglobulinemia Clinical lymphoma 2002;3:163-166 [Non randomized studies]
99.	Dimopoulos,M. A. et al.Treatment of Waldenstrom's macroglobulinemia with rituximab Journal of clinical oncology  2002;20:2327-2333 [Non randomized studies]
100.	Coiffier,B. et al.Monoclonal antibodies combined to chemotherapy for the treatment of patients with lymphoma. Blood reviews 2003;17:25-31 [Abstract/editorial/review/comments]
101.	Plosker,G. L. et al.Rituximab: A review of its use in non-Hodgkin's lymphoma and chronic lymphocytic leukaemia. Drugs 2003;63:803-843 [Abstract/editorial/review/comments]
102.	Frankel,S. R. et al.Oblimersen sodium (G3139 Bcl-2 antisense oligonucleotide) therapy in Waldenstrom's macroglobulinemia: A targeted approach to enhance apoptosis. Seminars in oncology 2003;30:300-304 [Abstract/editorial/review/comments]
103.	Ioannidis,J. P. A. et al.Levels of absolute survival benefit for systemic therapies of advanced cancer: A call for standards. European journal of cancer 2003;39:1194-1198 [Abstract/editorial/review/comments]
104.	Ansell,S. M. et al.Adding cytokines to monoclonal antibody therapy: Does the concurrent administration of interleukin-12 add to the efficacy of rituximab in B-cell non-Hodgkin lymphoma?. Leukemia and Lymphoma 2003;44:1309-1315 [Abstract/editorial/review/comments]
105.	Byrd,J. C. et al.Randomized phase 2 study of fludarabine with concurrent versus sequential treatment with rituximab in symptomatic, untreated patients with B-cell chronic lymphocytic leukemia: results from Cancer and Leukemia Group B 9712 (CALGB 9712) Blood 2003;101:6-14 [Purging, maintenance and sequential protocols]
106.	Herold,M. et al.Randomized phase III study for the treatment of advanced indolent non-Hodgkin's lymphomas (NHL) and mantle cell lymphoma: chemotherapy versus chemotherapy plus rituximab Annals of Hematology 2003;82:77-79 [Duplicate publication]
107.	Timmerman,J. M. et al.Immunotherapy for lymphomas. International journal of hematology 2003;77:444-455 [Abstract/editorial/review/comments]
108.	Korolenko,V. O. et al.Clinical trial of the monoclonal antibody drug mabthera (Rituximab) in the treatment of non-Hodgkin lymphoma Voprosy onkologii 2003;49:459-463 [Non R free arm]
109.	Wiseman,G. A. et al.Additional radiation absorbed dose estimates for Zevalin radioimmunotherapy Cancer biotherapy & radiopharmaceuticals 2003;18:253-258 [Non R free arm]
110.	Galimberti,S. et al.Quantitative molecular evaluation in autotransplant programs for follicular lymphoma: efficacy of in vivo purging by Rituximab Bone marrow transplantation 2003;32:57-63 [Purging, maintenance and sequential protocols]
111.	Mounier,N. et al.Rituximab plus CHOP (R-CHOP) overcomes bcl-2-associated resistance to chemotherapy in elderly patients with diffuse large B-cell lymphoma (DLBCL). Blood 2003;101:4279-4284 [Duplicate publication]
112.	White,C. A. et al.Rituxan immunotherapy and Zevalin radioimmunotherapy in the treatment of non-Hodgkin's lymphoma. Current Pharmaceutical Biotechnology 2003;4:221-238 [Abstract/editorial/review/comments]
113.	Hiddemann,W. et al.Rituximab plus chemotherapy in follicular and mantle cell lymphomas Seminars in oncology 2003;30:16-20 [Abstract/editorial/review/comments]
114.	Plosker,G. L. et al.Rituximab: a review of its use in non-Hodgkin's lymphoma and chronic lymphocytic leukaemia Drugs 2003;63:803-843 [Abstract/editorial/review/comments]
115.	van der Kolk,L. E. et al.Treatment of relapsed B-cell non-Hodgkin's lymphoma with a combination of chimeric anti-CD20 monoclonal antibodies (rituximab) and G-CSF: Final report on safety and efficacy. Leukemia 2003;17:1658-1664 [Non R free arm]
116.	Eckschlager,T. et al.Have immunotherapy place in current pediatric oncology? Klinicka Onkologie 2003;16:125-126 [Abstract/editorial/review/comments]
117.	Dillman,R. O. et al.Treatment of low-grade B-cell lymphoma with the monoclonal antibody rituximab. Seminars in oncology 2003;30:434-447 [Abstract/editorial/review/comments]
118.	Lin,T. S. et al.Rituximab in B-cell chronic lymphocytic leukemia. Seminars in oncology 2003;30:483-492 [Abstract/editorial/review/comments]
119.	Tobinai,K. et al.Radioimmunotherapy of intractable lymphoma. Japanese Journal of Clinical Radiology 2003;48:1187-1194 [Abstract/editorial/review/comments]
120.	O'Brien,S. M. et al.Alemtuzumab as Treatment for Residual Disease after Chemotherapy in Patients with Chronic Lymphocytic Leukemia. Cancer 2003;98:2657-2663 [Other MoAb included]
121.	Hernandez-Ilizaliturri,F. J. et al.Neutrophils Contribute to the Biological Antitumor Activity of Rituximab in a Non-Hodgkin's Lymphoma Severe Combined Immunodeficiency Mouse Model. Clinical Cancer Research 2003;9:5866-5873 [Abstract/editorial/review/comments]
122.	Buchele,T. et al.Proapoptotic therapy with oblimersen (bcl-2 antisense oligonucleotide) - Review of preclinical and clinical results. Onkologie 2003;26:60-69 [Abstract/editorial/review/comments]
123.	Hagenbeek,A. et al.Radioimmunotherapy for NHL: Experience of 90Y-Ibritumomab tiuxetan in clinical practice. Leukemia and Lymphoma 2003;44:37s-47s [Other MoAb included]
124.	Rehwald,U. et al.Treatment of relapsed CD20+ Hodgkin lymphoma with the monoclonal antibody rituximab is effective and well tolerated: results of a phase 2 trial of the German Hodgkin Lymphoma Study Group Blood 2003;101:420-424 [Non randomized studies]
125.	Faderl,S. et al.Experience with alemtuzumab plus rituximab in patients with relapsed and refractory lymphoid malignancies Blood 2003;101:3413-3415 [Other MoAb included]
126.	Ekstrand,B. C. et al.Rituximab in lymphocyte-predominant Hodgkin disease: results of a phase 2 trial Blood 2003;101:4285-4289 [Non randomized studies]
127.	Wiseman,G. A. et al.Radiation dosimetry results and safety correlations from 90Y-ibritumomab tiuxetan radioimmunotherapy for relapsed or refractory non-Hodgkin's lymphoma: combined data from 4 clinical trials Journal of nuclear medicine  2003;44:465-474 [Other MoAb included]
128.	Nieva,J. et al.Phase 2 study of rituximab in the treatment of cladribine-failed patients with hairy cell leukemia Blood 2003;102:810-813 [Non randomized studies]
129.	Gianni,A. M. et al.Long-term remission in mantle cell lymphoma following high-dose sequential chemotherapy and in vivo rituximab-purged stem cell autografting (R-HDS regimen) Blood 2003;102:749-755 [Non randomized studies]
130.	Savage,D. G. et al.Combined fludarabine and rituximab for low grade lymphoma and chronic lymphocytic leukemia Leukemia & lymphoma 2003;44:477-481 [Non randomized studies]
131.	Frankel,A. E. et al.DAB389IL2 (ONTAK) fusion protein therapy of chronic lymphocytic leukaemia Expert opinion on biological therapy 2003;3:179-186 [Non R free arm]
132.	Hainsworth,J. D. et al.Single-agent rituximab as first-line and maintenance treatment for patients with chronic lymphocytic leukemia or small lymphocytic lymphoma: a phase II trial of the Minnie Pearl Cancer Research Network Journal of clinical oncology  2003;21:1746-1751 [Non randomized studies]
133.	Hess,G. et al.Safety and feasibility of CHOP/rituximab induction treatment followed by high-dose chemo/radiotherapy and autologous PBSC-transplantation in patients with previously untreated mantle cell or indolent B-cell-non-Hodgkin's lymphoma Bone marrow transplantation 2003;31:775-782 [Non randomized studies]
134.	Joyce,R. M. et al.A phase I-II study of rituximab, ifosfamide, mitoxantrone and etoposide (R-IME) for B cell non-Hodgkin's lymphoma prior to and after high-dose chemotherapy and autologous stem cell transplantation (HDC-ASCT) Annals of Oncology  2003;:-7 [Non randomized studies]
135.	Press,O. W. et al.A phase 2 trial of CHOP chemotherapy followed by tositumomab/iodine I 131 tositumomab for previously untreated follicular non-Hodgkin lymphoma: Southwest Oncology Group Protocol S9911 Blood 2003;102:1606-1612 [Other MoAb included]
136.	Economopoulos,T. et al.Rituximab in combination with CNOP chemotherapy in patients with previously untreated indolent non-Hodgkin's lymphoma The hematology journal  2003;4:110-115 [Non randomized studies]
137.	Kami,M. et al.Safety of rituximab in lymphoma patients with hepatitis B or hepatitis C virus infection The hematology journal  2003;4:159-162 [Abstract/editorial/review/comments]
138.	Thomas,D. A. et al.Rituximab in relapsed or refractory hairy cell leukemia Blood 2003;102:3906-3911 [Non randomized studies]
139.	Hainsworth,J. D. et al.First-line treatment with brief-duration chemotherapy plus rituximab in elderly patients with intermediate-grade non-Hodgkin's lymphoma: phase II trial Clinical lymphoma 2003;4:36-42 [Non randomized studies]
140.	Conconi,A. et al.Clinical activity of rituximab in extranodal marginal zone B-cell lymphoma of MALT type Blood 2003;102:2741-2745 [Non randomized studies]
141.	Younes,A. et al.A pilot study of rituximab in patients with recurrent, classic Hodgkin disease Cancer 2003;98:310-314 [Non randomized studies]
142.	van der Kolk,L. E. et al.Treatment of relapsed B-cell non-Hodgkin's lymphoma with a combination of chimeric anti-CD20 monoclonal antibodies (rituximab) and G-CSF: final report on safety and efficacy Leukemia  2003;17:1658-1664 [Non randomized studies]
143.	Friedberg,J. W. et al.Ex vivo B cell depletion using the Eligix B Cell SC system and autologous peripheral blood stem cell transplantation in patients with follicular non-Hodgkin's lymphoma Bone marrow transplantation 2003;32:681-686 [Non R free arm]
144.	Martinelli,G. et al.Chlorambucil in combination with induction and maintenance rituximab is feasible and active in indolent non-Hodgkin's lymphoma British journal of haematology 2003;123:271-277 [Non randomized studies]
145.	Bremer,K. et al.Semi-extended, six weekly rituximab infusions in pre-treated advanced low-grade B cell non-Hodgkin's lymphoma: a phase II study Anti-Cancer Drugs 2003;14:809-815 [Non randomized studies]
146.	Cocconi,G. et al.P-CHOP: cisplatin (P) added to the standard CHOP regimen as first-line treatment for aggressive non-Hodgkin lymphoma: a single-institution phase II study American journal of clinical oncology 2003;26:535-542 [Non R free arm]
147.	Drapkin,R. et al.Results of a phase II multicenter trial of pentostatin and rituximab in patients with low grade B-cell non-Hodgkin's lymphoma: an effective and minimally toxic regimen Clinical lymphoma 2003;4:169-175 [Non randomized studies]
148.	Musto,P. et al.Short progression-free survival in myeloma patients receiving rituximab as maintenance therapy after autologous transplantation British journal of haematology 2003;123:746-747 [Purging, maintenance and sequential protocols]
149.	Dimopoulos,M. A. et al.Treatment of Waldenstrom's macroglobulinemia with the combination of fludarabine and cyclophosphamide Leukemia & lymphoma 2003;44:993-996 [Abstract/editorial/review/comments]
150.	Tobinai,K. et al.Clinical trials for malignant lymphoma in Japan. Japanese journal of clinical oncology 2004;34:369-378 [Abstract/editorial/review/comments]
151.	Ghielmini,M. et al.Prolonged treatment with rituximab in patients with follicular lymphoma significantly increases event-free survival and response duration compared with the standard weekly x 4 schedule Blood 2004;103:4416-4423 [Non R free arm]
152.	Zinzani,P. L. et al.Fludarabine plus mitoxantrone with and without rituximab versus CHOP with and without rituximab as front-line treatment for patients with follicular lymphoma Journal of clinical oncology  2004;22:2654-2661 [Non R free arm]
153.	Gordon,L. I. et al.Yttrium 90-labeled ibritumomab tiuxetan radioimmunotherapy produces high response rates and durable remissions in patients with previously treated B-cell lymphoma Clinical lymphoma 2004;5:98-101 [Other MoAb included]
154.	Williams,M. E. et al.ECOG 4402: randomized phase III-trial comparing two different rituximab dosing regimens for patients with low tumor burden indolent non-Hodgkin's lymphoma Current hematology reports 2004;3:395-396 [Non R free arm]
155.	Adachi,S. et al.Apoptosis induced by molecular targeting therapy in hematological malignancies. Acta Haematologica 2004;111:107-123 [Abstract/editorial/review/comments]
156.	Jantunen,E. et al.Stem cell transplantation for peripheral T-cell lymphomas. Leukemia and Lymphoma 2004;45:441-446 [Abstract/editorial/review/comments]
157.	Hainsworth,J. D. et al.Prolonging Remission with Rituximab Maintenance Therapy. Seminars in oncology 2004;31:17-21 [Abstract/editorial/review/comments]
158.	Hillmen,P. et al.Advancing Therapy for Chronic Lymphocytic Leukemia - The Role of Rituximab. Seminars in oncology 2004;31:22-26 [Abstract/editorial/review/comments]
159.	Horwitz,S. M. et al.Rituximab as adjuvant to high-dose therapy and autologous hematopoietic cell transplantation for aggressive non-Hodgkin lymphoma Blood 2004;103:777-783 [Non randomized studies]
160.	Farag,S. S. et al.Fc gamma RIIIa and Fc gamma RIIa polymorphisms do not predict response to rituximab in B-cell chronic lymphocytic leukemia Blood 2004;103:1472-1474 [Non R free arm]
161.	Kewalramani,T. et al.Rituximab and ICE as second-line therapy before autologous stem cell transplantation for relapsed or primary refractory diffuse large B-cell lymphoma Blood 2004;103:3684-3688 [Non randomized studies]
162.	Endo,T. et al.Peripheral blood stem cell mobilization following CHOP plus rituximab therapy combined with G-CSF in patients with B-cell non-Hodgkin's lymphoma Bone marrow transplantation 2004;33:703-707 [Purging, maintenance and sequential protocols]
163.	Mangel,J. et al.Intensive chemotherapy and autologous stem-cell transplantation plus rituximab is superior to conventional chemotherapy for newly diagnosed advanced stage mantle-cell lymphoma: a matched pair analysis Annals of Oncology  2004;15:283-290 [Non randomized studies]
164.	Wohrer,S. et al.Rituximab, cyclophosphamide, doxorubicin, vincristine and prednisone (R-CHOP) for treatment of early-stage gastric diffuse large B-cell lymphoma. Annals of Oncology 2004;15:1086-1090 [Non randomized studies]
165.	Khouri,I. F. et al.Nonmyeloablative stem cell transplantation for lymphoma Seminars in oncology 2004;31:22-26 [Non R free arm]
166.	Jermann,M. et al.Rituximab-EPOCH, an effective salvage therapy for relapsed, refractory or transformed B-cell lymphomas: results of a phase II study Annals of Oncology  2004;15:511-516 [Non randomized studies]
167.	Ma,S. Y. et al.Fludarabine, mitoxantrone and dexamethasone in the treatment of indolent B- and T-cell lymphoid malignancies in Chinese patients British journal of haematology 2004;124:754-761 [Non R free arm]
168.	Gluck,W. L. et al.Phase I studies of interleukin (IL)-2 and rituximab in B-cell non-hodgkin's lymphoma: IL-2 mediated natural killer cell expansion correlations with clinical response Clinical cancer research  2004;10:2253-2264 [Non randomized studies]
169.	Tsiara,S. N. et al.Treatment of resistant/relapsing chronic lymphocytic leukemia with a combination regimen containing deoxycoformycin and rituximab Acta Haematologica 2004;111:185-188 [Non R free arm]
170.	Kaufmann,H. et al.Antitumor activity of rituximab plus thalidomide in patients with relapsed/refractory mantle cell lymphoma Blood 2004;104:2269-2271 [Non R free arm]
171.	Arcaini,L. et al.A model of in vivo purging with Rituximab and high-dose AraC in follicular and mantle cell lymphoma Bone marrow transplantation 2004;34:175-179 [Non randomized studies]
172.	Escalon,M. P. et al.Nonmyeloablative allogeneic hematopoietic transplantation: a promising salvage therapy for patients with non-Hodgkin's lymphoma whose disease has failed a prior autologous transplantation Journal of clinical oncology  2004;22:2419-2423 [Non randomized studies]
173.	Addeo,R. et al.Oxaliplatin/rituximab combination in the treatment of intermediate-low grade non-Hodgkin's lymphoma of elderly patients Oncology reports 2004;12:135-140 [Non randomized studies]
174.	Wohrer,S. et al.Rituximab, cyclophosphamide, doxorubicin, vincristine and prednisone (R-CHOP) for treatment of early-stage gastric diffuse large B-cell lymphoma Annals of Oncology  2004;15:1086-1090 [Non randomized studies]
175.	Lenz,G. et al.The role of fludarabine in the treatment of follicular and mantle cell lymphoma. Cancer 2004;101:883-893 [Abstract/editorial/review/comments]
176.	Niitsu,N. et al.Phase I study of Rituximab-CHOP regimen in combination with granulocyte colony-stimulating factor in patients with follicular lymphoma Clinical cancer research  2004;10:4077-4082 [Non randomized studies]
177.	Hendry,L. et al.Fludarabine, cyclophosphamide and mitoxantrone in relapsed or refractory chronic lymphocytic leukemia and low grade non-Hodgkin's lymphoma Leukemia & lymphoma 2004;45:945-950 [Non R free arm]
178.	Leonard,J. P. et al.Epratuzumab, a humanized anti-CD22 antibody, in aggressive non-Hodgkin's lymphoma: phase I/II clinical trial results Clinical cancer research  2004;10:5327-5334 [Other MoAb included]
179.	Leo,E. et al.Significant thrombocytopenia associated with the addition of rituximab to a combination of fludarabine and cyclophosphamide in the treatment of relapsed follicular lymphoma European journal of haematology 2004;73:251-257 [Non randomized studies]
180.	Dang,N. H. et al.Phase II study of denileukin diftitox for relapsed/refractory B-Cell non-Hodgkin's lymphoma Journal of clinical oncology  2004;22:4095-4102 [Non randomized studies]
181.	Eisenbeis,C. F. et al.Combination immunotherapy of B-cell non-Hodgkin's lymphoma with rituximab and interleukin-2: a preclinical and phase I study Clinical cancer research  2004;10:6101-6110 [Non randomized studies]
182.	Czuczman,M. S. et al.Prolonged clinical and molecular remission in patients with low-grade or follicular non-Hodgkin's lymphoma treated with rituximab plus CHOP chemotherapy: 9-year follow-up Journal of clinical oncology  2004;22:4711-4716 [Non randomized studies]
183.	Jabbour,E. et al.Stem cell transplantation for chronic lymphocytic leukemia: Should not more patients get a transplant?. Bone marrow transplantation 2004;34:289-297 [Abstract/editorial/review/comments]
184.	Nabhan,C. et al.A pilot trial of rituximab and alemtuzumab combination therapy in patients with relapsed and/or refractory chronic lymphocytic leukemia (CLL) Leukemia & lymphoma 2004;45:2269-2273 [Other MoAb included]
185.	Brugger,W. et al.Rituximab consolidation after high-dose chemotherapy and autologous blood stem cell transplantation in follicular and mantle cell lymphoma: a prospective, multicenter phase II study Annals of Oncology  2004;15:1691-1698 [Non randomized studies]
186.	Cervetti,G. et al.Rituximab as treatment for minimal residual disease in hairy cell leukaemia European journal of haematology 2004;73:412-417 [Non randomized studies]
187.	Weide,R. et al.Bendamustine/Mitoxantrone/Rituximab (BMR): a very effective, well tolerated outpatient chemoimmunotherapy for relapsed and refractory CD20-positive indolent malignancies. Final results of a pilot study Leukemia & lymphoma 2004;45:2445-2449 [Non randomized studies]
188.	Kim,R. et al.Therapeutic potential of antisense Bcl-2 as a chemosensitizer for cancer therapy. Cancer 2004;101:2491-2502 [Abstract/editorial/review/comments]
189.	Knight,C. et al.Rituximab (MabThera) for aggressive non-Hodgkin's lymphoma: Systematic review and economic evaluation. Health technology assessment 2004;8:48- [Abstract/editorial/review/comments]
190.	Aurer,I. et al.Rituximab in the treatment of indolent non-Hodgkin's lymphoma Lijecnicki vjesnik 2004;126:307-311 [Abstract/editorial/review/comments]
191.	Mariette,X. et al.Emerging biological therapies in rheumatoid arthritis. Joint Bone Spine 2004;71:470-474 [Abstract/editorial/review/comments]
192.	Bjorkholm,M. et al.Treatment options in Waldenstrom's macroglobulinemia. Clinical Lymphoma 2004;5:155-162 [Abstract/editorial/review/comments]
193.	Budziszewska,B. K. et al.Management of diffuse large B-cell lymphomas - Three case reports. Onkologia Polska 2004;7:199-205 [Non R free arm]
194.	Clayton,A. et al.The changing face of HIV-associated lymphoma: What can we learn about optimal therapy in the post highly active antiretroviral therapy era?. Hematological oncology 2004;22:111-120 [Abstract/editorial/review/comments]
195.	Looney,R. J. et al.B cells as therapeutic targets for rheumatic diseases. Current opinion in rheumatology 2004;16:180-185 [Abstract/editorial/review/comments]
196.	Leblond,V. et al.Waldenstrom's macroglobulinemia: Prognostic factors and recent therapeutic advances. Clinical and Experimental Medicine 2004;3:187-198 [Abstract/editorial/review/comments]
197.	Viklicky,O. et al.Monoclonal antibodies in kidney transplantation. Aktuality v Nefrologii 2004;10:25-30 [Abstract/editorial/review/comments]
198.	Messori,A. et al.Survival gain with new drugs. PharmacoEconomics - Italian Research Articles 2004;6:95-104 [Abstract/editorial/review/comments]
199.	Niitsu,N. et al.Phase I study of rituximab-CHOP regimen in combination with granulocyte colony-stimulating factor in patients with follicular lymphoma. Clinical Cancer Research 2004;10:4077-4082 [Non randomized studies]
200.	Tobinai,K. et al.Japanese multicenter phase II and pharmacokinetic study of rituximab in relapsed or refractory patients with aggressive B-cell lymphoma Annals of Oncology  2004;15:821-830 [Non randomized studies]
201.	Tsimberidou,A. M. et al.Yttrium-90 ibritumomab tiuxetan radioimmunotherapy in Richter syndrome Cancer 2004;100:2195-2200 [Other MoAb included]
202.	Gertz,M. A. et al.Multicenter phase 2 trial of rituximab for Waldenstrom macroglobulinemia (WM): an Eastern Cooperative Oncology Group Study (E3A98) Leukemia & lymphoma 2004;45:2047-2055 [Non randomized studies]
203.	Ghobrial,I. M. et al.Initial immunoglobulin M 'flare' after rituximab therapy in patients diagnosed with Waldenstrom macroglobulinemia: an Eastern Cooperative Oncology Group Study Cancer 2004;101:2593-2598 [Non randomized studies]
204.	Dimopoulos,M. A. et al.Treatment of Waldenstrom's macroglobulinemia with rituximab: prognostic factors for response and progression Leukemia & lymphoma 2004;45:2057-2061 [Non randomized studies]
205.	Bociek,R. G. et al.Adult Burkitt's lymphoma. Clinical Lymphoma and Myeloma 2005;6:11-20 [Abstract/editorial/review/comments]
206.	Feugier,P. et al.Long-term results of the R-CHOP study in the treatment of elderly patients with diffuse large B-cell lymphoma: A study by the groupe d'etude des lymphomes de l'adulte. Journal of Clinical Oncology 2005;23:4117-4126 [Duplicate publication]
207.	Dimopoulos,M. A. et al.Diagnosis and management of Waldenstrom's macroglobulinemia. Journal of Clinical Oncology 2005;23:1564-1577 [Abstract/editorial/review/comments]
208.	Romaguera,J. E. et al.High rate of durable remissions after treatment of newly diagnosed aggressive mantle-cell lymphoma with rituximab plus hyper-CVAD alternating with rituximab plus high-dose methotrexate and cytarabine. Journal of Clinical Oncology 2005;23:7013-7023 [Non R free arm]
209.	Habermann,T. M. et al.Advances in the treatment of aggessive lymphomas. Part 3 of a 3-part series: Advances in the treatment of hematologic maliganancies. Clinical Advances in Hematology and Oncology 2005;3:13- [Abstract/editorial/review/comments]
210.	Ghielmini,M. et al.Effect of single-agent rituximab given at the standard schedule or as prolonged treatment in patients with mantle cell lymphoma: a study of the Swiss Group for Clinical Cancer Research (SAKK) Journal of clinical oncology  2005;23:705-711 [Purging, maintenance and sequential protocols]
211.	Hainsworth,J. D. et al.Maximizing therapeutic benefit of rituximab: maintenance therapy versus re-treatment at progression in patients with indolent non-Hodgkin's lymphoma--a randomized phase II trial of the Minnie Pearl Cancer Research Network Journal of clinical oncology  2005;23:1088-1095 [Non randomized studies]
212.	Kim,S. et al.Prospective randomized comparative observation of single- vs split-dose lenograstim to mobilize peripheral blood progenitor cells following chemotherapy in patients with multiple myeloma or non-Hodgkin's lymphoma Annals of Hematology 2005;84:742-747 [Non R free arm]
213.	Ghielmini,M. et al.Adding rituximab to cyclophosphamide, vincristine and prednisone increases time to treatment failure or progression in people with untreated stage III/IV follicular lymphoma Cancer treatment reviews 2005;31:644-647 [Duplicate publication]
214.	Fisher,R. I. et al.Overview of Southwest Oncology Group Clinical Trials in non-Hodgkin Lymphoma. S0016. A phase III trial of CHOP vs CHOP + rituximab vs CHOP + iodine131-labeled monoclonal anti-B1 antibody (tositumomab) for treatment of newly diagnosed follicular NHL Clinical advances in hematology & oncology  2005;3:544-546 [Other MoAb included]
215.	Lin,T. Y. et al.Comparison between R-CHOP regimen and CHOP regimen in treating naive diffuse large B-cell lymphoma in China--a multi-center randomized trail Ai zheng  2005;24:1421-1426 [Chinese language]
216.	Lin,T. S. et al.FCGR3A and FCGR2A polymorphisms may not correlate with response to alemtuzumab in chronic lymphocytic leukemia Blood 2005;105:289-291 [Other MoAb included]
217.	Friedberg,J. W. et al.Combination immunotherapy with a CpG oligonucleotide (1018 ISS) and rituximab in patients with non-Hodgkin lymphoma: increased interferon-alpha/beta-inducible gene expression, without significant toxicity Blood 2005;105:489-495 [Non randomized studies]
218.	Spina,M. et al.Rituximab plus infusional cyclophosphamide, doxorubicin, and etoposide in HIV-associated non-Hodgkin lymphoma: pooled results from 3 phase 2 trials Blood 2005;105:1891-1897 [Abstract/editorial/review/comments]
219.	Horning,S. J. et al.Efficacy and safety of tositumomab and iodine-131 tositumomab (Bexxar) in B-cell lymphoma, progressive after rituximab Journal of clinical oncology  2005;23:712-719 [Other MoAb included]
220.	O'Connor,O. A. et al.Phase II clinical experience with the novel proteasome inhibitor bortezomib in patients with indolent non-Hodgkin's lymphoma and mantle cell lymphoma Journal of clinical oncology  2005;23:676-684 [Other MoAb included]
221.	Wenger,C. et al.Rituximab plus gemcitabine: a therapeutic option for elderly or frail patients with aggressive non Hodgkin's lymphoma? Leukemia & lymphoma 2005;46:71-75 [Non randomized studies]
222.	Biagi,J. J. et al.A phase II study of dexamethasone, ifosfamide, cisplatin and etoposide (DICE) as salvage chemotherapy for patients with relapsed and refractory lymphoma Leukemia & lymphoma 2005;46:197-206 [Non R free arm]
223.	Hainsworth,J. D. et al.Rituximab plus short-duration chemotherapy as first-line treatment for follicular non-Hodgkin's lymphoma: a phase II trial of the minnie pearl cancer research network Journal of clinical oncology  2005;23:1500-1506 [Non randomized studies]
224.	Gordan,L. N. et al.Phase II trial of individualized rituximab dosing for patients with CD20-positive lymphoproliferative disorders Journal of clinical oncology  2005;23:1096-1102 [Non randomized studies]
225.	Witzig,T. E. et al.Rituximab therapy for patients with newly diagnosed, advanced-stage, follicular grade I non-Hodgkin's lymphoma: a phase II trial in the North Central Cancer Treatment Group Journal of clinical oncology  2005;23:1103-1108 [Non randomized studies]
226.	Martinelli,G. et al.Clinical activity of rituximab in gastric marginal zone non-Hodgkin's lymphoma resistant to or not eligible for anti-Helicobacter pylori therapy Journal of clinical oncology  2005;23:1979-1983 [Non R free arm]
227.	Di Bella,N. et al.An open-label pilot study of pentostatin, mitoxantrone, and rituximab in patients with previously untreated, Stage III or IV, low-grade non-Hodgkin lymphoma Cancer 2005;103:978-984 [Non randomized studies]
228.	Czuczman,M. S. et al.Rituximab in combination with fludarabine chemotherapy in low-grade or follicular lymphoma Journal of clinical oncology  2005;23:694-704 [Non randomized studies]
229.	Niitsu,N. et al.Phase I/II study of the rituximab-EPOCT regimen in combination with granulocyte colony-stimulating factor in patients with relapsed or refractory follicular lymphoma including evaluation of its cardiotoxicity using B-type natriuretic peptide and troponin Clinical cancer research  2005;11:697-702 [Non randomized studies]
230.	McLaughlin,P. et al.Myelodysplasia and acute myeloid leukemia following therapy for indolent lymphoma with fludarabine, mitoxantrone, and dexamethasone (FND) plus rituximab and interferon alpha Blood 2005;105:4573-4575 [Non R free arm]
231.	Cortelezzi,A. et al.A pilot study of low-dose subcutaneous alemtuzumab therapy for patients with hemotherapy-refractory chronic lymphocytic leukemia Haematologica 2005;90:410-412 [Other MoAb included]
232.	Keating,M. J. et al.Early results of a chemoimmunotherapy regimen of fludarabine, cyclophosphamide, and rituximab as initial therapy for chronic lymphocytic leukemia Journal of clinical oncology  2005;23:4079-4088 [Non randomized studies]
233.	Khouri,I. F. et al.Concurrent administration of high-dose rituximab before and after autologous stem-cell transplantation for relapsed aggressive B-cell non-Hodgkin's lymphomas Journal of clinical oncology  2005;23:2240-2247 [Abstract/editorial/review/comments]
234.	Boucek,J. A. et al.Validation of prospective whole-body bone marrow dosimetry by SPECT/CT multimodality imaging in (131)I-anti-CD20 rituximab radioimmunotherapy of non-Hodgkin's lymphoma European journal of nuclear medicine and molecular imaging 2005;32:458-469 [Other MoAb included]
235.	Antonescu,C. et al.Repeated injections of 131I-rituximab show patient-specific stable biodistribution and tissue kinetics European journal of nuclear medicine and molecular imaging 2005;32:943-951 [Other MoAb included]
236.	Haioun,C. et al.18F]fluoro-2-deoxy-D-glucose positron emission tomography (FDG-PET) in aggressive lymphoma: an early prognostic tool for predicting patient outcome Blood 2005;106:1376-1381 [Non R free arm]
237.	Rummel,M. J. et al.Bendamustine plus rituximab is effective and has a favorable toxicity profile in the treatment of mantle cell and low-grade non-Hodgkin's lymphoma Journal of clinical oncology  2005;23:3383-3389 [Non randomized studies]
238.	Bienert,M. et al.Radioimmunotherapy using 131I-rituximab in patients with advanced stage B-cell non-Hodgkin's lymphoma: initial experience European journal of nuclear medicine and molecular imaging 2005;32:1225-1233 [Other MoAb included]
239.	Josting,A. et al.High-dose sequential chemotherapy followed by autologous stem cell transplantation in relapsed and refractory aggressive non-Hodgkin's lymphoma: results of a multicenter phase II study Annals of Oncology  2005;16:1359-1365 [Non R free arm]
240.	Leonard,J. P. et al.Combination antibody therapy with epratuzumab and rituximab in relapsed or refractory non-Hodgkin's lymphoma Journal of clinical oncology  2005;23:5044-5051 [Other MoAb included]
241.	Nademanee,A. et al.A phase 1/2 trial of high-dose yttrium-90-ibritumomab tiuxetan in combination with high-dose etoposide and cyclophosphamide followed by autologous stem cell transplantation in patients with poor-risk or relapsed non-Hodgkin lymphoma Blood 2005;106:2896-2902 [Other MoAb included]
242.	Halaas,J. L. et al.R-CHOP-14 in patients with diffuse large B-cell lymphoma: feasibility and preliminary efficacy Leukemia & lymphoma 2005;46:541-547 [Non randomized studies]
243.	Grigg,A. P. et al.Autografting followed by rituximab for chemosensitive mantle cell lymphoma: a pilot study and literature review Leukemia & lymphoma 2005;46:851-860 [Non R free arm]
244.	Ghielmini,M. et al.Single agent rituximab in patients with follicular or mantle cell lymphoma: clinical and biological factors that are predictive of response and event-free survival as well as the effect of rituximab on the immune system: a study of the Swiss Group for Cli Annals of Oncology  2005;16:1675-1682 [Purging, maintenance and sequential protocols]
245.	Pijpe,J. et al.Rituximab treatment in patients with primary Sjogren's syndrome: an open-label phase II study Arthritis and Rheumatism 2005;52:2740-2750 [Non lymphoma ]
246.	Romaguera,J. E. et al.High rate of durable remissions after treatment of newly diagnosed aggressive mantle-cell lymphoma with rituximab plus hyper-CVAD alternating with rituximab plus high-dose methotrexate and cytarabine Journal of clinical oncology  2005;23:7013-7023 [Non R free arm]
247.	Blaes,A. H. et al.Rituximab therapy is effective for posttransplant lymphoproliferative disorders after solid organ transplantation: results of a phase II trial Cancer 2005;104:1661-1667 [Non randomized studies]
248.	Grillo-Lopez,A. J. et al.Is rituximab effective as a single agent in mantle-cell lymphoma? Nature clinical practice.Oncology 2005;2:500-501 [Abstract/editorial/review/comments]
249.	Bauwens,D. et al.Activity and safety of combined rituximab with chlorambucil in patients with mantle cell lymphoma British journal of haematology 2005;131:338-340 [Non randomized studies]
250.	Hensel,M. et al.Pentostatin/cyclophosphamide with or without rituximab: an effective regimen for patients with Waldenstrom's macroglobulinemia/lymphoplasmacytic lymphoma Clinical lymphoma & myeloma 2005;6:131-135 [Non lymphoma ]
251.	Vose,J. M. et al.Long-term update of a phase II study of rituximab in combination with CHOP chemotherapy in patients with previously untreated, aggressive non-Hodgkin's lymphoma Leukemia & lymphoma 2005;46:1569-1573 [Non randomized studies]
252.	Lenz,G. et al.Does the combination of rituximab and thalidomide influence the long-term perspectives of advanced-stage MCL? Nature clinical practice.Oncology 2005;2:72-73 [Non randomized studies]
253.	Del Poeta,G. et al.The addition of rituximab to fludarabine improves clinical outcome in untreated patients with ZAP-70-negative chronic lymphocytic leukemia Cancer 2005;104:2743-2752 [Non randomized studies]
254.	Cervetti,G. et al.High efficacy of Rituximab in indolent HCV-related lymphoproliferative disorders associated with systemic autoimmune diseases Clinical and experimental rheumatology 2005;23:877-880 [Non R free arm]
255.	Byrd,J. C. et al.Addition of rituximab to fludarabine may prolong progression-free survival and overall survival in patients with previously untreated chronic lymphocytic leukemia: An updated retrospective comparative analysis of CALGB 9712 and CALGB 9011. Blood 2005;105:49-53 [Non randomized studies]
256.	Robak,T. et al.The place of cladribine in the treatment of chronic lymphocytic leukemia: A 10-year experience in Poland. Annals of Hematology 2005;84:63-70 [Abstract/editorial/review/comments]
257.	Paneesha,S. et al.Stem cell transplantation for chronic lymphocytic leukaemia. British journal of haematology 2005;128:145-152 [Abstract/editorial/review/comments]
258.	Coiffier,B. et al.Monoclonal antibodies in the treatment of indolent lymphomas. Best Practice and Research in Clinical Haematology 2005;18:69-80 [Abstract/editorial/review/comments]
259.	Avivi,I. et al.Conventional allograft and autograft in low grade lymphoma. Best Practice and Research in Clinical Haematology 2005;18:113-128 [Abstract/editorial/review/comments]
260.	Reyes,F. et al.ACVBP versus CHOP plus radiotherapy for localized aggressive lymphoma. New England Journal of Medicine 2005;352:1197-1205 [Non R free arm]
261.	Lopez,A. et al.Pegfilgrastim supports delivery of CHO-R chemotherapy administered every 14 days: A randomized phase II study. Journal of Supportive Oncology 2005;3:46-47 [Abstract/editorial/review/comments]
262.	Satwani,P. et al.Reduced-intensity allogeneic stem cell transplantation in adults and children with malignant and nonmalignant diseases: End of the beginning and future challenges. Biology of Blood and Marrow Transplantation 2005;11:403-422 [Abstract/editorial/review/comments]
263.	Lin,X. -F et al.Evidence-based treatment for newly diagnosed diffuse large-B-cell lymphoma in an elderly patient. Chinese Journal of Evidence-Based Medicine 2005;5:347-349 [Abstract/editorial/review/comments]
264.	Leitch,H. A. et al.Vaccine therapy for non-Hodgkin's lymphoma and other B-cell malignancies. Current Opinion in Investigational Drugs 2005;6:597-604 [Abstract/editorial/review/comments]
265.	Van Hoof,A. et al.Hodkgin's disease: Current insights. Tijdschrift voor Geneeskunde 2005;61:822-829 [Abstract/editorial/review/comments]
266.	Hurvitz,S. A. et al.Recombinant, tumour-derived idiotype vaccination for indolent B cell non-Hodgkin's lymphomas: A focus on FavId[trademark]. Expert Opinion on Biological Therapy 2005;5:841-852 [Abstract/editorial/review/comments]
267.	Ljungman,P. et al.Vaccination of patients with haematological malignancies with one or two doses of influenza vaccine: A randomised study. British journal of haematology 2005;130:96-98 [Non R free arm]
268.	Crump,M. et al.A randomized phase III study of gemcitabine, dexamethasone, and cisplatin versus dexamethasone, cytarabine, and cisplatin as salvage chemotherapy followed by posttransplantation rituximab maintenance therapy versus observation for treatment of aggressive Clinical Lymphoma 2005;6:56-60 [Abstract/editorial/review/comments]
269.	DeNardo,G. L. et al.Treatment of Non-Hodgkin's Lymphoma (NHL) with radiolabeled antibodies (mAbs). Seminars in nuclear medicine 2005;35:202-211 [Other MoAb included]
270.	Hurvitz,S. A. et al.Current status of therapeutic vaccines for non-Hodgkin's lymphoma. Current opinion in oncology 2005;17:432-440 [Abstract/editorial/review/comments]
271.	Spina,M. et al.Rituximab for HIV-associated lymphoma: Weighing the benefits and risks. Current opinion in oncology 2005;17:462-465 [Abstract/editorial/review/comments]
272.	Ng,R. et al.Pegfilgrastim: Evidence in support of its use with cytotoxic chemotherapy. Expert Review of Anticancer Therapy 2005;5:585-590 [Abstract/editorial/review/comments]
273.	Frampton,J. E. et al.Pegfilgrastim: A review of its use in chemotherapy-induced neutropenia. American Journal of Cancer 2005;4:255-274 [Abstract/editorial/review/comments]
274.	Lamanna,N. et al.Advances in the treatment of chronic lymphocytic leukemia. Current oncology reports 2005;7:333-338 [Abstract/editorial/review/comments]
275.	Schlehuber,S. et al.Anticalins in drug development. BioDrugs 2005;19:279-288 [Abstract/editorial/review/comments]
276.	Robak,T. et al.Comparison of cladribine plus prednisone with chlorambucil plus prednisone in patients with chronic lymphocytic leukemia. Final report of the Polish Adult Leukemia Group (PALG CLL1). Medical Science Monitor 2005;11:71-79 [Non R free arm]
277.	Worker,S. et al.Routine application of the proton-pump inhibitor pantoprazole in patients with gastric lymphoma undergoing chemotherapy. Scandinavian journal of gastroenterology 2005;40:1222-1225 [Non R free arm]
278.	Cronin,D. P. et al.Patterns of care in a population-based random sample of patients diagnosed with non-Hodgkin's lymphoma. Hematological oncology 2005;23:73-81 [Abstract/editorial/review/comments]
279.	Robak,T. et al.Therapy of chronic lymphocytic leukaemia with purine nucleoside analogues: Facts and controversies. Drugs and Aging 2005;22:983-1012 [Abstract/editorial/review/comments]
280.	Eisenberg,R. et al.The therapeutic potential of anti-CD20: What do B-cells do?. Clinical Immunology 2005;117:207-213 [Abstract/editorial/review/comments]
281.	Gertz,M. A. et al.Early results of a phase I trial of oblimersen sodium for relapsed or refractory Waldenstrom's macroglobulinemia Clinical lymphoma 2005;5:282-284 [Non randomized studies]
282.	Treon,S. P. et al.Extended rituximab therapy in Waldenstrom's macroglobulinemia Annals of Oncology  2005;16:132-138 [Non randomized studies]
283.	Treon,S. P. et al.Polymorphisms in FcgammaRIIIA (CD16) receptor expression are associated with clinical response to rituximab in Waldenstrom's macroglobulinemia Journal of clinical oncology  2005;23:474-481 [Non randomized studies]
284.	Treon,S. P. et al.CHOP plus rituximab therapy in Waldenstrom's macroglobulinemia Clinical lymphoma 2005;5:273-277 [Non randomized studies]
285.	Dimopoulos,M. A. et al.Predictive factors for response to rituximab in Waldenstrom's macroglobulinemia Clinical lymphoma 2005;5:270-272 [Non randomized studies]
286.	Wierda,W. et al.A retrospective comparison of three sequential groups of patients with recurrent/refractory chronic lymphocytic leukemia treated with fludarabine-based regimens. Cancer 2006;106:337-345 [Non randomized studies]
287.	Dundar,Y. et al.Comparison of conference abstracts and presentations with full-text articles in the health technology assessments of rapidly evolving technologies. Health technology assessment 2006;10:57-ss [Abstract/editorial/review/comments]
288.	Van Heeckeren,W. J. et al.Randomised comparison of two B-cell purging protocols for patients with B-cell non-Hodgkin lymphoma: in vivo purging with rituximab versus ex vivo purging with CliniMACS CD34+ cell enrichment device. British journal of haematology 2006;132:42-55 [Purging, maintenance and sequential protocols]
289.	Coiffier,B. et al.Monoclonal antibody as therapy for malignant lymphomas. Comptes Rendus - Biologies 2006;329:241-254 [Abstract/editorial/review/comments]
290.	Robak,T. et al.New agents in chronic lymphocytic leukemia. Current Treatment Options in Oncology 2006;7:200-212 [Abstract/editorial/review/comments]
291.	Pfreundschuh,M. et al.CHOP-like chemotherapy plus rituximab versus CHOP-like chemotherapy alone in young patients with good-prognosis diffuse large-B-cell lymphoma: a randomised controlled trial by the MabThera International Trial (MInT) Group. Lancet Oncology 2006;7:379-391 [Non identical CHT in either arm]
292.	Cvetkovic,R. S. et al.Rituximab: A review of its use in non-Hodgkin's lymphoma and chronic lymphocytic leukaemia. Drugs 2006;66:791-820 [Abstract/editorial/review/comments]
293.	Hagberg,H. et al.Randomised phase III study of R-ICE versus R-DHAP in relapsed patients with CD20 diffuse large B-cell lymphoma (DLBCL) followed by high-dose therapy and a second randomisation to maintenance treatment with rituximab or not: An update of the CORAL study. Annals of Oncology 2006;17:31-32 [Non R free arm]
294.	Moskowitz,C. et al.Is it time to stop treating subsets of DLBCL with R-CHOP?. Blood 2006;107:4197-4198 [Abstract/editorial/review/comments]
295.	Jantunen,E. et al.Autologous stem cell transplantation in patients with chronic lymphocytic leukaemia: The Finnish experience. Bone marrow transplantation 2006;37:1093-1098 [Non R free arm]
296.	De Vita,S. et al.Treatment of rheumatoid arthritis with rituximab: An update and possible indications. Autoimmunity Reviews 2006;5:443-448 [Abstract/editorial/review/comments]
297.	Gore,S. D. et al.Six (or more) drugs in search of a mechanism: DNA methyltransferase and histone deacetylase inhibitors in the treatment of myelodysplastic syndromes. JNCCN Journal of the National Comprehensive Cancer Network 2006;4:83-90 [Abstract/editorial/review/comments]
298.	Held,G. et al.Rituximab for the treatment of diffuse large B-cell lymphomas. Expert Review of Anticancer Therapy 2006;6:1175-1186 [Abstract/editorial/review/comments]
299.	Weigert,O. et al.Investigational strategies in autologous stem cell transplantation for follicular lymphoma. Current oncology reports 2006;8:368-375 [Abstract/editorial/review/comments]
300.	Nademanee,A. et al.Role of Hematopoietic Stem Cell Transplantation for Advanced-Stage Diffuse Large Cell B-Cell Lymphoma-B. Seminars in hematology 2006;43:240-250 [Abstract/editorial/review/comments]
301.	Ladetto,M. et al.Prolonged survival and low incidence of late toxic sequelae in advanced follicular lymphoma treated with a TBI-free autografting program: Updated results of the multicenter consecutive GITMO trial. Leukemia 2006;20:1840-1847 [Non R free arm]
302.	Holmberg,L. A. et al.Immunotherapy with rituximab/interleukin-2 after autologous stem cell transplantation as treatment for CD20+ non-Hodgkin's lymphoma. Clinical Lymphoma and Myeloma 2006;7:135-139 [Non randomized studies]
303.	Tam,F. W. K. et al.Current pharmacotherapy for the treatment of crescentic glomerulonephritis. Expert opinion on investigational drugs 2006;15:1353-1369 [Abstract/editorial/review/comments]
304.	Lamanna,N. et al.Advances in the treatment of chronic lymphocytic leukemia. Current Hematologic Malignancy Reports 2006;1:43-48 [Abstract/editorial/review/comments]
305.	Frey,N. V. et al.Primary lymphomas of the cervix and uterus: The University of Pennsylvania's experience and a review of the literature. Leukemia and Lymphoma 2006;47:1894-1901 [Abstract/editorial/review/comments]
306.	Safdar,A. et al.Dose-related safety and immunogenicity of baculovirus-expressed trivalent influenza vaccine: A double-blind, controlled trial in adult patients with non-Hodgkin B cell lymphoma. Journal of Infectious Diseases 2006;194:1394-1397 [Non R free arm]
307.	Magda,D. et al.Motexafin gadolinium: A novel redox active drug for cancer therapy. Seminars in cancer biology 2006;16:466-476 [Abstract/editorial/review/comments]
308.	Wiernik,P. H. et al.Plasma cell dyscrasias and leukemias. Update on Cancer Therapeutics 2006;1:539-567 [Abstract/editorial/review/comments]
309.	Wilson,W. H. et al.Phase III randomized study of R-CHOP vs dose-adjusted EPOCH-R with molecular profiling in untreated de novo diffuse large B-cell lymphoma. Clinical Advances in Hematology and Oncology 2006;4:848-850 [Non R free arm]
310.	Eisenberg,R. et al.Targeting B cells in SLE: The experience with rituximab treatment (Anti-CD20). Endocrine, Metabolic and Immune Disorders - Drug Targets 2006;6:345-350 [Non lymphoma ]
311.	Robak,T. et al.Current treatment approaches in chronic lymphocytic leukemia. Acta Haematologica Polonica 2006;37:41-51 [Abstract/editorial/review/comments]
312.	Byrd,J. C. et al.Select high-risk genetic features predict earlier progression following chemoimmunotherapy with fludarabine and rituximab in chronic lymphocytic leukemia: Justification for risk-adapted therapy. Journal of Clinical Oncology 2006;24:437-443 [Non randomized studies]
313.	Provencio,M. et al.Rapid-infusion rituximab in lymphoma treatment Annals of Oncology  2006;17:1027-1028 [Non R free arm]
314.	Di Renzo,N. et al.Vinorelbine, gemcitabine, procarbazine and prednisone (ViGePP) as salvage therapy in relapsed or refractory aggressive non-Hodgkin's lymphoma (NHL): results of a phase II study conducted by the Gruppo Italiano per lo Studio dei Linfomi Leukemia & lymphoma 2006;47:473-479 [Non R free arm]
315.	Winter,J. N. et al.Prognostic significance of Bcl-6 protein expression in DLBCL treated with CHOP or R-CHOP: a prospective correlative study Blood 2006;107:4207-4213 [No infection outcome]
316.	Pfreundschuh,M. et al.Factors predictive for response of follicular and mantle-cell lymphoma to rituximab Nature clinical practice.Oncology 2006;3:184-185 [Abstract/editorial/review/comments]
317.	Kim,D. H. et al.FCGR3A gene polymorphisms may correlate with response to frontline R-CHOP therapy for diffuse large B-cell lymphoma Blood 2006;108:2720-2725 [Non randomized studies]
318.	Ogura,M. et al.Randomized phase II study of concurrent and sequential rituximab and CHOP chemotherapy in untreated indolent B-cell lymphoma Cancer science 2006;97:305-312 [Purging, maintenance and sequential protocols]
319.	Buske,C. et al.The Follicular Lymphoma International Prognostic Index (FLIPI) separates high-risk from intermediate- or low-risk patients with advanced-stage follicular lymphoma treated front-line with rituximab and the combination of cyclophosphamide, doxorubicin, vinc Blood 2006;108:1504-1508 [Abstract/editorial/review/comments]
320.	Nickenig,C. et al.Combined cyclophosphamide, vincristine, doxorubicin, and prednisone (CHOP) improves response rates but not survival and has lower hematologic toxicity compared with combined mitoxantrone, chlorambucil, and prednisone (MCP) in follicular and mantle cell ly Cancer 2006;107:1014-1022 [Non R free arm]
321.	Ansell,S. M. et al.Randomized phase II study of interleukin-12 in combination with rituximab in previously treated non-Hodgkin's lymphoma patients Clinical cancer research  2006;12:6056-6063 [Non R free arm]
322.	van Oers,M. H. et al.Rituximab maintenance improves clinical outcome of relapsed/resistant follicular non-Hodgkin lymphoma in patients both with and without rituximab during induction: results of a prospective randomized phase 3 intergroup trial Blood 2006;108:3295-3301 [Duplicate publication]
323.	Hess,G. et al.Effect of rituximab on the long-term outcome after high-dose therapy for relapsed B-cell non-Hodgkin's lymphoma Annals of Hematology 2006;85:769-779 [Non R free arm]
324.	Forstpointner,R. et al.Maintenance therapy with rituximab leads to a significant prolongation of response duration after salvage therapy with a combination of rituximab, fludarabine, cyclophosphamide, and mitoxantrone (R-FCM) in patients with recurring and refractory follicular Blood 2006;108:4003-4008 [Purging, maintenance and sequential protocols]
325.	Micallef,I. N. et al.A pilot study of epratuzumab and rituximab in combination with cyclophosphamide, doxorubicin, vincristine, and prednisone chemotherapy in patients with previously untreated, diffuse large B-cell lymphoma Cancer 2006;107:2826-2832 [Other MoAb included]
326.	Khan,K. D. et al.A phase 2 study of rituximab in combination with recombinant interleukin-2 for rituximab-refractory indolent non-Hodgkin's lymphoma Clinical cancer research  2006;12:7046-7053 [Non randomized studies]
327.	Provencio,M. et al.Rapid-infusion rituximab in lymphoma treatment Annals of Oncology  2006;17:1027-1028 [Abstract/editorial/review/comments]
328.	Tobinai,K. et al.Phase II study of oral fludarabine phosphate in relapsed indolent B-Cell non-Hodgkin's lymphoma Journal of clinical oncology  2006;24:174-180 [Non randomized studies]
329.	Rigacci,L. et al.Dose-dense CHOP plus rituximab (R-CHOP14) for the treatment of elderly patients with high-risk diffuse large B cell lymphoma: a pilot study Acta Haematologica 2006;115:22-27 [Non randomized studies]
330.	Ravandi,F. et al.Eradication of minimal residual disease in hairy cell leukemia Blood 2006;107:4658-4662 [Abstract/editorial/review/comments]
331.	Thomas,D. A. et al.Chemoimmunotherapy with hyper-CVAD plus rituximab for the treatment of adult Burkitt and Burkitt-type lymphoma or acute lymphoblastic leukemia Cancer 2006;106:1569-1580 [Non randomized studies]
332.	Niitsu,N. et al.Multicenter phase II study of the CyclOBEAP (CHOP-like + etoposide and bleomycin) regimen for patients with poor-prognosis aggressive lymphoma Annals of Hematology 2006;85:374-380 [Non R free arm]
333.	Brusamolino,E. et al.Dose-dense R-CHOP-14 supported by pegfilgrastim in patients with diffuse large B-cell lymphoma: a phase II study of feasibility and toxicity Haematologica 2006;91:496-502 [Non randomized studies]
334.	Neumann,F. et al.Rituximab long-term maintenance therapy after autologous stem cell transplantation in patients with B-cell non-Hodgkin's lymphoma Annals of Hematology 2006;85:530-534 [Non R free arm]
335.	Kahl,B. S. et al.Maintenance rituximab following induction chemoimmunotherapy may prolong progression-free survival in mantle cell lymphoma: a pilot study from the Wisconsin Oncology Network Annals of Oncology  2006;17:1418-1423 [Non randomized studies]
336.	Visco,C. et al.Distinctive natural history in hepatitis C virus positive diffuse large B-cell lymphoma: analysis of 156 patients from northern Italy Annals of Oncology  2006;17:1434-1440 [Non R free arm]
337.	Coso,D. et al.A phase II trial of rituximab as adjuvant to intensive sequential chemotherapy in patients under 60 years with untreated poor-prognosis diffuse large B-cell lymphoma Bone marrow transplantation 2006;38:217-222 [Non randomized studies]
338.	Niitsu,N. et al.Phase II study of the CPT-11, mitoxantrone and dexamethasone regimen in combination with rituximab in elderly patients with relapsed diffuse large B-cell lymphoma Cancer science 2006;97:933-937 [Non R free arm]
339.	Shikama,N. et al.A prospective study of reduced-dose three-course CHOP followed by involved-field radiotherapy for patients 70 years old or more with localized aggressive non-Hodgkin's lymphoma International journal of radiation oncology, biology, physics 2006;66:217-222 [Non R free arm]
340.	D'Arena,G. et al.Rituximab therapy for chronic lymphocytic leukemia-associated autoimmune hemolytic anemia American Journal of Hematology 2006;81:598-602 [Non R free arm]
341.	Ganjoo,K. N. et al.Rituximab, bevacizumab and CHOP (RA-CHOP) in untreated diffuse large B-cell lymphoma: safety, biomarker and pharmacokinetic analysis Leukemia & lymphoma 2006;47:998-1005 [Other MoAb included]
342.	Tomita,N. et al.Phase II study of CHOP-GR therapy for advanced-stage follicular lymphoma Leukemia & lymphoma 2006;47:1041-1047 [Non randomized studies]
343.	Strauss,S. J. et al.Multicenter phase II trial of immunotherapy with the humanized anti-CD22 antibody, epratuzumab, in combination with rituximab, in refractory or recurrent non-Hodgkin's lymphoma Journal of clinical oncology  2006;24:3880-3886 [Other MoAb included]
344.	Press,O. W. et al.Phase II trial of CHOP chemotherapy followed by tositumomab/iodine I-131 tositumomab for previously untreated follicular non-Hodgkin's lymphoma: five-year follow-up of Southwest Oncology Group Protocol S9911 Journal of clinical oncology  2006;24:4143-4149 [Other MoAb included]
345.	Boue,F. et al.Phase II trial of CHOP plus rituximab in patients with HIV-associated non-Hodgkin's lymphoma Journal of clinical oncology  2006;24:4123-4128 [Non randomized studies]
346.	Nishio,M. et al.Hypogammaglobulinemia with a selective delayed recovery in memory B cells and an impaired isotype expression after rituximab administration as an adjuvant to autologous stem cell transplantation for non-Hodgkin lymphoma European journal of haematology 2006;77:226-232 [Non R free arm]
347.	Intragumtornchai,T. et al.Rituximab-CHOP-ESHAP vs CHOP-ESHAP-high-dose therapy vs conventional CHOP chemotherapy in high-intermediate and high-risk aggressive non-Hodgkin's lymphoma Leukemia & lymphoma 2006;47:1306-1314 [Non randomized studies]
348.	Han,L. N. et al.Feasibility and efficacy of high-dose melphalan, cyclophosphamide, etoposide, and dexamethasone (LEED) chemotherapy with or without rituximab followed by autologous stem cell transplantation for aggressive and relapsed non-Hodgkin's lymphoma International journal of hematology 2006;84:174-181 [Non randomized studies]
349.	Leahy,M. F. et al.Multicenter phase II clinical study of iodine-131-rituximab radioimmunotherapy in relapsed or refractory indolent non-Hodgkin's lymphoma Journal of clinical oncology  2006;24:4418-4425 [Other MoAb included]
350.	Robak,T. et al.Rituximab combined with cladribine or with cladribine and cyclophosphamide in heavily pretreated patients with indolent lymphoproliferative disorders and mantle cell lymphoma Cancer 2006;107:1542-1550 [Non R free arm]
351.	Tinhofer,I. et al.The effect of IgVH mutational status on the induction of apoptosis by rituximab in patients with heavily pretreated B-cell chronic lymphocytic leukemia: evidence from a clinical phase I/II trial Haematologica 2006;91:1291-1293 [Non randomized studies]
352.	Oyan,B. et al.High dose sequential chemotherapy and autologous stem cell transplantation in patients with relapsed/refractory lymphoma Leukemia & lymphoma 2006;47:1545-1552 [Non R free arm]
353.	Mey,U. J. et al.Dexamethasone, high-dose cytarabine, and cisplatin in combination with rituximab as salvage treatment for patients with relapsed or refractory aggressive non-Hodgkin's lymphoma Cancer investigation 2006;24:593-600 [Non R free arm]
354.	Roberts,J. D. et al.Phase I study of bryostatin 1 and fludarabine in patients with chronic lymphocytic leukemia and indolent (non-Hodgkin's) lymphoma Clinical cancer research  2006;12:5809-5816 [Non randomized studies]
355.	Holmberg,L. A. et al.Immunotherapy with rituximab/interleukin-2 after autologous stem cell transplantation as treatment for CD20+ non-Hodgkin's lymphoma Clinical lymphoma & myeloma 2006;7:135-139 [Non randomized studies]
356.	Czuczman,M. S. et al.Prognostic factors for non-Hodgkin's lymphoma patients treated with chemotherapy may not predict outcome in patients treated with rituximab Leukemia & lymphoma 2006;47:1830-1840 [Non R free arm]
357.	Zaja,F. et al.CHOP-rituximab with pegylated liposomal doxorubicin for the treatment of elderly patients with diffuse large B-cell lymphoma Leukemia & lymphoma 2006;47:2174-2180 [Non randomized studies]
358.	Chanan-Khan,A. et al.Clinical efficacy of lenalidomide in patients with relapsed or refractory chronic lymphocytic leukemia: results of a phase II study Journal of clinical oncology  2006;24:5343-5349 [Non R free arm]
359.	Mey,U. J. et al.DHAP in combination with rituximab vs DHAP alone as salvage treatment for patients with relapsed or refractory diffuse large B-cell lymphoma: a matched-pair analysis Leukemia & lymphoma 2006;47:2558-2566 [Non randomized studies]
360.	Savage,K. J. et al.Favorable outcome of primary mediastinal large B-cell lymphoma in a single institution: The British Columbia experience. Annals of Oncology 2006;17:123-130 [Non R free arm]
361.	Zojer,N. et al.Rituximab treatment provides no clinical benefit in patients with pretreated advanced multiple myeloma Leukemia & lymphoma 2006;47:1103-1109 [Non randomized studies]
362.	Nabhan,C. et al.Minimal residual disease in chronic lymphocytic leukaemia: Is it ready for primetime?. British journal of haematology 2007;136:379-392 [Abstract/editorial/review/comments]
363.	Kasperkiewicz,M. et al.Rituximab (anti-CD20) for the treatment of autoimmune bullous diseases. Hautarzt 2007;58:115-121 [Non lymphoma ]
364.	Cheung,M. C. et al.Rituximab in lymphoma: A systematic review and consensus practice guideline from Cancer Care Ontario. Cancer treatment reviews 2007;33:161-176 [Abstract/editorial/review/comments]
365.	Sehn,L. H. et al.The revised International Prognostic Index (R-IPI) is a better predictor of outcome than the standard IPI for patients with diffuse large B-cell lymphoma treated with R-CHOP. Blood 2007;109:1857-1861 [Abstract/editorial/review/comments]
366.	Rummel,M. J. et al.Rituximab for the therapy of non-Hodgkin's lymphoma. Onkologe 2007;13:227-235 [Abstract/editorial/review/comments]
367.	Lee,S. -T et al.Therapeutic vaccine for lymphoma. Yonsei medical journal 2007;48:1-10 [Abstract/editorial/review/comments]
368.	Sokol,K. C. et al.Polypharmacy in older oncology patients and the need for an interdisciplinary approach to side-effect management. Journal of clinical pharmacy and therapeutics 2007;32:169-175 [Abstract/editorial/review/comments]
369.	Khashab,M. et al.Epidemiology of acute liver failure. Current gastroenterology reports 2007;9:66-73 [Non lymphoma ]
370.	Rodriguez,J. et al.New treatment concepts in diffuse large B-cell lymphomas (DLBL): Chemotherapy and biological therapy. Reviews on Recent Clinical Trials 2007;2:149-162 [Abstract/editorial/review/comments]
371.	Kato,K. et al.Reduced-intensity stem cell transplantation for hematological malignancies: Current status and the future. Current Stem Cell Research and Therapy 2007;2:149-162 [Abstract/editorial/review/comments]
372.	Alinari,L. et al.Alemtuzumab (Campath-1H) in the treatment of chronic lymphocytic leukemia. Oncogene 2007;26:3644-3653 [Other MoAb included]
373.	Coiffier,B. et al.Rituximab therapy in malignant lymphoma. Oncogene 2007;26:3603-3613 [Abstract/editorial/review/comments]
374.	Winter,J. N. et al.Defining the role of immunotherapy and radioimmunotherapy in the treatment of low-grade lymphoma. Current opinion in hematology 2007;14:360-368 [Abstract/editorial/review/comments]
375.	Bertele,V. et al.Haematological anticancer drugs in Europe: Any added value at the time of approval?. European journal of clinical pharmacology 2007;63:713-719 [Abstract/editorial/review/comments]
376.	Saven,A. et al.Randomized, double-blind, phase II study evaluating same-day vs next-day administration of pegfilgrastim with R-CHOP in non-Hodgkin's lymphoma patients. Journal of Supportive Oncology 2007;5:42-43 [Non R free arm]
377.	Ng,A. K. et al.Diffuse Large B-Cell Lymphoma. Seminars in radiation oncology 2007;17:169-175 [Abstract/editorial/review/comments]
378.	Smith,S. et al.Iodine 131 tositumomab in the treatment of non-Hodgkins's lymphoma. Future Oncology 2007;3:255-262 [Other MoAb included]
379.	Turturro,F. et al.Update on front-line therapy for follicular lymphoma: Chemo-immunotherapy with rituximab and survival. Expert Review of Anticancer Therapy 2007;7:959-965 [Abstract/editorial/review/comments]
380.	Fraser,G. et al.Alemtuzumab in chronic lymphocytic leukemia. Current Oncology 2007;14:96-109 [Other MoAb included]
381.	Ponzoni,M. et al.Definition, diagnosis, and management of intravascular large B-cell lymphoma: Proposals and perspectives from an international consensus meeting. Journal of Clinical Oncology 2007;25:3168-3173 [Abstract/editorial/review/comments]
382.	Gine,E. et al.The role of stem-cell transplantation in chronic lympocytic leukemia risk-adapted therapy. Best Practice and Research in Clinical Haematology 2007;20:529-543 [Abstract/editorial/review/comments]
383.	Fernandez,H. F. et al.Autotransplant conditioning regimens for aggressive lymphoma: Are we on the right road?. Bone marrow transplantation 2007;40:505-513 [Abstract/editorial/review/comments]
384.	Anonymous et al.Oblimersen: Augmerosen, BCL-2 antisense oligonucleotide - Genta, G 3139, GC 3139, oblimersen sodium. Drugs in R and D 2007;8:321-334 [Abstract/editorial/review/comments]
385.	Hayama,M. et al.t(6;14)(q15;q32) in a patient with CD5+CD10+ diffuse large B-cell lymphoma. International journal of hematology 2007;86:147-149 [Abstract/editorial/review/comments]
386.	Park,S. I. et al.Radioimmunotherapy for treatment of B-cell lymphomas and other hematologic malignancies. Current opinion in hematology 2007;14:632-638 [Abstract/editorial/review/comments]
387.	Zhang,Q. et al.Biweekly CHOP therapy improves therapeutic effect in the non-GCB subtype of diffuse large B-cell lymphoma. Central European Journal of Medicine 2007;2:488-498 [Non R free arm]
388.	Schmitz,N. et al.Autologous Stem Cell Transplantation in Lymphoma. Seminars in hematology 2007;44:234-245 [Abstract/editorial/review/comments]
389.	Dearden,C. E. et al.Role of antibody therapy in lymphoid malignancies. British medical bulletin 2007;83:275-290 [Abstract/editorial/review/comments]
390.	Mihelic,R. et al.Maintenance therapy in lymphoma. Clinical Lymphoma and Myeloma 2007;7:507-513 [Abstract/editorial/review/comments]
391.	Robak,T. et al.Recent progress in the management of chronic lymphocytic leukemia. Cancer treatment reviews 2007;33:710-728 [Abstract/editorial/review/comments]
392.	Shimoni,A. et al.Radioimmunotherapy and stem-cell transplantation in the treatment of aggressive B-cell lymphoma. Leukemia and Lymphoma 2007;48:2110-2120 [Abstract/editorial/review/comments]
393.	Sibilia,J. et al.Safety of T-cell co-stimulation modulation with abatacept in patients with rheumatoid arthritis. Clinical and experimental rheumatology 2007;25:46s-56s [Non lymphoma ]
394.	Meijer,J. M. et al.The future of biologic agents in the treatment of Sjogren's syndrome. Clinical Reviews in Allergy and Immunology 2007;32:292-297 [Abstract/editorial/review/comments]
395.	Matutes,E. et al.Splenic marginal zone lymphoma with and without villous lymphocytes. Current Treatment Options in Oncology 2007;8:109-116 [Abstract/editorial/review/comments]
396.	Schulz,H. et al.Chemotherapy plus Rituximab versus chemotherapy alone for B-cell non-Hodgkin's lymphoma. Cochrane Database of Systematic Reviews 2007;4:CD003805 [Abstract/editorial/review/comments]
397.	Foa,R. et al.Combination therapy with alemtuzumab: Existing data and future studies. Haematologica Meeting Reports 2007;1:25-32 [Abstract/editorial/review/comments]
398.	Bentz,M. et al.Where does 90Y-ibritumomab tiuxetan radioimmunotherapy fit? Selecting the right patient. Haematologica Meeting Reports 2007;1:61-68 [Abstract/editorial/review/comments]
399.	Tobinai,K. et al.4. Antibody therapy for malignant lymphoma Internal medicine (Tokyo, Japan) 2007;46:99-100 [Other MoAb included]
400.	Nehring,A. K. et al.Epstein-Barr virus T-cell immunity despite rituximab British journal of haematology 2007;136:628-632 [Non R free arm]
401.	Sieniawski,M. et al.Rituximab added to an intensified salvage chemotherapy program followed by autologous stem cell transplantation improved the outcome in relapsed and refractory aggressive non-Hodgkin lymphoma Annals of Hematology 2007;86:107-115 [Non randomized studies]
402.	Plonquet,A. et al.Peripheral blood natural killer cell count is associated with clinical outcome in patients with aaIPI 2-3 diffuse large B-cell lymphoma Annals of Oncology  2007;18:1209-1215 [Non R free arm]
403.	Kim,M. K. et al.A randomized comparison of peripheral blood hematopoietic progenitor cell level of 5/mm3 versus 50/mm3 as a surrogate marker to initiate efficient autologous blood stem cell collection Journal of clinical apheresis 2007;22:277-282 [Non R free arm]
404.	Economopoulos,T. et al.CEOP-21 versus CEOP-14 chemotherapy with or without rituximab for the first-line treatment of patients with aggressive lymphomas: results of the HE22A99 trial of the Hellenic Cooperative Oncology Group Cancer journal (Sudbury, Mass.) 2007;13:327-334 [Non R free arm]
405.	Ritchie,D. S. et al.The hyper-CVAD-rituximab chemotherapy programme followed by high-dose busulfan, melphalan and autologous stem cell transplantation produces excellent event-free survival in patients with previously untreated mantle cell lymphoma Annals of Hematology 2007;86:101-105 [Non randomized studies]
406.	Leonard,J. P. et al.A phase I/II study of galiximab (an anti-CD80 monoclonal antibody) in combination with rituximab for relapsed or refractory, follicular lymphoma Annals of Oncology  2007;18:1216-1223 [Other MoAb included]
407.	Rigacci,L. et al.Rituximab and chlorambucil as first-line treatment for low-grade ocular adnexal lymphomas Annals of Hematology 2007;86:565-568 [Non randomized studies]
408.	de Latour,R. P. et al.Mobilization of peripheral blood progenitor cells after DHAP regimen with or without rituximab: a large multicenter comparative study in patients with malignant lymphoma Leukemia & lymphoma 2007;48:897-904 [Purging, maintenance and sequential protocols]
409.	Yamanaka,R. et al.Immuno-chemotherapy with a combination of rituximab, methotrexate, pirarubicin and procarbazine for patients with primary CNS lymphoma--a preliminary report Leukemia & lymphoma 2007;48:1019-1022 [Abstract/editorial/review/comments]
410.	Rigacci,L. et al.Liposome-encapsulated doxorubicin in combination with cyclophosphamide, vincristine, prednisone and rituximab in patients with lymphoma and concurrent cardiac diseases or pre-treated with anthracyclines Hematological oncology 2007;25:198-203 [Non randomized studies]
411.	Browning,R. L. et al.Expression of TCL-1 as a potential prognostic factor for treatment outcome in B-cell chronic lymphocytic leukemia Leukemia research 2007;31:1737-1740 [Non randomized studies]
412.	Seror,R. et al.Tolerance and efficacy of rituximab and changes in serum B cell biomarkers in patients with systemic complications of primary Sjogren's syndrome Annals of the Rheumatic Diseases 2007;66:351-357 [Non lymphoma ]
413.	Kay,N. E. et al.Combination chemoimmunotherapy with pentostatin, cyclophosphamide, and rituximab shows significant clinical activity with low accompanying toxicity in previously untreated B chronic lymphocytic leukemia Blood 2007;109:405-411 [Non R free arm]
414.	Aydin,S. et al.Rituximab plus ASHAP for the treatment of patients with relapsed or refractory aggressive non-Hodgkin's lymphoma: a single-centre study of 20 patients Annals of Hematology 2007;86:271-276 [Non randomized studies]
415.	Dreger,P. et al.Rituximab-augmented myeloablation for first-line autologous stem cell transplantation for mantle cell lymphoma: effects on molecular response and clinical outcome Haematologica 2007;92:42-49 [Non randomized studies]
416.	Sehn,L. H. et al.Rapid infusion rituximab in combination with corticosteroid-containing chemotherapy or as maintenance therapy is well tolerated and can safely be delivered in the community setting Blood 2007;109:4171-4173 [Non R free arm]
417.	Rubenstein,J. L. et al.Phase I study of intraventricular administration of rituximab in patients with recurrent CNS and intraocular lymphoma Journal of clinical oncology  2007;25:1350-1356 [Non randomized studies]
418.	Schutt,P. et al.Ifosfamide, etoposide, cytarabine, and dexamethasone as salvage treatment followed by high-dose cyclophosphamide, melphalan, and etoposide with autologous peripheral blood stem cell transplantation for relapsed or refractory lymphomas European journal of haematology 2007;78:93-101 [Non R free arm]
419.	Todisco,E. et al.CD34+ dose-driven administration of granulocyte colony-stimulating factor after high-dose chemotherapy in lymphoma patients European journal of haematology 2007;78:111-116 [Non R free arm]
420.	Kurkus,J. et al.Biocompatibility of a novel avidin-agarose adsorbent for extracorporeal removal of redundant radiopharmaceutical from the blood Artificial Organs 2007;31:208-214 [Non R free arm]
421.	Kamezaki,K. et al.Rituximab does not compromise the mobilization and engraftment of autologous peripheral blood stem cells in diffuse-large B-cell lymphoma Bone marrow transplantation 2007;39:523-527 [Non randomized studies]
422.	Shimoni,A. et al.Yttrium-90-ibritumomab tiuxetan (Zevalin) combined with high-dose BEAM chemotherapy and autologous stem cell transplantation for chemo-refractory aggressive non-Hodgkin's lymphoma Experimental hematology 2007;35:534-540 [Other MoAb included]
423.	Morschhauser,F. et al.Efficacy and safety of yttrium-90 ibritumomab tiuxetan in patients with relapsed or refractory diffuse large B-cell lymphoma not appropriate for autologous stem-cell transplantation Blood 2007;110:54-58 [Other MoAb included]
424.	Sacchi,S. et al.Introduction of rituximab in front-line and salvage therapies has improved outcome of advanced-stage follicular lymphoma patients Cancer 2007;109:2077-2082 [Non randomized studies]
425.	Jager,G. et al.CHOP chemotherapy followed by Rituximab consolidation as first line treatment in patients with follicular lymphoma. Long-term follow-up of a phase 2 trial European journal of haematology 2007;78:453-455 [Purging, maintenance and sequential protocols]
426.	Khouri,I. F. et al.Graft-versus-leukaemia effect after non-myeloablative haematopoietic transplantation can overcome the unfavourable expression of ZAP-70 in refractory chronic lymphocytic leukaemia British journal of haematology 2007;137:355-363 [Non randomized studies]
427.	Tsurumi,H. et al.A phase II study of a THP-COP regimen for the treatment of elderly patients aged 70 years or older with diffuse large B-cell lymphoma Hematological oncology 2007;25:107-114 [Non randomized studies]
428.	El Gnaoui,T. et al.Rituximab, gemcitabine and oxaliplatin: an effective salvage regimen for patients with relapsed or refractory B-cell lymphoma not candidates for high-dose therapy Annals of Oncology  2007;18:1363-1368 [Non randomized studies]
429.	Sacchi,S. et al.Rituximab in combination with fludarabine and cyclophosphamide in the treatment of patients with recurrent follicular lymphoma Cancer 2007;110:121-128 [Non randomized studies]
430.	Tarella,C. et al.Prolonged survival in poor-risk diffuse large B-cell lymphoma following front-line treatment with rituximab-supplemented, early-intensified chemotherapy with multiple autologous hematopoietic stem cell support: a multicenter study by GITIL (Gruppo Italian Leukemia  2007;21:1802-1811 [Non R free arm]
431.	Case,D. C.,Jr et al.Community-based trial of R-CHOP and maintenance rituximab for intermediate- or high-grade non-Hodgkin lymphoma with first-cycle filgrastim for older patients Clinical lymphoma & myeloma 2007;7:354-360 [Non randomized studies]
432.	Yano,S. et al.Long-term follow-up of autologous stem cell transplantation for patients with aggressive non-Hodgkin lymphoma who had bone marrow involvement at initial diagnosis in the pre-rituximab era Clinical lymphoma & myeloma 2007;7:361-363 [Non R free arm]
433.	Li,J. M. et al.Rituximab in combination with CHOP chemotherapy for the treatment of diffuse large B cell lymphoma in Chinese patients Annals of Hematology 2007;86:639-645 [Non randomized studies]
434.	Galimberti,S. et al.The efficacy of rituximab plus Hyper-CVAD regimen in mantle cell lymphoma is independent of FCgammaRIIIa and FCgammaRIIa polymorphisms Journal of chemotherapy (Florence, Italy) 2007;19:315-321 [Non randomized studies]
435.	Dillman,R. O. et al.Community-based phase II trial of pentostatin, cyclophosphamide, and rituximab (PCR) biochemotherapy in chronic lymphocytic leukemia and small lymphocytic lymphoma Cancer biotherapy & radiopharmaceuticals 2007;22:185-193 [Non randomized studies]
436.	Dang,N. H. et al.Phase II trial of the combination of denileukin diftitox and rituximab for relapsed/refractory B-cell non-Hodgkin lymphoma British journal of haematology 2007;138:502-505 [Non randomized studies]
437.	Weide,R. et al.High anti-lymphoma activity of bendamustine/mitoxantrone/rituximab in rituximab pretreated relapsed or refractory indolent lymphomas and mantle cell lymphomas. A multicenter phase II study of the German Low Grade Lymphoma Study Group (GLSG) Leukemia & lymphoma 2007;48:1299-1306 [Non randomized studies]
438.	Yamanaka,R. et al.Salvage immuno-chemotherapy with a combination of rituximab, high-dose cytarabine, mitoxantrone and dexamethasone for patients with primary CNS lymphoma: a preliminary study Leukemia & lymphoma 2007;48:1429-1433 [Abstract/editorial/review/comments]
439.	Harting,R. et al.Efficacy and safety of rituximab combined with ESHAP chemotherapy for the treatment of relapsed/refractory aggressive B-cell non-Hodgkin lymphoma Clinical lymphoma & myeloma 2007;7:406-412 [Non randomized studies]
440.	Robak,T. et al.Rituximab plus cladribine with or without cyclophosphamide in patients with relapsed or refractory chronic lymphocytic leukemia European journal of haematology 2007;79:107-113 [Non R free arm]
441.	Carlotti,E. et al.FcgammaRIIIA and FcgammaRIIA polymorphisms do not predict clinical outcome of follicular non-Hodgkin's lymphoma patients treated with sequential CHOP and rituximab Haematologica 2007;92:1127-1130 [Purging, maintenance and sequential protocols]
442.	Aviles,A. et al.Rituximab and dose dense chemotherapy in primary breast lymphoma Haematologica 2007;92:1147-1148 [Non randomized studies]
443.	Galimberti,S. et al.Evaluation of the MDR1, ABCG2, Topoisomerases IIalpha and GSTpi gene expression in patients affected by aggressive mantle cell lymphoma treated by the R-Hyper-CVAD regimen Leukemia & lymphoma 2007;48:1502-1509 [Non randomized studies]
444.	Kane,R. C. et al.Bortezomib for the treatment of mantle cell lymphoma Clinical cancer research  2007;13:5291-5294 [Non R free arm]
445.	Cooney-Qualter,E. et al.A phase I study of 90yttrium-ibritumomab-tiuxetan in children and adolescents with relapsed/refractory CD20-positive non-Hodgkin's lymphoma: a Children's Oncology Group study Clinical cancer research  2007;13:5652s-5660s [Other MoAb included]
446.	Grange,F. et al.Primary cutaneous diffuse large B-cell lymphoma, leg type: clinicopathologic features and prognostic analysis in 60 cases Archives of Dermatology 2007;143:1144-1150 [Non R free arm]
447.	Kuzel,T. M. et al.Phase II study of denileukin diftitox for previously treated indolent non-Hodgkin lymphoma: final results of E1497 Leukemia & lymphoma 2007;48:2397-2402 [Non randomized studies]
448.	Leonard,J. P. et al.Phase I trial of toll-like receptor 9 agonist PF-3512676 with and following rituximab in patients with recurrent indolent and aggressive non Hodgkin's lymphoma Clinical cancer research  2007;13:6168-6174 [Non randomized studies]
449.	Shah,G. D. et al.Combined immunochemotherapy with reduced whole-brain radiotherapy for newly diagnosed primary CNS lymphoma Journal of clinical oncology  2007;25:4730-4735 [Non randomized studies]
450.	Fina,M. et al.VNCOP-B plus rituximab in the treatment of diffuse large B-cell lymphoma in the elderly Leukemia & lymphoma 2007;48:2167-2171 [Non randomized studies]
451.	Rodriguez,J. et al.Rituximab, gemcitabine and oxaliplatin: an effective regimen in patients with refractory and relapsing mantle cell lymphoma Leukemia & lymphoma 2007;48:2172-2178 [Non randomized studies]
452.	Jurczak,W. et al.Radioimmunotherapy in follicular lymphomas, a retrospective analysis of the Polish Lymphoma Research Group's (PLRG) experience Nuclear medicine review.Central & Eastern Europe  2007;10:91-97 [Other MoAb included]
453.	Moreau,P. et al.Rituximab in CD20 positive multiple myeloma Leukemia  2007;21:835-836 [Abstract/editorial/review/comments]
454.	Baz,R. et al.Combination of rituximab and oral melphalan and prednisone in newly diagnosed multiple myeloma Leukemia & lymphoma 2007;48:2338-2344 [Non randomized studies]
455.	Dimopoulos,M. A. et al.Primary treatment of Waldenstrom macroglobulinemia with dexamethasone, rituximab, and cyclophosphamide Journal of clinical oncology  2007;25:3344-3349 [Non randomized studies]
456.	Lavori,P. W. et al.Adaptive treatment strategies in chronic disease. Annual Review of Medicine 2008;59:443-453 [Abstract/editorial/review/comments]
457.	Molina,A. et al.A decade of rituximab: Improving survival outcomes in non-Hodgkin's lymphoma. Annual Review of Medicine 2008;59:237-250 [Abstract/editorial/review/comments]
458.	Nabhan,C. et al.Controversies in the front-line management of chronic lymphocytic leukemia. Leukemia research 2008;32:679-688 [Abstract/editorial/review/comments]
459.	Burt,R. K. et al.Clinical applications of blood-derived and marrow-derived stem cells for nonmalignant diseases. JAMA - Journal of the American Medical Association 2008;299:925-936 [Abstract/editorial/review/comments]
460.	Jahrsdorfer,B. et al.CpG oligodeoxynucleotides as immunotherapy in cancer. Update on Cancer Therapeutics 2008;3:27-32 [Abstract/editorial/review/comments]
461.	Zhang,M. M. et al.Radioimmunotherapy-Based Conditioning Regimens for Stem Cell Transplantation. Seminars in hematology 2008;45:118-125 [Other MoAb included]
462.	O'Brien,S. et al.Valganciclovir prevents cytomegalovirus reactivation in patients receiving alemtuzumab-based therapy. Blood 2008;111:1816-1819 [Other MoAb included]
463.	Dearden,C. et al.The prognostic significance of a positive direct antiglobulin test in chronic lymphocytic leukemia: A beneficial effect of the combination of fludarabine and cyclophosphamide on the incidence of hemolytic anemia. Blood 2008;111:1820-1826 [Non R free arm]
464.	Janssens,A. et al.Monoclonal antibodies in the treatment of lymphoid malignancies. Tijdschrift voor Geneeskunde 2008;64:3610366- [Abstract/editorial/review/comments]
465.	Greb,A. et al.High-dose chemotherapy with autologous stem cell transplantation in the first line treatment of aggressive Non-Hodgkin Lymphoma (NHL) in adults. Cochrane Database of Systematic Reviews 2008;1:CD004024 [Abstract/editorial/review/comments]
466.	Robak,T. et al.Novel drugs in chronic lymphocytic leukemia. Acta Haematologica Polonica 2008;39:179-189 [Abstract/editorial/review/comments]
467.	Iorizzo III,L. J. et al.The treatment and prognosis of dermatomyositis: An updated review. Journal of the American Academy of Dermatology 2008;59:99-112 [Abstract/editorial/review/comments]
468.	Ennishi,D. et al.Increased incidence of interstitial pneumonia by CHOP combined with rituximab. International journal of hematology 2008;87:393-397 [Non randomized studies]
469.	Ding,C. et al.B-cell-targeted therapy for systemic lupus erythematosus: An update. BioDrugs 2008;22:239-249 [Abstract/editorial/review/comments]
470.	Park,H. J. et al.Developing idiotype vaccines for lymphoma: From preclinical studies to phase III clinical trials. British journal of haematology 2008;142:179-191 [Abstract/editorial/review/comments]
471.	Safdar,A. et al.Multiple-dose granulocyte-macrophage-colony-stimulating factor plus 23-valent polysaccharide pneumococcal vaccine in patients with chronic lymphocytic leukemia: A prospective, randomized trial of safety and immunogenicity. Cancer 2008;113:383-387 [Non R free arm]
472.	Bendandi,M. et al.Aiming at a curative strategy for follicular lymphoma. CA Cancer Journal for Clinicians 2008;58:305-317 [Abstract/editorial/review/comments]
473.	Westhovens,R. et al.Translating co-stimulation blockade into clinical practice. Arthritis Research and Therapy 2008;10 (s1):not reported [Abstract/editorial/review/comments]
474.	Robak,T. et al.Alemtuzumab for B-cell chronic lymphocytic leukemia. Expert Review of Anticancer Therapy 2008;8:1033-1051 [Other MoAb included]
475.	Isaksen,K. et al.Anti-CD20 treatment in primary Sjogren's syndrome. Scandinavian journal of immunology 2008;68:554-564 [Abstract/editorial/review/comments]
476.	Nagai,H. et al.Remission induction therapy containing rituximab markedly improved the outcome of untreated mature B cell lymphoma. British journal of haematology 2008;143:672-680 [Non randomized studies]
477.	Wiendl,H. et al.Basic and escalating immunomodulatory treatments in multiple sclerosis: Current therapeutic recommendations. Journal of neurology 2008;255:1449-1463 [Abstract/editorial/review/comments]
478.	Ghesquieres,H. et al.Combination of rituximab with chemotherapy in diffuse large B-cell lymphoma. Evaluation in daily practice before and after approval of rituximab in this indication. Hematological oncology 2008;26:139-147 [Non randomized studies]
479.	Coca,A. et al.Targeted biologic approaches to the treatment of systemic vasculitis. Clinical Reviews in Allergy and Immunology 2008;35:79-87 [Abstract/editorial/review/comments]
480.	Lugtenburg,P. J. et al.Treatment of diffuse large B-cell lymphoma in the elderly: Strategies integrating oncogeriatric themes. Current oncology reports 2008;10:412-419 [Abstract/editorial/review/comments]
481.	Sikder,M. A. et al.Beyond rituximab: The future of monoclonal antibodies in B-cell non-Hodgkin lymphoma. Current oncology reports 2008;10:420-426 [Abstract/editorial/review/comments]
482.	Sikder,M. A. et al.Beyond rituximab: The future of monoclonal antibodies in B-cell non-Hodgkin lymphoma. Current Hematologic Malignancy Reports 2008;3:187-193 [Abstract/editorial/review/comments]
483.	Senff,N. J. et al.European Organization for Research and Treatment of Cancer and International Society for cutaneous lymphoma Consensus recommendations for the management of cutaneous B-cell lymphomas. Blood 2008;112:1600-1609 [Abstract/editorial/review/comments]
484.	Robak,T. et al.New therapies for patients with chronic lymphocytic leukemia. Current Cancer Therapy Reviews 2008;4:235-242 [Abstract/editorial/review/comments]
485.	Gross,T. G. et al.Where there are no randomized, phase 3 trials-what is a doctor to do?. Journal of Pediatric Hematology/Oncology 2008;30:719-720 [Abstract/editorial/review/comments]
486.	Canioni,D. et al.High numbers of tumor-associated macrophages have an adverse prognostic value that can be circumvented by rituximab in patients with follicular lymphoma enrolled onto the GELA-GOELAMS FL-2000 trial Journal of clinical oncology  2008;26:440-446 [Non R free arm]
487.	Kimby,E. et al.Long-term molecular remissions in patients with indolent lymphoma treated with rituximab as a single agent or in combination with interferon alpha-2a: a randomized phase II study from the Nordic Lymphoma Group Leukemia & lymphoma 2008;49:102-112 [Non randomized studies]
488.	Hornberger,J. et al.Economic evaluation of rituximab plus cyclophosphamide, vincristine and prednisolone for advanced follicular lymphoma Leukemia & lymphoma 2008;49:227-236 [No infection outcome]
489.	Ladetto,M. et al.Prospective, multicenter randomized GITMO/IIL trial comparing intensive (R-HDS) versus conventional (CHOP-R) chemoimmunotherapy in high-risk follicular lymphoma at diagnosis: the superior disease control of R-HDS does not translate into an overall surviva Blood 2008;111:4004-4013 [Non R free arm]
490.	Pfreundschuh,M. et al.Prognostic significance of maximum tumour (bulk) diameter in young patients with good-prognosis diffuse large-B-cell lymphoma treated with CHOP-like chemotherapy with or without rituximab: an exploratory analysis of the MabThera International Trial Group The lancet oncology 2008;9:435-444 [Duplicate publication]
491.	Dungarwalla,M. et al.High dose methylprednisolone and rituximab is an effective therapy in advanced refractory chronic lymphocytic leukemia resistant to fludarabine therapy Haematologica 2008;93:475-476 [Non randomized studies]
492.	Hirt,C. et al.Rapid and sustained clearance of circulating lymphoma cells after chemotherapy plus rituximab: clinical significance of quantitative t(14;18) PCR monitoring in advanced stage follicular lymphoma patients British journal of haematology 2008;141:631-640 [No infection outcome]
493.	Zwick,C. et al.Equitoxicity of bolus and infusional etoposide: results of a multicenter randomised trial of the German High-Grade Non-Hodgkins Lymphoma Study Group (DSHNHL) in elderly patients with refractory or relapsing aggressive non-Hodgkin lymphoma using the CEMP r Annals of Hematology 2008;87:717-726 [Non R free arm]
494.	Kasteng,F. et al.Cost-effectiveness of maintenance rituximab treatment after second line therapy in patients with follicular lymphoma in Sweden Acta Oncologica (Stockholm, Sweden) 2008;47:1029-1036 [Non randomized studies]
495.	Marcus,R. et al.Phase III study of R-CVP compared with cyclophosphamide, vincristine, and prednisone alone in patients with previously untreated advanced follicular lymphoma Journal of clinical oncology  2008;26:4579-4586 [Duplicate publication]
496.	Hashino,S. et al.Cost benefit and clinical efficacy of low-dose granulocyte colony-stimulating factor after standard chemotherapy in patients with non-Hodgkin's lymphoma International journal of laboratory hematology 2008;30:292-299 [Non R free arm]
497.	Hamlin,P. A. et al.Do six or eight cycles work better with CHOP-14 and rituximab? Current oncology reports 2008;10:391-392 [Abstract/editorial/review/comments]
498.	Jacobs,S. A. et al.Phase II trial of short-course CHOP-R followed by 90Y-ibritumomab tiuxetan and extended rituximab in previously untreated follicular lymphoma Clinical cancer research  2008;14:7088-7094 [Other MoAb included]
499.	Salles,G. et al.Rituximab combined with chemotherapy and interferon in follicular lymphoma patients: results of the GELA-GOELAMS FL2000 study Blood 2008;112:4824-4831 [Non R free arm]
500.	Morschhauser,F. et al.Phase III trial of consolidation therapy with yttrium-90-ibritumomab tiuxetan compared with no additional therapy after first remission in advanced follicular lymphoma Journal of clinical oncology  2008;26:5156-5164 [Other MoAb included]
501.	Vellenga,E. et al.Rituximab improves the treatment results of DHAP-VIM-DHAP and ASCT in relapsed/progressive aggressive CD20+ NHL: a prospective randomized HOVON trial Blood 2008;111:537-543 [No infection outcome]
502.	Raynaud,P. et al.T-cell lymphoid aggregates in bone marrow after rituximab therapy for B-cell follicular lymphoma: a marker of therapeutic efficacy? Human pathology 2008;39:194-200 [Non R free arm]
503.	Pfreundschuh,M. et al.Dose-escalated CHOEP for the treatment of young patients with aggressive non-Hodgkin's lymphoma: II. Results of the randomized high-CHOEP trial of the German High-Grade Non-Hodgkin's Lymphoma Study Group (DSHNHL) Annals of Oncology  2008;19:545-552 [Non R free arm]
504.	Torres-Garcia,E. et al.Biokinetics and dosimetry of 188Re-anti-CD20 in patients with non-Hodgkin's lymphoma: preliminary experience Archives of Medical Research 2008;39:100-109 [Other MoAb included]
505.	Oki,Y. et al.Phase 2 study of gemcitabine in combination with rituximab in patients with recurrent or refractory Hodgkin lymphoma Cancer 2008;112:831-836 [Non randomized studies]
506.	Arcaini,L. et al.Immunochemotherapy with in vivo purging and autotransplant induces long clinical and molecular remission in advanced relapsed and refractory follicular lymphoma Annals of Oncology  2008;19:1331-1335 [Non randomized studies]
507.	Wilson,W. H. et al.Phase II study of dose-adjusted EPOCH and rituximab in untreated diffuse large B-cell lymphoma with analysis of germinal center and post-germinal center biomarkers Journal of clinical oncology  2008;26:2717-2724 [Non randomized studies]
508.	Hagenbeek,A. et al.First clinical use of ofatumumab, a novel fully human anti-CD20 monoclonal antibody in relapsed or refractory follicular lymphoma: results of a phase 1/2 trial Blood 2008;111:5486-5495 [Other MoAb included]
509.	Rueda,A. et al.R-CHOP-14 in patients with diffuse large B-cell lymphoma younger than 70 years: a multicentre, prospective study Hematological oncology 2008;26:27-32 [Non randomized studies]
510.	Morschhauser,F. et al.A phase II study of enzastaurin, a protein kinase C beta inhibitor, in patients with relapsed or refractory mantle cell lymphoma Annals of Oncology  2008;19:247-253 [Non randomized studies]
511.	Kang,H. J. et al.Irinotecan plus cisplatin and dexamethasone (ICD) combination chemotherapy for patients with diffuse large B-cell lymphoma previously treated with Rituximab plus CHOP Cancer chemotherapy and pharmacology 2008;62:299-304 [Non R free arm]
512.	Schulz,H. et al.Rituximab in relapsed lymphocyte-predominant Hodgkin lymphoma: long-term results of a phase 2 trial by the German Hodgkin Lymphoma Study Group (GHSG) Blood 2008;111:109-111 [Non randomized studies]
513.	Oki,Y. et al.Phase II study of a salvage regimen using cyclophosphamide, high-dose cytarabine, dexamethasone, etoposide, and rituximab in patients with relapsed or refractory B-cell non-Hodgkin's lymphoma Cancer science 2008;99:179-184 [Non randomized studies]
514.	Del Poeta,G. et al.Consolidation and maintenance immunotherapy with rituximab improve clinical outcome in patients with B-cell chronic lymphocytic leukemia Cancer 2008;112:119-128 [Non R free arm]
515.	Lopez,A. et al.GEMOX-R regimen is a highly effective salvage regimen in patients with refractory/relapsing diffuse large-cell lymphoma: a phase II study European journal of haematology 2008;80:127-132 [Non randomized studies]
516.	Natkunam,Y. et al.LMO2 protein expression predicts survival in patients with diffuse large B-cell lymphoma treated with anthracycline-based chemotherapy with and without rituximab Journal of clinical oncology  2008;26:447-454 [Non R free arm]
517.	Ribera,J. M. et al.Safety and efficacy of cyclophosphamide, adriamycin, vincristine, prednisone and rituximab in patients with human immunodeficiency virus-associated diffuse large B-cell lymphoma: results of a phase II trial British journal of haematology 2008;140:411-419 [Non randomized studies]
518.	Evens,A. M. et al.A phase II clinical trial of intensive chemotherapy followed by consolidative stem cell transplant: long-term follow-up in newly diagnosed mantle cell lymphoma British journal of haematology 2008;140:385-393 [Non randomized studies]
519.	Tsimberidou,A. M. et al.Phase I-II study of oxaliplatin, fludarabine, cytarabine, and rituximab combination therapy in patients with Richter's syndrome or fludarabine-refractory chronic lymphocytic leukemia Journal of clinical oncology  2008;26:196-203 [Non R free arm]
520.	Friedberg,J. W. et al.Bendamustine in patients with rituximab-refractory indolent and transformed non-Hodgkin's lymphoma: results from a phase II multicenter, single-agent study Journal of clinical oncology  2008;26:204-210 [Non randomized studies]
521.	Hainsworth,J. D. et al.Combination therapy with fludarabine and rituximab followed by alemtuzumab in the first-line treatment of patients with chronic lymphocytic leukemia or small lymphocytic lymphoma: a phase 2 trial of the Minnie Pearl Cancer Research Network Cancer 2008;112:1288-1295 [Other MoAb included]
522.	Economopoulos,T. et al.Phase II study of low-grade non-Hodgkin lymphomas with fludarabine and mitoxantrone followed by rituximab consolidation: promising results in marginal zone lymphoma Leukemia & lymphoma 2008;49:68-74 [Non randomized studies]
523.	Lim,S. H. et al.B-cell depletion for 2 years after autologous stem cell transplant for NHL induces prolonged hypogammaglobulinemia beyond the rituximab maintenance period Leukemia & lymphoma 2008;49:152-153 [Abstract/editorial/review/comments]
524.	Montoto,S. et al.High clinical and molecular response rates with fludarabine, cyclophosphamide and mitoxantrone in previously untreated patients with advanced stage follicular lymphoma Haematologica 2008;93:207-214 [Non randomized studies]
525.	de Romeuf,C. et al.Chronic lymphocytic leukaemia cells are efficiently killed by an anti-CD20 monoclonal antibody selected for improved engagement of FcgammaRIIIA/CD16 British journal of haematology 2008;140:635-643 [Non R free arm]
526.	Taskinen,M. et al.Prognostic influence of tumor-infiltrating mast cells in patients with follicular lymphoma treated with rituximab and CHOP Blood 2008;111:4664-4667 [Non randomized studies]
527.	Zinzani,P. L. et al.Fludarabine and mitoxantrone followed by yttrium-90 ibritumomab tiuxetan in previously untreated patients with follicular non-Hodgkin lymphoma trial: a phase II non-randomised trial (FLUMIZ) The lancet oncology 2008;9:352-358 [Other MoAb included]
528.	Tam,C. S. et al.Long-term results of the fludarabine, cyclophosphamide, and rituximab regimen as initial therapy of chronic lymphocytic leukemia Blood 2008;112:975-980 [Non randomized studies]
529.	Khouri,I. F. et al.Eight-year experience with allogeneic stem cell transplantation for relapsed follicular lymphoma after nonmyeloablative conditioning with fludarabine, cyclophosphamide, and rituximab Blood 2008;111:5530-5536 [Non randomized studies]
530.	Persky,D. O. et al.Phase II study of rituximab plus three cycles of CHOP and involved-field radiotherapy for patients with limited-stage aggressive B-cell lymphoma: Southwest Oncology Group study 0014 Journal of clinical oncology  2008;26:2258-2263 [Non randomized studies]
531.	Kilickap,S. et al.Addition of rituximab to chop does not increase the risk of cardiotoxicity in patients with non-Hodgkin's lymphoma Medical oncology  2008;25:437-442 [No infection outcome]
532.	Cartron,G. et al.Granulocyte-macrophage colony-stimulating factor potentiates rituximab in patients with relapsed follicular lymphoma: results of a phase II study Journal of clinical oncology  2008;26:2725-2731 [Non randomized studies]
533.	Bishton,M. J. et al.A prospective study of the separate predictive capabilities of 18[F]-FDG-PET and molecular response in patients with relapsed indolent non-Hodgkin's lymphoma following treatment with iodine-131-rituximab radio-immunotherapy Haematologica 2008;93:789-790 [Non randomized studies]
534.	Oriol,A. et al.High-dose chemotherapy and immunotherapy in adult Burkitt lymphoma: comparison of results in human immunodeficiency virus-infected and noninfected patients Cancer 2008;113:117-125 [Non randomized studies]
535.	Inwards,D. J. et al.Long-term results of the treatment of patients with mantle cell lymphoma with cladribine (2-CDA) alone (95-80-53) or 2-CDA and rituximab (N0189) in the North Central Cancer Treatment Group Cancer 2008;113:108-116 [Abstract/editorial/review/comments]
536.	Tarella,C. et al.Rituximab improves the efficacy of high-dose chemotherapy with autograft for high-risk follicular and diffuse large B-cell lymphoma: a multicenter Gruppo Italiano Terapie Innnovative nei linfomi survey Journal of clinical oncology  2008;26:3166-3175 [Non randomized studies]
537.	Shimada,K. et al.Retrospective analysis of intravascular large B-cell lymphoma treated with rituximab-containing chemotherapy as reported by the IVL study group in Japan Journal of clinical oncology  2008;26:3189-3195 [Non randomized studies]
538.	Witzig,T. E. et al.Salvage chemotherapy with rituximab DHAP for relapsed non-Hodgkin lymphoma: a phase II trial in the North Central Cancer Treatment Group Leukemia & lymphoma 2008;49:1074-1080 [Non randomized studies]
539.	Menzel,H. et al.Ifosfamide, epirubicin and etoposide rituximab in refractory or relapsed B-cell lymphoma: analysis of remission induction and stem cell mobilization Leukemia & lymphoma 2008;49:1337-1344 [Non randomized studies]
540.	Geisler,C. H. et al.Long-term progression-free survival of mantle cell lymphoma after intensive front-line immunochemotherapy with in vivo-purged stem cell rescue: a nonrandomized phase 2 multicenter study by the Nordic Lymphoma Group Blood 2008;112:2687-2693 [Non randomized studies]
541.	Robinson,K. S. et al.Phase II multicenter study of bendamustine plus rituximab in patients with relapsed indolent B-cell and mantle cell non-Hodgkin's lymphoma Journal of clinical oncology  2008;26:4473-4479 [Non randomized studies]
542.	Hayslip,J. W. et al.Cost-effectiveness of extended adjuvant rituximab for US patients aged 65-70 years with follicular lymphoma in second remission Clinical lymphoma & myeloma 2008;8:166-170 [Non R free arm]
543.	Klepfish,A. et al.Adding fresh frozen plasma to rituximab for the treatment of patients with refractory advanced CLL QJM  2008;101:737-740 [Non R free arm]
544.	Maza,S. et al.Yttrium-90 ibritumomab tiuxetan radioimmunotherapy in primary cutaneous B-cell lymphomas: first results of a prospective, monocentre study Leukemia & lymphoma 2008;49:1702-1709 [Other MoAb included]
545.	Damon,L. et al.Impact of intensive PBSC mobilization therapy on outcomes following auto-SCT for non-Hodgkin's lymphoma Bone marrow transplantation 2008;42:649-657 [Non R free arm]
546.	O'Connor,O. A. et al.A multicentre phase II clinical experience with the novel aza-epothilone Ixabepilone (BMS247550) in patients with relapsed or refractory indolent non-Hodgkin lymphoma and mantle cell lymphoma British journal of haematology 2008;143:201-209 [Non randomized studies]
547.	Ferreri,A. J. et al.The addition of rituximab to anthracycline-based chemotherapy significantly improves outcome in 'Western' patients with intravascular large B-cell lymphoma British journal of haematology 2008;143:253-257 [Non randomized studies]
548.	Lamanna,N. et al.Consolidation and maintenance rituximab therapy in chronic lymphocytic leukemia Current oncology reports 2008;10:363-364 [Abstract/editorial/review/comments]
549.	Xu,W. et al.Comparison between efficacy and safety of rituximab plus CHOP regimen and CHOP regimen for treatment of newly diagnosed patients with diffuse large B-cell lymphoma Zhongguo shi yan xue ye xue za zhi  2008;16:933-937 [Non randomized studies]
550.	Gu,W. J. et al.Treatment of chronic lymphocytic leukemia with regimen of fludarabine, cyclophosphamide and rituximab Zhongguo shi yan xue ye xue za zhi  2008;16:938-942 [Abstract/editorial/review/comments]
551.	Zent,C. S. et al.Early treatment of high-risk chronic lymphocytic leukemia with alemtuzumab and rituximab Cancer 2008;113:2110-2118 [Other MoAb included]
552.	Pro,B. et al.Phase II multicenter study of oblimersen sodium, a Bcl-2 antisense oligonucleotide, in combination with rituximab in patients with recurrent B-cell non-Hodgkin lymphoma British journal of haematology 2008;143:355-360 [Non randomized studies]
553.	Martin,A. et al.R-ESHAP as salvage therapy for patients with relapsed or refractory diffuse large B-cell lymphoma: the influence of prior exposure to rituximab on outcome. A GEL/TAMO study Haematologica 2008;93:1829-1836 [Non randomized studies]
554.	Quinn,J. P. et al.Efficacy of rituximab in combination with steroids in refractory chronic lymphocytic leukemia Leukemia & lymphoma 2008;49:1995-1998 [Abstract/editorial/review/comments]
555.	Wang,M. et al.Phase 2 trial of rituximab plus hyper-CVAD alternating with rituximab plus methotrexate-cytarabine for relapsed or refractory aggressive mantle cell lymphoma Cancer 2008;113:2734-2741 [Non R free arm]
556.	Gmeiner Stopar,T. et al.99mTc-labelled rituximab, a new non-Hodgkin's lymphoma imaging agent: first clinical experience Nuclear medicine communications 2008;29:1059-1065 [Other MoAb included]
557.	Hsi,E. D. et al.Ki67 and PIM1 expression predict outcome in mantle cell lymphoma treated with high dose therapy, stem cell transplantation and rituximab: a Cancer and Leukemia Group B 59909 correlative science study Leukemia & lymphoma 2008;49:2081-2090 [Non randomized studies]
558.	Siano,M. et al.A phase I-II study to determine the maximum tolerated infusion rate of rituximab with special emphasis on monitoring the effect of rituximab on cardiac function Clinical cancer research 2008;14:7935-7939 [Non randomized studies]
559.	Kalinka-Warzocha,E. et al.Randomized comparison of cladribine alone or in combination with cyclophosphamide, and cyclophosphamide, vincristine and prednisone in previously untreated low-grade B-cell non-Hodgkin lymphoma patients: Final report of the polish lymphoma research group. Cancer 2008;113:367-375 [Non R free arm]
560.	Sailler,L. et al.Rituximab off label use for difficult-to-treat auto-immune diseases: Reappraisal of benefits and risks. Clinical Reviews in Allergy and Immunology 2008;34:103-110 [Abstract/editorial/review/comments]
561.	Rummel,M. J. et al.Bendamustine in Chronic Lymphocytic Leukemia and Refractory Lymphoma. Seminars in hematology 2008;45:7s-10s [Abstract/editorial/review/comments]
562.	Evens,A. M. et al.Treatment of Hodgkin lymphoma: The past, present, and future. Nature Clinical Practice Oncology 2008;5:543-556 [Abstract/editorial/review/comments]
563.	Chanan-Khan,A. A. et al.Lenalidomide for the treatment of B-cell malignancies. Journal of Clinical Oncology 2008;26:1544-1552 [Abstract/editorial/review/comments]
564.	Treon,S. P. et al.Thalidomide and rituximab in Waldenstrom macroglobulinemia Blood 2008;112:4452-4457 [Non randomized studies]
565.	Witzens-Harig,M. et al.Quality of life during maintenance therapy with the anti-CD20 antibody rituximab in patients with B cell non-Hodgkin's lymphoma: Results of a prospective randomized controlled trial. Annals of Hematology 2009;88:51-57 [Purging, maintenance and sequential protocols]
566.	De Cerio,A. L. -D et al.Future of idiotypic vaccination for B-cell lymphoma. Expert Review of Vaccines 2009;8:43-50 [Abstract/editorial/review/comments]
567.	Zhang,T. et al.Combination of active specific immunotherapy or adoptive antibody or lymphocyte immunotherapy with chemotherapy in the treatment of cancer. Cancer Immunology, Immunotherapy 2009;58:475-492 [Abstract/editorial/review/comments]
568.	Copelan,E. et al.A randomized trial of etoposide and G-CSF with or without rituximab for PBSC mobilization in B-cell non-Hodgkin's lymphoma. Bone marrow transplantation 2009;43:101-105 [Purging, maintenance and sequential protocols]
569.	Rodrigues,E. B. et al.Therapeutic monoclonal antibodies in ophthalmology. Progress in retinal and eye research 2009;28:117-144 [Abstract/editorial/review/comments]
570.	Kalaycio,M. et al.Bendamustine: A new look at an old drug. Cancer 2009;115:473-479 [Abstract/editorial/review/comments]
571.	Hamblin,T. J. et al.Fludarabine, cyclophosphamide and rituximab for chronic lymphocytic leukemia: No country for old men?. Nature Clinical Practice Oncology 2009;6:130-131 [Abstract/editorial/review/comments]
572.	Korn,E. L. et al.Stopping or reporting early for positive results in randomized clinical trials: The national cancer institute cooperative group experience from 1990 to 2005. Journal of Clinical Oncology 2009;27:1712-1721 [Abstract/editorial/review/comments]
573.	Milani,C. et al.Veltuzumab, an anti-CD20 mAb for the treatment of non-Hodgkin's lymphoma, chronic lymphocytic leukemia and immune thrombocytopenic purpura. Current opinion in molecular therapeutics 2009;11:200-207 [Other MoAb included]
574.	Sharma,V. K. et al.Stem Cell Through Present and Future. Indian journal of pediatrics 2009;76:51-56 [Abstract/editorial/review/comments]
575.	Ciceri,F. et al.Infusion of suicide-gene-engineered donor lymphocytes after family haploidentical haemopoietic stem-cell transplantation for leukaemia (the TK007 trial): a non-randomised phase I-II study. The Lancet Oncology 2009;10:489-500 [Non randomized studies]
576.	Brannagan III,T. H. et al.Current treatments of chronic immune-mediated demyelinating polyneuropathies. Muscle and Nerve 2009;39:563-578 [Abstract/editorial/review/comments]
577.	Perreault,S. et al.Romiplostim: A novel thrombopoiesis-stimulating agent. American Journal of Health-System Pharmacy 2009;66:817-824 [Abstract/editorial/review/comments]
578.	Feng,J. -J et al.Meta-analysis on the efficacy and safety of R-CHOP chemotherapy for the treatment of low and moderate malignant B cell non-Hodgkin lymphoma. Journal of Leukemia and Lymphoma 2009;18:216-219 [Abstract/editorial/review/comments]
579.	Migkou,M. et al.Applications of monoclonal antibodies for the treatment of hematological malignancies. Expert Opinion on Biological Therapy 2009;9:207-220 [Abstract/editorial/review/comments]
580.	McDonald,V. et al.Rituximab in non-haematological disorders of adults and its mode of action. British journal of haematology 2009;146:233-246 [Abstract/editorial/review/comments]
581.	Aksoy,S. et al.Infectious complications of rituximab in patients with lymphoma during maintenance therapy: A systematic review and meta-analysis. Leukemia and Lymphoma 2009;50:357-365 [Abstract/editorial/review/comments]
582.	Renwick,W. et al.Use of filgrastim and pegfilgrastim to support delivery of chemotherapy: Twenty years of clinical experience. BioDrugs 2009;23:175-186 [Abstract/editorial/review/comments]
583.	Robu,D. et al.New strategies with Zevalin consolidation in the treatment of non-Hodgkin lymphomas. Medecine Nucleaire 2009;33:491-498 [Other MoAb included]
584.	Von Falck,C. et al.F-18 2-fluoro-2-deoxy-glucose positron emission tomography/computed tomography for the detection of radicular and peripheral neurolymphomatosis: Correlation with magnetic resonance imaging and ultrasound. Clinical nuclear medicine 2009;34:493-495 [Non R free arm]
585.	Geisler,C. et al.Mantle cell lymphoma: Are current therapies changing the course of disease?. Current oncology reports 2009;11:371-377 [Abstract/editorial/review/comments]
586.	Maddocks,K. J. et al.Update in the management of chronic lymphocytic leukemia.  2009;:29-ss [Abstract/editorial/review/comments]
587.	Jamshed,S. et al.Role of bendamustine in the treatment of chronic lymphocytic leukemia. OncoTargets and Therapy 2009;2:43-49 [Abstract/editorial/review/comments]
588.	Moskowitz,A. J. et al.Controversies in the treatment of lymphoma with autologous transplantation. Oncologist 2009;14:921-929 [Abstract/editorial/review/comments]
589.	Eichhorst,B. F. et al.First-line therapy with fludarabine compared with chlorambucil does not result in a major benefit for elderly patients with advanced chronic lymphocytic leukemia. Blood 2009;114:3382-3391 [Non R free arm]
590.	Zwick,C. et al.Treatment of diffuse large B-cell lymphomas. Memo - Magazine of European Medical Oncology 2009;2:150-153 [Abstract/editorial/review/comments]
591.	Hess,G. et al.Temsirolimus in Mantle Cell Lymphoma and Other Non-Hodgkin Lymphoma Subtypes. Seminars in oncology 2009;36:37s-45s [Non R free arm]
592.	Dancey,J. E. et al.Evaluating Temsirolimus Activity in Multiple Tumors: A Review of Clinical Trials. Seminars in oncology 2009;36:46s-58s [Abstract/editorial/review/comments]
593.	Coiffier,B. et al.Exploring mammalian target of rapamycin (mTOR) inhibition for treatment of mantle cell lymphoma and other hematologic malignancies. Leukemia and Lymphoma 2009;50:1916-1930 [Abstract/editorial/review/comments]
594.	Yamanaka,R. et al.Management of refractory or relapsed primary central nervous system lymphoma (review). Molecular Medicine Reports 2009;2:879-885 [Abstract/editorial/review/comments]
595.	Witzens-Harig,M. et al.Quality of life during maintenance therapy with the anti-CD20 antibody rituximab in patients with B cell non-Hodgkin's lymphoma: results of a prospective randomized controlled trial Annals of Hematology 2009;88:51-57 [Non randomized studies]
596.	Boehme,V. et al.CNS events in elderly patients with aggressive lymphoma treated with modern chemotherapy (CHOP-14) with or without rituximab: an analysis of patients treated in the RICOVER-60 trial of the German High-Grade Non-Hodgkin Lymphoma Study Group (DSHNHL) Blood 2009;113:3896-3902 [Duplicate publication]
597.	Hochster,H. et al.Maintenance rituximab after cyclophosphamide, vincristine, and prednisone prolongs progression-free survival in advanced indolent lymphoma: results of the randomized phase III ECOG1496 Study Journal of clinical oncology  2009;27:1607-1614 [Purging, maintenance and sequential protocols]
598.	Freedman,A. et al.Placebo-controlled phase III trial of patient-specific immunotherapy with mitumprotimut-T and granulocyte-macrophage colony-stimulating factor after rituximab in patients with follicular lymphoma Journal of clinical oncology  2009;27:3036-3043 [Other MoAb included]
599.	Takata,T. et al.Attenuated antibody reaction for the primary antigen but not for the recall antigen of influenza vaccination in patients with non-Hodgkin B-cell lymphoma after the administration of rituximab-CHOP Journal of clinical and experimental hematopathology  2009;49:9-13 [Non randomized studies]
600.	Schutt,P. et al.Anthracyline-reduced sequential combination chemotherapy for younger patients with good-prognosis aggressive B-cell non-Hodgkin's lymphoma Journal of cancer research and clinical oncology 2009;135:459-466 [Non R free arm]
601.	Griffin,T. C. et al.A study of rituximab and ifosfamide, carboplatin, and etoposide chemotherapy in children with recurrent/refractory B-cell (CD20+) non-Hodgkin lymphoma and mature B-cell acute lymphoblastic leukemia: a report from the Children's Oncology Group Pediatric blood & cancer 2009;52:177-181 [Non randomized studies]
602.	Chamberlain,M. C. et al.Recurrent lymphomatous meningitis treated with intra-CSF rituximab and liposomal ara-C Journal of neuro-oncology 2009;91:271-277 [Abstract/editorial/review/comments]
603.	Garbo,L. E. et al.Results of a Phase II trial of gemcitabine, mitoxantrone, and rituximab in relapsed or refractory mantle cell lymphoma Investigational new drugs 2009;27:476-481 [Non randomized studies]
604.	Treon,S. P. et al.Long-term outcomes to fludarabine and rituximab in Waldenstrom macroglobulinemia Blood 2009;113:3673-3678 [Abstract/editorial/review/comments]
605.	Tobinai,K. et al.Japanese phase II study of 90Y-ibritumomab tiuxetan in patients with relapsed or refractory indolent B-cell lymphoma Cancer science 2009;100:158-164 [Other MoAb included]
606.	Hicks,L. K. et al.Rituximab purging and maintenance combined with auto-SCT: long-term molecular remissions and prolonged hypogammaglobulinemia in relapsed follicular lymphoma Bone marrow transplantation 2009;43:701-708 [Non randomized studies]
607.	van 't Veer,M. B. et al.High-dose Ara-C and beam with autograft rescue in R-CHOP responsive mantle cell lymphoma patients British journal of haematology 2009;144:524-530 [Non randomized studies]
608.	Lin,K. I. et al.Relevance of the immunoglobulin VH somatic mutation status in patients with chronic lymphocytic leukemia treated with fludarabine, cyclophosphamide, and rituximab (FCR) or related chemoimmunotherapy regimens Blood 2009;113:3168-3171 [Abstract/editorial/review/comments]
609.	Pettengell,R. et al.Multivariate analysis of febrile neutropenia occurrence in patients with non-Hodgkin lymphoma: data from the INC-EU Prospective Observational European Neutropenia Study British journal of haematology 2009;144:677-685 [Abstract/editorial/review/comments]
610.	Illidge,T. M. et al.Phase 1/2 study of fractionated (131)I-rituximab in low-grade B-cell lymphoma: the effect of prior rituximab dosing and tumor burden on subsequent radioimmunotherapy Blood 2009;113:1412-1421 [Non R free arm]
611.	Al Zahrani,A. et al.Rapid infusion rituximab changing practice for patient care Journal of oncology pharmacy practice 2009;15:183-186 [Non R free arm]
612.	Eve,H. E. et al.Impairment of peripheral blood stem-cell mobilisation in patients with mantle-cell lymphoma following primary treatment with fludarabine and cyclophosphamide +/- rituximab Leukemia & lymphoma 2009;50:463-465 [Purging, maintenance and sequential protocols]
613.	Aviles,A. et al.Rituximab and chemotherapy in primary gastric lymphoma Cancer biotherapy & radiopharmaceuticals 2009;24:25-28 [Non randomized studies]
614.	Woyach,J. A. et al.A phase I/II study of rituximab and etanercept in patients with chronic lymphocytic leukemia and small lymphocytic lymphoma Leukemia  2009;23:912-918 [Non randomized studies]
615.	Hiraga,J. et al.Down-regulation of CD20 expression in B-cell lymphoma cells after treatment with rituximab-containing combination chemotherapies: its prevalence and clinical significance Blood 2009;113:4885-4893 [Non randomized studies]
616.	Luminari,S. et al.Anthracycline-fludarabine-containing regimens with or without rituximab in the treatment of patients with advanced follicular lymphoma Cancer 2009;115:1906-1913 [Non identical CHT in either arm]
617.	Brown,J. R. et al.A phase 2 study of concurrent fludarabine and rituximab for the treatment of marginal zone lymphomas British journal of haematology 2009;145:741-748 [Non randomized studies]
618.	Ferrajoli,A. et al.Incorporating the use of GM-CSF in the treatment of chronic lymphocytic leukemia Leukemia & lymphoma 2009;50:514-516 [Non R free arm]
619.	Weigert,O. et al.A novel regimen combining high dose cytarabine and bortezomib has activity in multiply relapsed and refractory mantle cell lymphoma - long-term results of a multicenter observation study Leukemia & lymphoma 2009;50:716-722 [Non R free arm]
620.	Shea,T. C. et al.Sequential high-dose ifosfamide, carboplatin and etoposide with rituximab for relapsed Hodgkin and large B-cell non-Hodgkin lymphoma: increased toxicity without improvement in progression-free survival Leukemia & lymphoma 2009;50:741-748 [Non identical CHT in either arm]
621.	Tam,C. S. et al.De novo deletion 17p13.1 chronic lymphocytic leukemia shows significant clinical heterogeneity: the M. D. Anderson and Mayo Clinic experience Blood 2009;114:957-964 [Non R free arm]
622.	Mishima,Y. et al.An imaging-based rapid evaluation method for complement-dependent cytotoxicity discriminated clinical response to rituximab-containing chemotherapy Clinical cancer research 2009;15:3624-3632 [Non R free arm]
623.	Rasmussen,P. et al.Mantle cell lymphoma in the orbital and adnexal region The British journal of ophthalmology 2009;93:1047-1051 [Non randomized studies]
624.	Tobinai,K. et al.Phase I/II and pharmacokinetic study of cladribine with 2-h infusion in Japanese patients with relapsed indolent B-cell lymphoma mostly pretreated with rituximab Cancer science 2009;100:1344-1350 [Non R free arm]
625.	Morschhauser,F. et al.Humanized anti-CD20 antibody, veltuzumab, in refractory/recurrent non-Hodgkin's lymphoma: phase I/II results Journal of clinical oncology 2009;27:3346-3353 [Other MoAb included]
626.	Friedberg,J. W. et al.Phase II study of a TLR-9 agonist (1018 ISS) with rituximab in patients with relapsed or refractory follicular lymphoma British journal of haematology 2009;146:282-291 [Non randomized studies]
627.	Hainsworth,J. D. et al.Rituximab plus short-duration chemotherapy followed by Yttrium-90 Ibritumomab tiuxetan as first-line treatment for patients with follicular non-Hodgkin lymphoma: a phase II trial of the Sarah Cannon Oncology Research Consortium Clinical lymphoma & myeloma 2009;9:223-228 [Other MoAb included]
628.	Xia,Y. et al.Short-term efficacy of rituximab-CHOP and CHOP regimens on two subtypes of diffuse large B-cell lymphoma Ai zheng  2009;28:146-149 [Chinese language]
629.	Haioun,C. et al.Rituximab versus observation after high-dose consolidative first-line chemotherapy with autologous stem-cell transplantation in patients with poor-risk diffuse large B-cell lymphoma Annals of Oncology 2009;20:1985-1992 [Purging, maintenance and sequential protocols]
630.	Vitolo,U. et al.Dose-dense and high-dose chemotherapy plus rituximab with autologous stem cell transplantation for primary treatment of diffuse large B-cell lymphoma with a poor prognosis: a phase II multicenter study Haematologica 2009;94:1250-1258 [Non randomized studies]
631.	Ribrag,V. et al.Efficacy and toxicity of 2 schedules of frontline rituximab plus cyclophosphamide, doxorubicin, vincristine, and prednisone plus bortezomib in patients with B-cell lymphoma: a randomized phase 2 trial from the French Adult Lymphoma Study Group (GELA) Cancer 2009;115:4540-4546 [Non randomized studies]
632.	Tobinai,K. et al.Phase II study of oral fludarabine in combination with rituximab for relapsed indolent B-cell non-Hodgkin lymphoma Cancer science 2009;100:1951-1956 [Non randomized studies]
633.	Bottcher,S. et al.Standardized MRD flow and ASO IGH RQ-PCR for MRD quantification in CLL patients after rituximab-containing immunochemotherapy: a comparative analysis Leukemia 2009;23:2007-2017 [Non R free arm]
634.	Salar,A. et al.Combination therapy with rituximab and intravenous or oral fludarabine in the first-line, systemic treatment of patients with extranodal marginal zone B-cell lymphoma of the mucosa-associated lymphoid tissue type Cancer 2009;115:5210-5217 [Non randomized studies]
635.	Bosch,F. et al.Rituximab, fludarabine, cyclophosphamide, and mitoxantrone: a new, highly active chemoimmunotherapy regimen for chronic lymphocytic leukemia Journal of clinical oncology  2009;27:4578-4584 [Non randomized studies]
636.	Klepfish,A. et al.Enhancing the action of rituximab in chronic lymphocytic leukemia by adding fresh frozen plasma: complement/rituximab interactions & clinical results in refractory CLL Annals of the New York Academy of Sciences 2009;1173:865-873 [Non R free arm]
637.	Wang,M. et al.Phase II study of yttrium-90-ibritumomab tiuxetan in patients with relapsed or refractory mantle cell lymphoma Journal of clinical oncology  2009;27:5213-5218 [Other MoAb included]
638.	de Vos,S. et al.Multicenter randomized phase II study of weekly or twice-weekly bortezomib plus rituximab in patients with relapsed or refractory follicular or marginal-zone B-cell lymphoma Journal of clinical oncology  2009;27:5023-5030 [Non R free arm]
639.	Copie-Bergman,C. et al.Immuno-fluorescence in situ hybridization index predicts survival in patients with diffuse large B-cell lymphoma treated with R-CHOP: a GELA study Journal of clinical oncology  2009;27:5573-5579 [Non R free arm]
640.	Derenzini,E. et al.Cyclophosphamide, doxorubicin, vincristine, methotrexate, bleomicin and prednisone plus rituximab in untreated young patients with low-risk (age-adjusted international prognostic index 0-1) diffuse large B-cell lymphoma Leukemia & lymphoma 2009;50:1824-1829 [Non R free arm]
641.	Evens,A. M. et al.The novel expanded porphyrin, motexafin gadolinium, combined with [90Y]ibritumomab tiuxetan for relapsed/refractory non-Hodgkin's lymphoma: preclinical findings and results of a phase I trial Clinical cancer research  2009;15:6462-6471 [Other MoAb included]
642.	Delaloye,A. B. et al.Dosimetry of 90Y-ibritumomab tiuxetan as consolidation of first remission in advanced-stage follicular lymphoma: results from the international phase 3 first-line indolent trial Journal of nuclear medicine  2009;50:1837-1843 [Other MoAb included]
643.	Aviles,A. et al.Rituximab and dose-dense chemotherapy in primary testicular lymphoma Clinical lymphoma & myeloma 2009;9:386-389 [Non randomized studies]
644.	Schneider,T. et al.Results of immuno-chemotherapeutic treatment of patients with diffuse large B-cell lymphoma Orvosi hetilap 2009;150:2019-2026 [Non randomized studies]
645.	Duhrsen,U. et al.Positron emission tomography guided therapy of aggressive non-Hodgkin lymphomas--the PETAL trial Leukemia & lymphoma 2009;50:1757-1760 [No infection outcome]
646.	Damon,L. E. et al.Immunochemotherapy and autologous stem-cell transplantation for untreated patients with mantle-cell lymphoma: CALGB 59909 Journal of clinical oncology  2009;27:6101-6108 [Non randomized studies]
647.	Barr,P. M. et al.Phase I trial of fludarabine, bortezomib and rituximab for relapsed and refractory indolent and mantle cell non-Hodgkin lymphoma British journal of haematology 2009;147:89-96 [Non R free arm]
648.	Blasco,H. et al.Pharmacokinetics of rituximab associated with CHOP chemotherapy in B-cell non-Hodgkin lymphoma Fundamental & clinical pharmacology 2009;23:601-608 [Non randomized studies]
649.	Blum,K. A. et al.Phase II study of the histone deacetylase inhibitor MGCD0103 in patients with previously treated chronic lymphocytic leukaemia British journal of haematology 2009;147:507-514 [Non R free arm]
650.	Cheson,B. D. et al.Bendamustine and rituximab - Optimizing dose and schedule. Clinical Advances in Hematology and Oncology 2009;7:3-5 [Abstract/editorial/review/comments]
651.	Dennie,T. W. et al.Bendamustine for the treatment of chronic lymphocytic leukemia and rituximab-refractory, indolent B-cell non-hodgkin lymphoma. Clinical therapeutics 2009;31:2290-2311 [Non R free arm]
652.	Marinella,M. A. et al.Reversible posterior leucoencephalopathy syndrome associated with anticancer drugs. Internal Medicine Journal 2009;39:826-834 [Abstract/editorial/review/comments]
653.	Robak,T. et al.Current and emerging treatments for chronic lymphocytic leukaemia. Drugs 2009;69:2415-2449 [Abstract/editorial/review/comments]
654.	Schlette,E. J. et al.P53 Expression by Immunohistochemistry is an Important Determinant of Survival in Patients with Chronic Lymphocytic Leukemia Receiving Frontline Chemo-Immunotherapy Leukemia & lymphoma 2009;50:1597-1605 [Abstract/editorial/review/comments]
655.	Steurer,M. et al.Chronic lymphocytic leukaemia. Memo - Magazine of European Medical Oncology 2009;2:103- [Abstract/editorial/review/comments]
656.	Tedeschi,A. et al.Fludarabine-based combination therapies for Waldenstrom's macroglobulinemia. Clinical lymphoma & myeloma 2009;9:67-70 [Abstract/editorial/review/comments]
657.	Tsimberidou,A. -M et al.Ultimate fate of oncology drugs approved by the US food and drug administration without a randomized trial. Journal of Clinical Oncology 2009;27:6243-6250 [Abstract/editorial/review/comments]
658.	Vleugels,R. A. et al.Dermatomyositis: Current and future treatments. Expert Review of Dermatology 2009;4:581-594 [Abstract/editorial/review/comments]
659.	Gertz,M. A. et al.Clinical value of minor responses after 4 doses of rituximab in Waldenstrom macroglobulinaemia: a follow-up of the Eastern Cooperative Oncology Group E3A98 trial British journal of haematology 2009;147:677-680 [Non randomized studies]
660.	Treon,S. P. et al.Primary therapy of Waldenstrom macroglobulinemia with bortezomib, dexamethasone, and rituximab: WMCTG clinical trial 05-180 Journal of clinical oncology  2009;27:3830-3835 [Non R free arm]
661.	Treon,S. P. et al.Lenalidomide and rituximab in Waldenstrom's macroglobulinemia Clinical cancer research  2009;15:355-360 [Non randomized studies]
662.	Shustik,J. et al.Correlations between BCL6 rearrangement and outcome in patients with diffuse large B-cell lymphoma treated with CHOP or R-CHOP. Haematologica 2010;95:96-101 [Abstract/editorial/review/comments]
663.	Lin,T. S. et al.Flavopiridol, fludarabine, and rituximab in mantle cell lymphoma and indolent B-cell lymphoproliferative disorders Journal of clinical oncology  2010;28:418-423 [Non randomized studies]
664.	Aue,G. et al.Fractionated subcutaneous rituximab is well-tolerated and preserves CD20 expression on tumor cells in patients with chronic lymphocytic leukemia Haematologica 2010;95:329-332 [Non R free arm]
665.	Di Bella,N. et al.Results of a phase 2 study of bortezomib in patients with relapsed or refractory indolent lymphoma Blood 2010;115:475-480 [Non R free arm]
666.	Gao,G. et al.A systematic review and meta-analysis of immunochemotherapy with rituximab for B-cell non-Hodgkin's lymphoma. Acta Oncologica 2010;49:3-12 [Abstract/editorial/review/comments]
667.	Horning,S. J. et al.Interim positron emission tomography scans in diffuse large B-cell lymphoma: An independent expert nuclear medicine evaluation of the Eastern Cooperative Oncology Group E3404 study. Blood 2010;115:775-777 [Non R free arm]
668.	Kahl,B. S. et al.Bendamustine is effective therapy in patients with rituximab-refractory, indolent B-cell non-Hodgkin lymphoma: results from a Multicenter Study Cancer 2010;116:106-114 [Non R free arm]
669.	Kotake,T. et al.Intravascular large B-cell lymphoma presenting pulmonary arterial hypertension as an initial manifestation. Internal Medicine 2010;49:51-54 [Abstract/editorial/review/comments]
670.	Bachy,E. et al.Long-term follow-up of patients with newly diagnosed follicular lymphoma in the prerituximab era: effect of response quality on survival--A study from the groupe d'etude des lymphomes de l'adulte Journal of Clinical Oncology 2010;28:822-829 [Non randomized studies]
671.	Laatiri,M. A. et al.Tunisian experience in the treatment of aggressive non Hodgkin's lymphoma in adults: About 337 patients. Bulletin du cancer 2010;97:409-416 [Non randomized studies]
672.	Lamanna,N. et al.Challenges in the frontline treatment of patients with chronic lymphocytic leukemia. Current Hematologic Malignancy Reports 2010;5:45-51 [Abstract/editorial/review/comments]
673.	Levy,M. et al.Treatment of t(11;18)-positive gastric mucosa-associated lymphoid tissue lymphoma with rituximab and chlorambucil: clinical, histological, and molecular follow-up Leukemia & lymphoma 2010;51:284-290 [Non randomized studies]
674.	Lucas,G. et al.Recipient-derived HPA-1a antibodies: A cause of prolonged thrombocytopenia after unrelated donor stem cell transplantation. Transfusion 2010;50:334-339 [Abstract/editorial/review/comments]
675.	Machover,D. et al.Treatment with rituximab, dexamethasone, high-dose cytarabine, and oxaliplatin (R-DHAOx) produces a strong long-term antitumor effect in previously treated patients with follicular non-Hodgkin's lymphoma Biomedicine & pharmacotherapy 2010;64:83-87 [Non randomized studies]
676.	Niitsu,N. et al.Multicentre phase II study of CyclOBEAP plus rituximab in patients with diffuse large B-cell lymphoma Hematological oncology 2010;28:68-74 [Non randomized studies]
677.	Murawski,N. et al.Unresolved issues in diffuse large B-cell lymphomas. Expert Review of Anticancer Therapy 2010;10:387-402 [Abstract/editorial/review/comments]
678.	Orciuolo,E. et al.2CdA chemotherapy and rituximab in the treatment of marginal zone lymphoma Leukemia research 2010;34:184-189 [Non randomized studies]
679.	Qiao,W. et al.Predictive value of (18)F-FDG hybrid PET/CT for the clinical outcome in patients with non-Hodgkin's lymphoma prior to and after autologous stem cell transplantation Hematology (Amsterdam, Netherlands) 2010;15:21-27 [Non R free arm]
680.	Bacigalupo,A. et al.Pre-emptive treatment of acute GVHD: A randomized multicenter trial of rabbit anti-thymocyte globulin, given on day7 after alternative donor transplants. Bone marrow transplantation 2010;45:385-391 [Other MoAb included]
681.	Rabascio,C. et al.Expression of the human concentrative nucleotide transporter 1 (hCNT1) gene correlates with clinical response in patients affected by Waldenstrom's Macroglobulinemia (WM) and small lymphocytic lymphoma (SLL) undergoing a combination treatment with 2-chlor Leukemia research 2010;34:454-457 [Non randomized studies]
682.	Ruan,J. et al.Durable responses with the metronomic rituximab and thalidomide plus prednisone, etoposide, procarbazine, and cyclophosphamide regimen in elderly patients with recurrent mantle cell lymphoma Cancer 2010;116:2655-2664 [Non R free arm]
683.	Sissolak,G. et al.Human immunodeficiency and Hodgkin lymphoma. Transfusion and Apheresis Science 2010;42:131-139 [Abstract/editorial/review/comments]
684.	Sparano,J. A. et al.Rituximab plus concurrent infusional EPOCH chemotherapy is highly effective in HIV-associated B-cell non-Hodgkin lymphoma Blood 2010;115:3008-3016 [Non R free arm]
685.	Takasaki,H. et al.Clinical study of sequential high-dose chemotherapy with in vivo rituximab-purged stem cell autografting for mantle cell lymphoma [Rinsho ketsueki] The Japanese journal of clinical hematology 2010;51:57-62 [Abstract/editorial/review/comments]
686.	Yoshikawa,T. et al.Serum concentration of L-kynurenine predicts the clinical outcome of patients with diffuse large B-cell lymphoma treated with R-CHOP European journal of haematology 2010;84:304-309 [Non R free arm]
687.	Ziepert,M. et al.Standard International prognostic index remains a valid predictor of outcome for patients with aggressive CD20+ B-cell lymphoma in the rituximab era Journal of Clinical Oncology 2010;28:2373-2380 [Abstract/editorial/review/comments]
688.	Zwick,C. et al.Rituximab in high-grade lymphoma. Seminars in hematology 2010;47:148-155 [Abstract/editorial/review/comments]
689.	Bendandi,M. et al.Hybridoma-derived idiotype vaccine for lymphoma: Approval must wait. Pharmaceuticals 2010;3:667-678 [Non R free arm]
690.	Anonimous et al.Rituximab: Chronic lymphoid leukaemia: No decisive advantage. Prescrire international 2010;19:56-58 [Abstract/editorial/review/comments]
691.	Byrd,J. C. et al.Phase 1/2 study of lumiliximab combined with fludarabine, cyclophosphamide, and rituximab in patients with relapsed or refractory chronic lymphocytic leukemia Blood 2010;115:489-495 [Other MoAb included]
692.	Caimi,P. F. et al.Non-hodgkins lymphoma in the elderly. Drugs and Aging 2010;27:211-238 [Abstract/editorial/review/comments]
693.	Capitini,C. M. et al.Immune-based therapeutics for pediatric cancer. Expert Opinion on Biological Therapy 2010;10:163-178 [Children (aged 16 or less)]
694.	Carrabba,M. G. et al.Treatment approaches for primary CNS lymphomas. Expert opinion on pharmacotherapy 2010;11:1263-1276 [Abstract/editorial/review/comments]
695.	Cavattoni,I. et al.Pilot study of rituximab plus donor-lymphocyte infusion to prevent or treat relapse in B-cell lymphoma after allogeneic stem cell transplantation Leukemia & lymphoma 2010;51:146-148 [Non R free arm]
696.	Niitsu,N. et al.Multicentre phase II study of CyclOBEAP plus rituximab in patients with diffuse large B-cell lymphoma Hematological oncology 2010;28:68-74 [Non randomized studies]
697.	Pott,C. et al.Molecular remission is an independent predictor of clinical outcome in patients with mantle cell lymphoma after combined immunochemotherapy: a European MCL intergroup study Blood 2010;115:3215-3223 [No infection outcome]
698.	Rabascio,C. et al.Expression of the human concentrative nucleotide transporter 1 (hCNT1) gene correlates with clinical response in patients affected by Waldenstrom's Macroglobulinemia (WM) and small lymphocytic lymphoma (SLL) undergoing a combination treatment with 2-chlor Leukemia research 2010;34:454-457 [Non R free arm]
699.	Sparano,J. A. et al.Rituximab plus concurrent infusional EPOCH chemotherapy is highly effective in HIV-associated B-cell non-Hodgkin lymphoma Blood 2010;115:3008-3016 [Non randomized studies]
700.	Tsirigotis,P. et al.Post-autologous stem cell transplantation administration of rituximab improves the outcome of patients with aggressive B cell non-Hodgkin's lymphoma Annals of Hematology 2010;89:263-272 [Non randomized studies]
701.	Bachy,E. et al.Long-term follow-up of patients with newly diagnosed follicular lymphoma in the prerituximab era: effect of response quality on survival--A study from the groupe d'etude des lymphomes de l'adulte Journal of clinical oncology  2010;28:822-829 [Non randomized studies]
702.	Dunleavy,K. et al.The role of tumor histogenesis, FDG-PET, and short-course EPOCH with dose-dense rituximab (SC-EPOCH-RR) in HIV-associated diffuse large B-cell lymphoma Blood 2010;115:3017-3024 [Non randomized studies]
703.	Faderl,S. et al.Alemtuzumab by continuous intravenous infusion followed by subcutaneous injection plus rituximab in the treatment of patients with chronic lymphocytic leukemia recurrence Cancer 2010;116:2360-2365 [Other MoAb included]
704.	Faderl,S. et al.Fludarabine, cyclophosphamide, mitoxantrone plus rituximab (FCM-R) in frontline CLL <70 Years Leukemia research 2010;34:284-288 [Non randomized studies]
705.	Hainsworth,J. D. et al.Brief-duration rituximab/chemotherapy followed by maintenance rituximab in patients with diffuse large B-cell lymphoma who are poor candidates for R-CHOP chemotherapy: a phase II trial of the Sarah Cannon Oncology Research Consortium Clinical lymphoma, myeloma & leukemia 2010;10:44-50 [Non R free arm]
706.	Kay,N. E. et al.Pentostatin and rituximab therapy for previously untreated patients with B-cell chronic lymphocytic leukemia Cancer 2010;116:2180-2187 [Non randomized studies]
707.	Koc,O. N. et al.A phase 2 trial of immunotherapy with mitumprotimut-T (Id-KLH) and GM-CSF following rituximab in follicular B-cell lymphoma Journal of immunotherapy (Hagerstown, Md. 2010;33:178-184 [Other MoAb included]
708.	Levy,M. et al.Treatment of t(11;18)-positive gastric mucosa-associated lymphoid tissue lymphoma with rituximab and chlorambucil: clinical, histological, and molecular follow-up Leukemia & lymphoma 2010;51:284-290 [Abstract/editorial/review/comments]
709.	Perkins,J. et al.A Randomized Phase II Trial Comparing Tacrolimus and Mycophenolate Mofetil to Tacrolimus and Methotrexate for Acute Graft-versus-Host Disease Prophylaxis. Biology of Blood and Marrow Transplantation 2010;16:937-947 [Abstract/editorial/review/comments]
710.	Zucca,E. et al.Cancer Convention Lugano of the European School of Oncology, ECCLU European Journal of Cancer, Supplement 2010;0:0-0 [Abstract/editorial/review/comments]
711.	Ghobrial,I. M. et al.Phase II trial of weekly bortezomib in combination with rituximab in relapsed or relapsed and refractory Waldenstrom macroglobulinemia Journal of clinical oncology  2010;28:1422-1428 [Non randomized studies]
712.	Ghobrial,I. M. et al.Clinical and translational studies of a phase II trial of the novel oral Akt inhibitor perifosine in relapsed or relapsed/refractory Waldenstrom's macroglobulinemia Clinical cancer research  2010;16:1033-1041 [Non randomized studies]
713.	Laszlo,D. et al.Rituximab and subcutaneous 2-chloro-2'-deoxyadenosine combination treatment for patients with Waldenstrom macroglobulinemia: clinical and biologic results of a phase II multicenter study Journal of clinical oncology  2010;28:2233-2238 [NON RANDOMIZED STUDIES
