# Supplementary material for: Risk of infection in patients with lymphoma receiving rituximab: systematic review and meta-analysis
Source: BMC Med. 2011 Apr 12;9:36. doi: 10.1186/1741-7015-9-36 (PMC3094236; doi:10.1186/1741-7015-9-36)
Supplement: Additional file 6 — Protocols. Details of therapies in each different RTC. [file 1741-7015-9-36-S6.RTF]

Appendix 7: details of treatments
CHOP 21	
Study	CHT PROTOCOL	RITUXIMAB 
(375 mg/m2)	ANCILLARY THERAPY	
	cycle	C 
(mg/m2)	H
(mg/m2)	O
(mg/m2)	P
 (mg/m2)		G-CSF	RTX	AI profilax.	other	
Coiffer 2002	8 	750	50	1.4	40	8 courses	Discretionary	no	no	none	
Kaplan 2005	3 or 6 depending on diseases stage	750	50	1.4	100	3 or 6 courses
(same as CHT)	All	Some	PCP (all)	MP
ARV	
Habermann 2006	6 to 8 (depending on response) 	750	50	1.4	100	4 or 5 courses 
(depending on CHT)	Discretionary	no	no	MP	
Lenz 2005	6	750	50	1.4	100	6 courses 	NR	no	no	none	
Hiddeman 2005	6 to 8 (depending on response) 	750	50	1.4	100	6 to 8 courses 
(same as CHT)	NR	no	no	none	
Van Oers 2006	3 or 6 (depending on response)	750	50	1.4	100	3 or 6 courses 
(same as CHT)	NR	no	no	none	
Buske 2009	4 to 8 (depending on response)	750	50	1.4	100	4 to 8
(same as CHT)	NR	NR	NR	none	
CHOP 14	
ID	CHT PROTOCOL	RITUXIMAB 
(375 mg/m2)	ANCILLARY THERAPY	
	cycle	C
(mg/m2)	H
(mg/m2)	O
(mg/m2)	P
(mg/m2)		G-CSF	RTX	AI profilax.	other	
Pfreundschuh 2008a	6 	750	50	2	100	   8 courses (2 additional courses after CHT)	All	no	no	Pre-O	
Pfreundschuh 2008b	8 	750	50	2	100	   8 courses	All	no	no	Pre-O	
C= Cyclophosphamide; H= doxorubicin; O=vincristine; P=prednisone; G-CSF=granulocyte colony stimulating factor; RTX= radio therapy; AI prophylax.= anti-infective prophylaxis; MP=meningeal prophylaxis with methotrexate; ART=antiretroviral therapy; PCP; prophylaxis against P.jaroviecy ; NR=not reported; PreO: pre-treatment with single dose 1 mg vincristine and 100 mg prednisone orally for 7 days.

MCP 28	
ID	CHT PROTOCOL	RITUXIMAB 
(375 mg/m2)	ANCILLARY THERAPY	
	cycle	M 
(mg/m2)	Ch
(mg/m2)	P 
(mg/m2)		G-CSF	RTX	AI profilax.	other	
Herold 2007	8	8	9	25	8 courses	NR	no	no	none	
FCM	
ID	CHT PROTOCOL	RITUXIMAB 
(375 mg/m2)
	ANCILLARY THERAPY	
	cycle	F 
(mg/m2)	C
(mg/m2)	M 
(mg/m2)		G-CSF	RTX	AI profilax.	other	
Forstpointner 2004	4	25	200	8	4 courses	NR	no	no	none	
FC	
ID	CHT PROTOCOL	RITUXIMAB 
(375 mg/m2)	ANCILLARY THERAPY	
	cycle	F 
(mg/m2)	C 
(mg/m2)		G-CSF	RTX	AI profilax.	other	
Robak 2010	6	25	250	6 courses	Discretionary	no	no	none	
Eve 2009	8	40	250	8 courses	NR	no	PCP 
VZV	none	
C= Cyclophosphamide; Ch= chlorambucil; F= Fludarabine M=Mitoxantrone; P=prednisone; G-CSF=granulocyte colony stimulating factor; RTX= radio therapy; AI prophylax.= anti-infective prophylaxis; VZV= prophylaxis against Varicella zoster virus; PCP=prophylaxis against P.jaroviecy ; NR=not reported

CEOP 	
Study	CHT PROTOCOL	RITUXIMAB 
(375 mg/m2)	ANCILLARY THERAPY	
	cycle	C 
(mg/m2)	E
(mg/m2)	O 
(mg/m2)	P 
(mg/m2)		G-CSF	RTX	AI profilax.	other	
Aviles 2007b	6 
every 14 days	1500	120	1.4	100	6 courses	NR	no	no	none	
Aviles 2007a	6
every 21 days	750	70 (1st cycle), 90 (2nd cycle), 105(3rd to 6th cycle)	2	100	6 courses	Discretionary	no	no	none	
CVP 21	
Study	cycle	C 
(mg x 5d)	V 
(mg x 5d)	P 
(mg/m2)	RITUXIMAB 
(375 mg/m2)	G-CSF	RTX	AI profilax.	other	
Marcus 2005	8	750	1.4	40	8 courses	NR	no	no	none	
ESHAP	
paper	CHT PROTOCOL	RITUXIMAB (mg/sqm)	ANCILLARY THERAPY	
	cycle	Ep	S (mg/kg)	HA (mg/sqm)	Cis-P (mg)		G-CSF	RTX	AI profilax.	other	
Aviles 2010	6	not reported	not reported	not reported	not reported	375 (each cycle) 	yes (all)	no	none	none	
C= Cyclophosphamide; E= epirobucin; O=vincristine; P=prednisone; G-CSF=granulocyte colony stimulating factor; RTX= radio therapy; AI prophylax.= anti-infective prophylaxis; Ep= etoposide; S= methylprednisolone; HA= high dose cytarabine; Cis-P=cisplatin
