# Supplementary material for: Risk of infection in patients with lymphoma receiving rituximab: systematic review and meta-analysis
Source: BMC Med. 2011 Apr 12;9:36. doi: 10.1186/1741-7015-9-36 (PMC3094236; doi:10.1186/1741-7015-9-36)
Supplement: Additional file 7 — Reported outcome. Details of studied outcomes as reported in each different RTC. [file 1741-7015-9-36-S7.RTF]

Appendix 6: This table describes the studies according to the outcome reported. 
Studies characteristics (reported outcomes)	
Study	Incidence of infection a	Diagnosis of infection 	Infection related deaths 	Granulocytopenia a	Leucopenia a	Lymphopenia a 	Overall response	
Aviles 2007a	grade not reported	not explained	not reported	per cycle	not reported	not reported	only CR	
Aviles 2007b	per cycle	not explained	reported (none)	not reported	per cycle	not reported	only CR 	
Aviles 2010	per cycle	sepsis, pneumonia, FN, viral and bacterial isolates	reported (none)	per cycle	not reported	not reported	reported *	
Buske 2009	per patient *	not explained	reported (none)	per patient *	per patient *	not reported	reported*	
Coiffier 2002	per patient *	herpes	not reported	grade not reported	not reported	not reported	reported *	
Eve 2009	per patient *	not explained	not reported	per patient *	per patient *	not reported	not reported	
Forstpointner 2004	per cycle	not explained	not reported	per cycle	per cycle	per cycle	reported *	
Habermann 2006	per patient *	not explained	reported * 	per patient *	not reported	not reported	reported *	
Herold 2007	per patient *	not explained	not reported	not reported	per patient *	not reported	reported *	
Hiddemann 2005	per cycle	not explained	not reported	not reported	not reported	not reported	reported *	
Kaplan 2005	infections during therapy and follow-up reported together	OI and FN	reported *	per patient *	not reported	not reported	reported *	
Lenz 2005	per patient *	not explained	not reported	per patient *	per patient *	not reported	reported *	
Marcus 2005	per patient *	not explained	reported (none)	per patient *	per patient *	not reported	reported  *	
Pfreundschuh 2008a	per patient *	not explained	not reported	not reported	per patient *	not reported	reported *	
Pfreundschuh 2008b	per patient *	not explained	not reported	not reported	per patient *	not reported	reported *	
Robak 2010	per patient *	FN, HBV and pneumonia	not reported	per patient *	per patient *	not reported	reported *	
van Oers 2006	only lethal infections while in therapy	only lethal infections	reported *	per patient *	not reported	not reported	reported *	
a) Grade 3 and 4 events according NCI classification; per patient= reported as number/proportion of patients experiencing at least one event during therapy; per cycle= reported as number or proportion of cycles in which occurred one or more events. FN= febrile neutropenia, OI= opportunistic infection; Only CR=reported only complete remission. * Included in the meta-analysis
