# Supplementary material for: Risk of infection in patients with lymphoma receiving rituximab: systematic review and meta-analysis
Source: BMC Med. 2011 Apr 12;9:36. doi: 10.1186/1741-7015-9-36 (PMC3094236; doi:10.1186/1741-7015-9-36)
Supplement: Additional file 8 — Within study risk of bias. Details of the studies according to within risk of bias of each different RTC. [file 1741-7015-9-36-S8.RTF]

Appendix 8: analysis of within-study risk of bias (extracted data)
Study	Sequence generation	Allocation concealment	Blnding	Incomplete outcome	Funding	Early stop	Overall
risk of bias	
	type	judgment	type	judgment	type	judgment	type	judgment	Type	judgment	type	judgment		
Aviles 2007a	SE	inadequate	SOE	adequate	none	inadequate	AT	inadequate	non-profit	adequate	none	adequate	High	
Aviles 2007b	SE	inadequate	SOE	adequate	none	inadequate	ITT	adequate	non-profit	adequate	none	adequate	High	
Aviles 2010	NR	unclear	NR	unclear	none	inadequate	ITT	adequate	non-private	adequate	none	adequate	High	
Buske 2009	BR	adequate	C	adequate	none	inadequate	ATA	inadequate	CI NR	unclear	none	adequate	High	
Coiffier 2002	NR	unclear	C	adequate	none	inadequate	AT	inadequate	manufacturer	inadequate	none	adequate	High	
Eve 2009	NR	unclear	NR	unclear	none	inadequate	ATA	inadequate	CI NR	unclear	none	adequate	High	
Forstpointner 2004	BR	adequate	C	adequate	none	inadequate	ATA	inadequate	CI NR	unclear	occurred	inadequate	High	
Habermann 2006	SR	adequate	NR	unclear	none	inadequate	ITT	adequate	manufacturer	inadequate	none	adequate	High	
Herold 2007	SR	adequate	C	adequate	none	inadequate	AT	inadequate	manufacturer	inadequate	occurred	inadequate	High	
Hiddemann 2005	SR	adequate	C	adequate	none	inadequate	ATA	inadequate	CI NR	unclear	occurred	inadequate	High	
Kaplan 2005	BR	adequate	C	adequate	none	inadequate	AT	inadequate	manufacturer	inadequate	none	adequate	High	
Lenz 2005	SR	adequate	C	adequate	none	inadequate	ATA	inadequate	manufacturer	inadequate	none	adequate	High	
Marcus 2005	SR	adequate	C	adequate	none	inadequate	AT	inadequate	manufacturer	inadequate	none	adequate	High	
Pfreundschuh 2008a	M	adequate	C	adequate	none	inadequate	ITT	adequate	manufacturer	inadequate	occurred	inadequate	High	
Pfreundschuh 2008b	M	adequate	C	adequate	none	inadequate	ITT	adequate	manufacturer	inadequate	occurred	inadequate	High	
Robak 2010	SR	adequate	NR	unclear	none	inadequate	AT	adequate	manufacturer	inadequate	none	adequate	High	
van Oers 2006	M	adequate	C	adequate	none	inadequate	AT	inadequate	non-profit	adequate	occurred	inadequate	High	
NR= not reported; BR=block randomization; SR=stratified randomization; SE= randomization by shuffling opaque sealed envelope; M= minimization; SOE=allocation concealment obtained by sealed opaque envelopes; AT= attrition bias; ATA= as treated analisys; ITT= intent to treat analysis (in absence of attrition bias); CI NR = individual conflict of interests not clearly reported (in case that R manufacturer's did not found the study). 
