# Supplementary material for: Risk of infection in patients with lymphoma receiving rituximab: systematic review and meta-analysis
Source: BMC Med. 2011 Apr 12;9:36. doi: 10.1186/1741-7015-9-36 (PMC3094236; doi:10.1186/1741-7015-9-36)
Supplement: Additional file 9 — Sensitivity analysis. Summary of complete results of sensitivity analysis. [file 1741-7015-9-36-S9.RTF]

Appendix 9: sensitivity analysis complete results

	Overall 	Randomization	Funding 	Early stop 	ITT only 	
	N	RR (95%CI)	p	N	RR (95%CI)		N	RR(95%CI)	p	N	RR(95%CI)	p	N.	RR (95%CI)	p	
Infections	10	1.00  
(0.87-1.14)	0.943	6	1.03
(0.87-1.22)	0.705	0	-	-	7	0.92 
(0.75-1.14)	0.438	4	1.04 
(0.90-1.20)	0.603	
Death for infection	3	1.60 
(0.68-3.75)	0.279	2	3.46
( 0.68-17.56)      	0.143	1	0.99
(0.06-15.69)	0.993	2	1.68 
(0.6-4.12)	0.254	1	1.19 
(0.44-1.25)	0.728	
Febrile  neutropenia	2	1.14
(0.80-1.63)	0.478	1	1.33       
(0.75-  2.36)	0.329	0	-	-	2	1.14 
(0.80-1.63)	0.478	1	1.03
 (0.65-1.63) 	0.895	
Leucopenia	8	1.24
(1.12-1.37)	<0.001	6	1.19
(1.07- 1.33)  	0.001	1	2.00  C
(1.34-2.99)	0.001	5	1.35 B
(1.16-1.58)	<0.001	3	1.23 
(1.06-1.43)	0.007	
Granulocytopenia	8	1.07
(1.02-1.12)	0.008	5	1.19 A
(1.05-1.35)	0.007	1	1.09  
(0.91-1.31)	0.331	7	1.07 
(1.01-1.12)	0.013	2	1.04
(0.99-1.10) 	0.116	
Overall response	14	1.12
(1.09-1.15)	<0.001	10	1.12
(1.08-1.16)	<0.001	2	1.16
(1.05-1.27)	0.002	8	1.17 
(1.11-1.23)	<0.001	5	1.07 
(1.02-1.12)	0.007	
In bold RR change >10% when p<0.10. In particular:
A)	RR for granulocytopenia shows a moderate increase when only studies with adequate randomization where considered. 
B)	RR for leucopenia shows a moderate increase when only studies without early stopping were considered.
C)	Variation of RR leucopenia in case of adequate funding is to be taken with caution only one study considered.
Given the size and sense (i.e. no association was reversed) of those variations I believe sensitivity analysis).
